# PARP1 activation increases expression of modified tumor suppressors and pathways underlying development of aggressive hepatoblastoma

**DOI:** 10.1038/s42003-018-0077-8

**Published:** 2018-06-11

**Authors:** Leila Valanejad, Ashley Cast, Mary Wright, Karl-Dimiter Bissig, Rebekah Karns, Matthew T. Weirauch, Nikolai Timchenko

**Affiliations:** 1 Department of Pediatric Surgery, Cincinnati Children’s Hospital Medical Center, Cincinnati, 45229 OH USA; 2 Center for Cell and Gene Therapy, Baylor College of Medicine, Houston, 77030 TX USA; 3 Department of Gastroenterology, Cincinnati Children’s Hospital Medical Center, Cincinnati, 45229 OH USA; 4 Center for Autoimmune Genomics and Etiology, Cincinnati Children’s Hospital Medical Center, Cincinnati, 45229 OH USA; 5 Divisions of Biomedical Informatics and Developmental Biology, Cincinnati Children’s Hospital Medical Center, Cincinnati, 45229 OH USA

## Abstract

Hepatoblastoma (HBL) is a pediatric liver cancer that affects children under the age of three. Reduction of tumor suppressor proteins (TSPs) is commonly seen in liver cancer. However, in our studies we find that aggressive, chemo-resistant HBLs exhibit an elevation of TSPs. HBL patients with a classic phenotype have reduced TSP levels, but patients with aggressive HBL express elevated TSPs that undergo posttranslational modifications, eliminating their tumor suppression activities. Here we identify unique aggressive liver cancer domains (ALCDs) that are activated in aggressive HBL by PARP1-mediated chromatin remodeling leading to elevation of modified TSPs and activation of additional cancer pathways: WNT signaling and β-catenin. Inhibition of PARP1 blocks activation of ALCDs and normalizes expression of corresponding genes, therefore reducing cell proliferation. Our studies reveal PARP1 activation as a mechanism for the development of aggressive HBL, further suggesting FDA-approved PARP1 inhibitors might be used for treatment of patients with aggressive HBL.

## Abstract (web summary)

Leila Valanejad et al. report increased expression of modified tumor suppressor proteins (TSPs) with loss of tumor suppressor activity in aggressive, chemotherapy-resistant hepatoblastoma. They find that TSP upregulation occurs via PARP1-mediated chromatin remodeling, leading to activation of multiple cancer-associated pathways.

## Introduction

Hepatoblastoma (HBL) is the most common type of malignant pediatric liver cancer, affecting children in their first 3 years of life^1,2^. While overall survival for children with HBL has improved over the years through cisplatin-based chemotherapy and subsequent resection, a substantial number of patients experience metastasis or are faced with aggressive tumors that are unresectable and do not respond favorably to chemotherapy^2–4^. Several recent studies reported that HBL is a genetically simple tumor with an average of 2.9 mutations per tumor predominately in β-catenin and *NFE2L2* genes^5–7^ and in the Wnt pathway^8^. These reports demonstrate that genomic mutations are only one part of the complex alterations observed in HBL. The quiescent liver expresses up to 20 tumor suppressor proteins (TSPs) that are involved in the protection of the liver from the development of cancer; however, the elimination of TSPs is a common trend seen in many types of liver cancer^9,10^. Ubiquitin-proteasome-mediated degradation of TSPs is one of the main pathways of elimination of tumor suppressor proteins. This pathway depends on the small subunit of the 26S proteasome Gankyrin (Gank) that triggers degradation of TSPs by direct interactions or through activation of proteins that degrade TSPs^11^. It has been previously reported that the farnesoid X receptor (FXR) represses Gank and that the reduction of FXR increases expression of Gank^12–14^. In the majority of patients with classic, chemo-sensitive hepatoblastoma, alterations of the FXR-Gank axis lead to a failure of hepatic stem cells to differentiate into hepatocytes^14^.The causal role of FXR and Gank in the development of liver cancer in adult patients and in animal models has been documented in many reports^11,12,15^. Particularly, FXR KO mice and double FXR/SHP KO mice develop spontaneous liver cancer at 17 and 12 months, respectively^12^. Liver-specific overexpression of Gank has been shown to facilitate the development of liver cancer under DEN/CCl_4_-mediated cancer^16^. Overexpression of Gank in livers of zebra fish has recently been shown to develop spontaneous intrahepatic cholangiocarcinoma and hepatocellular carcinoma^17^.

While the FXR/Gank axis appears to play a primary role in the development of liver cancer, this pathway does not always lead to the elimination of TSPs. Our new results show that many TSPs are elevated in aggressive HBL as oncogenic isoforms. Furthermore, the elevation of these oncogenic isoforms is mediated by activation of poly (ADP-ribose) polymerase, PARP1. PARP1 is a nuclear protein classically identified as an enzyme involved in the repair of double-stranded DNA breaks^18^. However, recent publications revealed that PARP1 is also a potent transcriptional regulator and has activities associated with oncogenic properties^19^. Transcriptional activities of PARP1 are associated with regulation of transcription factors, changes of the chromatin structure, and direct interactions with chromatin remodeling proteins^18–20^. Additionally, PARP1 interacts with complexes of RNA pol II^21^. Several studies showed that the transcriptional activities of PARP1 are involved in the promotion of cancer^18^. PARP1 occupies and activates promoters of key pluripotency genes, protecting these genes from epigenetic repression^22^. PARP1 also represses the activity of FXR by poly(ADP-ribosyl)ation associated with the removal of FXR from its binding sites^23^. It has been shown that PARP1 binds to the E2F1 protein and functions as a strong activator of *E2F1* gene expression^24^. Additionally, PARP1 modulates chromatin on the c-myc promoter leading to activation of the *c-myc* gene^25^. Another cancer-related activity of PARP1 is its recruitment of a SNF2 family member (known as “amplified in liver cancer 1” (ALC1) gene) to DNA^26^. In addition, PARP1 poly-(ADP-ribosyl)ates transcription factor Sp1 positively regulates cell cycle progression through downregulation of checkpoint proteins p21 and p27^27^.

In this paper, we present evidence for the critical role of PARP1 in aggressive chemo-resistant pediatric liver tumors. PARP1 is elevated in aggressive HBL form complexes with Ku80 and Ku70 and binds to the core 18 base pair sequence (18BPS) within a larger 250 bp aggressive liver cancer domain (ALCDs). This binding activates a number of genes that play a critical role in the development of liver cancer. The inhibition of PARP1 by specific drugs and siRNA inhibits formation of PARP1/Ku80/Ku70 complexes, silences multiple pathways of liver cancer, and results in inhibition of proliferation of cancer cells.

## Results

### TSPs lose tumor suppressor activities in aggressive HBL

**Fig. 1:**
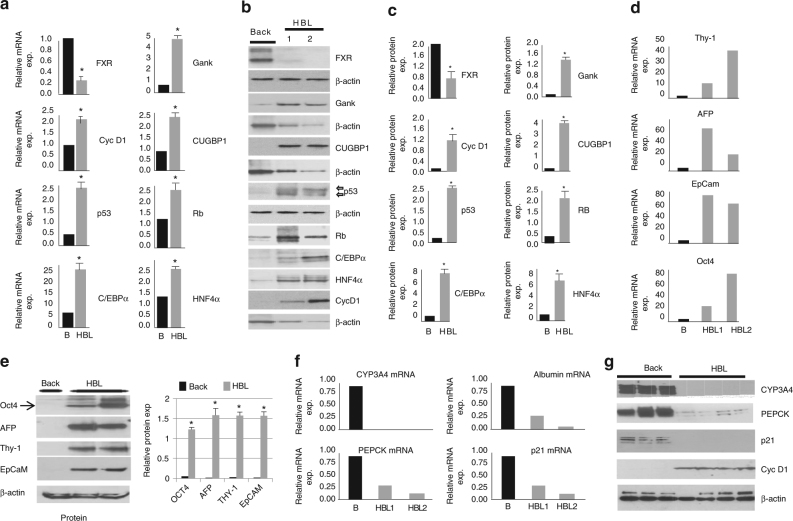
Investigations of tumor sections of an HBL patient with dramatically upregulated tumor suppressor proteins. Examination of a unique case in which liver was investigated immediately after surgery without freezing. The patient had two distinct HBL tumor sections which had a distinct morphology, but both were aggressive HBLs. We obtained background region and two tumor sections which we named HBL1 and HBL2. **a**, **b** QRT-PCR (**a**), western blotting (**b**), and quantitation (**c**) show elevation of mRNAs and tumor suppressor proteins correspondingly in these fresh chemo-resistant HBL. Arrows indicate appearance of two isoforms for p53. **c** Bar graphs show levels of proteins as ratios to β-actin control. **d** Markers of stem cells are elevated in HBL samples with high levels of TSP as compared to background tissue at mRNA and **e** protein levels. Right: bar graphs show levels of proteins as ratios to β-actin. **f**, **g** Targets of C/EBPα, HNF4α, and p53 are dramatically reduced at the mRNA (**f**) and protein (**g**) levels in HBL samples with high levels of these proteins. QRT-PCR and western blotting were performed correspondingly. Two additional HBL samples were added for western blotting. Error bars represent standard error of the mean (**a**, **c**, **e**)

Our group has recently shown that the elimination of tumor suppressor proteins (TSPs) and subsequent failure of stem cells to differentiate into hepatocytes is the cause of classic HBL^14^. In the previous study^14^, we performed RNA-Seq analysis (Supplementary Fig. 1), which revealed a complex transcriptome profile for hepatoblastoma including an increase in expression of stem cell markers, a dramatic decrease in expression of hepatocyte markers (CYP family and OCT family), and surprisingly, often includes increases in expression of TSPs found mainly in patients with aggressive liver cancer. Table 1 shows the selected information regarding these alterations. More details can be found in our paper^14^. These findings led us to examine the tumor suppressor proteins in aggressive pediatric liver cancer. Experimental work with frozen liver samples is often associated with possible degradation of proteins and RNAs. Therefore, in addition to frozen HBL samples, we collected fresh HBL and background samples immediately after surgical resection. These fresh samples (two separate large tumor nodules, called HBL1 and HBL2) were obtained from a patient that presented with aggressive HBL and did not respond to cisplatin-based chemotherapy. Figure 1 shows an example of examination of these fresh samples. Although there is activation of the FXR−Gank pathway, these samples express very high levels of TSPs proteins that one would assume should have been neutralized by Gank (Fig. 1a–c). Interestingly, we have also observed elevation of two isoforms of p53 with a difference of 2 kD (Fig. 1b). It has been previously shown that HNF4α and C/EBPα are involved in the differentiation of hepatic stem cells into mature hepatocytes^11,28^. However, our studies revealed very high levels of stem cell markers at both mRNA and protein levels (Fig. 1d, e), demonstrating that C/EBPα and HNF4α have lost their ability to promote differentiation of hepatic stem cells into hepatocytes.

**Table 1:** Summary of investigations of a large cohort of HBL samples by RNA-Seq, QRT-PCR, and western blotting

Genes	Up	Down	No change
*Tumor suppressor genes*			
Rb	9	19	13
p53	12	15	14
C/EBP*α*	18	8	15
HNF4*α*	16	9	16
CUGBP1	6	19	16
*Stem cell markers*			
4-Oct	40	0	1
AFP	40	0	1
Thy-1	40	0	1
EpCam	39	0	2
*Hepatocyte markers*			
CYP Family	0	41	0
OCT Family	0	41	0

Forty-one HBL samples were analyzed by RNA-Seq. The table presents selected groups of genes from the categories of TSPs, stem cell markers, and markers of hepatocytes. Six HBL samples have increased levels of five tumor suppressor proteins. The whole set of data for RNA-Seq can be found in our previous publication^14^

spr

To further examine the activities of C/EBPα, HNF4α, and p53, we examined expression of genes that are direct downstream targets of these proteins. CYP3A4 (target of C/EBPα and HNF4α), PEPCK and albumin (targets of C/EBPα), and p21 (target of p53) mRNA expression were examined in our samples that expressed high levels of C/EBPα, HNF4α, and p53. We found that these targets were dramatically reduced in the HBL samples with very high levels of C/EBPα, HNF4α, and p53 and elevated Cyclin D1 expression (Fig. 1f, g, respectively), demonstrating that these TSPs have lost their transcriptional and, perhaps, tumor suppressor activities. Interestingly, an increase of cyclin D1 indicates the increased proliferation in aggressive HBL. These observations led us to perform extensive analyses of the function and possible modifications of TSPs in these and in additional aggressive HBL samples.

### C/EBPα, HNF4α, CUGBP1, and RB are modified in aggressive HBL

In order to understand the mechanisms by which tumor suppressor proteins are neutralized in aggressive HBL, we first performed exome sequencing of aggressive HBL and examination of possible mutations within TSPs. While we detected mutations in *ARID1A*, *MDM4*, and *NRF2* genes, no mutations were found in coding regions of TSPs (Supplementary Fig. 2). Therefore, we suggested that posttranslational modifications of the TSPs might change their activities in patients with aggressive HBL. To test this hypothesis, we conducted extensive proteomic analysis of the activities of C/EBPα, HNF4α, p53, and RB.

**Fig. 2:**
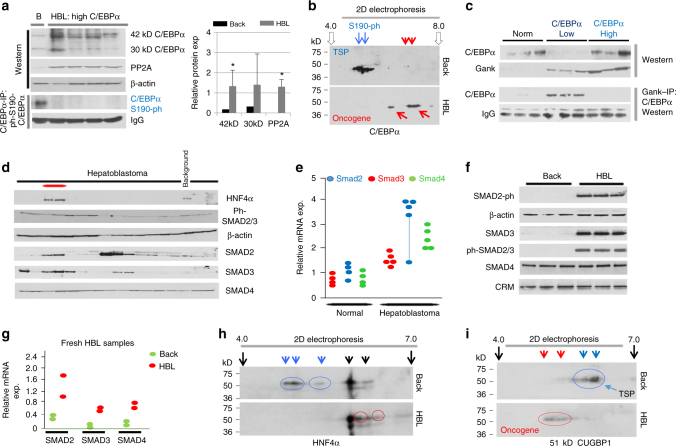
Tumor suppressor proteins C/EBPα and HNF4α are dephosphorylated in HBL samples with high levels of these proteins. **a** C/EBPα is dephosphorylated at Ser190 in HBL samples with high levels of C/EBPα. Western blotting was performed with protein extracts isolated from background and tumor sections of HBL samples from patients with aggressive phenotype. Antibodies to total C/EBPα and to PP2A were used. Bottom portion shows Co-IP of total C/EBPα and western with antibodies to ph-S190-C/EBPα. Bar graph (right) shows the level of proteins as ratios to β-actin. Data for four HBL samples are shown. **b** 2D-electrophoresis shows that ph-S190-C/EBPα is not detected in HBL samples. Nuclear extracts from background (back) and tumor sections (HBL) of the same patient were separated by 2D technique and probed with antibodies to C/EBPα. Red arrows show dephosphorylated (oncogenic) forms of C/EBPα, blue arrows show tumor suppressor isoforms of C/EBPα. **c** De-ph-C/EBPα does not interact with Gank. Upper image shows western blotting with Abs to C/EBPα and Gank; bottom image shows Gank-IP and western blotting with C/EBPα Abs. Three samples of each group were analyzed. **d** Western blotting of 16 frozen HBL samples with Abs to proteins of TGFβ-SMAD signaling. Red bar indicates samples with elevated expression HNF4α that have activation of downstream SMAD proteins (shown on the right). **e** Levels of mRNAs of SMAD family determined by QRT-PCR for frozen samples. **f** Western blotting with background and three HBL samples with antibodies to proteins of SMAD pathway. **g** Levels of mRNAs of SMAD family determined by QRT-PCR for fresh samples. **h**, **i** 2D examination of HNF4α and CUGBP1 in aggressive HBL samples. Blue arrows/circles indicate tumor suppressor isoforms of HNF4α and CUGBP1, red arrows/circles show oncogenic forms of HNF4α and CUGBP1. Error bars represent standard error of the mean (**a**)

It has been shown that Akt-PPA2-mediated dephosphorylation of mouse C/EBPα at Ser193 and human C/EBPα at Ser190 blocks tumor suppression activity of C/EBPα^29,30^. We examined if this pathway is involved in the neutralization of C/EBPα in four HBL samples with high levels of two isoforms of C/EBPα. Figure 2a shows that both isoforms of C/EBPα (42 kD and 30 kD) are increased compared to background HBL tissues. We examined the expression of PP2A (the enzyme which dephosphorylates human C/EBPα at Ser190^29^) and found that it is elevated in these HBL samples (Fig. 2a). We examined phosphorylation status of Ser190 using two approaches: C/EBPα IP-western with antibodies to ph-S190 isoform of C/EBPα and 2D gel electrophoresis. We found that phosphorylated forms of Ser190-C/EBPα are not detected in these HBL samples (Fig. 2a). 2D gel electrophoresis confirmed that the phosphorylated forms of Ser190-C/EBPα that are present in the background tissue (Fig. 2b, blue arrows) are not detectable in HBL tissues.

We next examined if dephosphorylation of C/EBPα at Ser190 in HBL samples creates its resistance to degradation by Gank. Since Gank does not recognize mouse C/EBPα dephosphorylated at Ser193^31^, we performed studies to determine if this phenomenon was recapitulated in pediatric liver cancer. We performed Co-IP studies using control samples from normal livers, HBL samples with low levels of C/EBPα, and HBL samples with high levels of C/EBPα (Fig. 2c). To normalize levels of C/EBPα in IP reactions, we used fivefold higher amounts of HBL samples with low levels of C/EBPα. As seen in Fig. 2c, Gank interacts with C/EBPα in extracts with low levels of C/EBPα, but not with C/EBPα in HBL samples with dephosphorylated C/EBPα. Thus, these studies revealed that dephosphorylation of C/EBPα at Ser190 also makes this tumor suppressor protein resistant to Gank-mediated degradation, resulting in elevated levels of C/EBPα in aggressive, chemo-resistant HBL.

Although HNF4α is a strong TSP, it has been shown that its activity could be neutralized by dephosphorylation through the TGFβ–SMAD pathway^32^. We tested if this pathway is activated in HBL samples with high levels of HNF4α. In addition to several HBL samples, we tested two HBL samples with high levels of HNF4α (labeled by a red line) to examine activation of TGFβ signaling as compared to background tissue (Fig. 2d). Likewise, targets of the TGFβ–SMAD pathway were also active at the mRNA level (Fig. 2e). Additional analysis with fresh chemo-resistant HBL samples found that TGFβ signaling is activated at the protein (Fig. 2f) and mRNA (Fig. 2g) levels. 2D examination of HNF4α in fresh HBL samples showed that, unlike in HBL sections, background samples contain HNF4α isoforms in acetic regions (blue arrows) (Fig. 2h). On the contrary, HBL samples contained new isoforms (red circles) located in alkaline regions of the 2D gel, suggesting that posttranslational modifications that occur in aggressive HBL result in the neutralization of the tumor suppressor activity of HNF4α.

We have recently found that RNA-binding protein CUGBP1 is a tumor suppressor protein when it is dephosphorylated at Ser302, but it is an oncogene when it is phosphorylated at this residue^13^. Therefore, we examined CUGBP1 isoforms in samples with aggressive HBL by 2D Gel electrophoresis and found that background regions contain mainly un-ph-tumor suppressor isoforms (shown blue arrows in Fig. 2i); while tumor sections do not have these isoforms, but contain oncogenic isoforms (shown by red arrows). These studies demonstrated that posttranslational modifications converted CUGBP1 in oncogenic form in aggressive HBL.

**Fig. 3:**
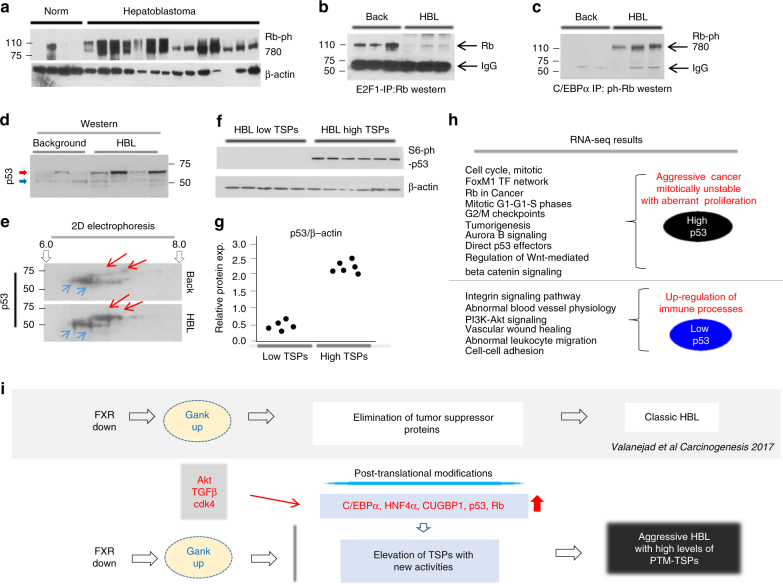
Rb and p53 are posttranslationally modified in HBL samples with high levels of these proteins. **a** Western blotting was performed with Abs to S780-phosphorylated Rb using liver nuclear extracts of four normal livers and 14 HBL samples. The filter was re-probed with Abs to β-actin. **b** Immunoprecipitation of E2F1 and western blotting with Abs to Rb was performed with protein extracts isolated from three background and three tumor sections of HBL samples from patients with aggressive phenotype. IgG image on bottom. **c** Immunoprecipitation of C/EBPα and western blotting with Abs to Rb. IgG: signal of IgG that are detected in IPs. **d** Western blotting with protein extracts isolated from three background and four HBL sections with Abs to p53. Red arrow indicates high molecular weight isoform of p53. **e** 2D gel electrophoresis of p53 using nuclear extracts isolated from fresh background and HBL. Blue arrows show p53 isoforms detected in both background and tumor sections, red arrows show isoform that are detected only in tumor sections (HBL). **f** Western blotting of nuclear extracts isolated from background and HBL sections with Abs to Ser6-ph p53. The film was re-probed with Abs to β-actin. **g** Dot plots show levels of p53 as ratios of p53 to β-actin. **h** Examination of signaling pathways in HBL with high and low levels of p53 by RNA-Seq analysis. HBL samples with high level of p53 have much higher elevation of pathways typically activated in aggressive liver cancer. **i** Summary of studies of a large Biobank of HBL samples. The upper part summarizes our recently published findings^14^. These studies and current manuscript identified two types of HBL: classic HBL and aggressive HBL. Aggressive HBL expresses high levels of TSPs which underwent posttranslational modifications. PTM posttranslational modification

It has been shown that neutralization of the tumor suppressor activity of RB is usually mediated by cdk4-dependent phosphorylation at Ser780^33^. As shown in Fig. 3a, posttranslational modification status of RB in HBL samples with an aggressive phenotype was tested and has shown that RB is phosphorylated at Ser780, supporting neutralization of tumor suppressor activity seen in aggressive HBL. Consistent with reports showing that phosphorylation of RB at Ser780 disrupts E2F1–RB complexes repressors^33^, Co-IP results from Fig. 3b indicate that E2F1–Rb complexes in HBL samples with high levels of RB are dramatically reduced. Since C/EBPα interacts with RB, we asked if dephosphorylated C/EBPα and phosphorylated RB interact with each other in HBL samples with high levels of both proteins. In fact, Co-IP analysis showed that these proteins form a new stable complex, ph-S780-Rb-de-ph-C/EBPα, in samples that express high levels of both modified proteins (Fig. 3c).

### Modified p53 correlates with aggressive HBL phenotype

p53 is a well-described TSP^34^ and the identification of HBL samples with high levels of this protein was surprising. To investigate consequences of p53 elevation, we performed a set of experiments to examine the status of elevated p53 and transcriptome profiling in an aggressive phenotype of HBL. As shown in Fig. 1b, two isoforms of p53 are elevated in fresh HBL samples; therefore, we examined if additional HBL samples from our cohort might have a similar pattern. Western blotting of additional samples revealed that in fact multiple aggressive chemo-resistant HBL samples expressed two isoforms of p53 (Fig. 3d). We further examined posttranslational modifications of p53 in our cohort of samples by 2D gel electrophoresis and found that in background tissue of HBL samples, the upper isoform of p53 is less acetic than the bottom isoform (Fig. 3e). We next investigated possible posttranslational modifications of p53 that result in reduction or neutralization of its tumor suppressor activity. It is known that when p53 is phosphorylated at Ser6, it loses its ability to suppressor tumor activity^35^. We examined the protein expression of Ser6 phosphorylated p53 in HBL samples that expressed high levels of p53 and confirmed that p53 was phosphorylated, leading to the outcome of inhibition of tumor suppressor function (Fig. 3f, quantified Fig. 3g). Lastly, we analyzed signaling pathways in HBL samples with low levels of p53 and high levels of p53 using RNA-Seq. This comparison showed that signaling pathways with the highest levels of gene activation in HBL samples expressing increased p53 are typical for aggressive liver cancer with mitotic instability; while the pathways with highest gene activation in HBL samples expressing decreased p53 are involved in activation of immune processes (Fig. 3h). Figure 3i outlines the identification of two types of HBL based on tumor suppressor protein expression and functions. It is important to note that previous reports revealed activation of pathways (such as Akt, TGFβ, and cdk4) in liver cancer that might cause the observed posttranslational modifications of TSPs^14,29,32^.

### Identification of ALCDs

**Fig. 4:**
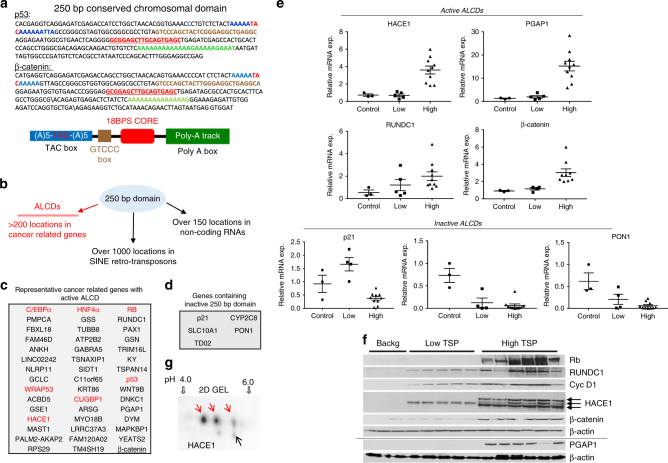
Identification of aggressive liver cancer domains (ALCDs) in the genomic regions of five tumor suppressor genes and in many cancer-related genes. **a** Top: A typical representation of the 250 bp domain located within p53 and β-catenin genes. 100% homological core 18BP sequence is shown in red. Additional highly homological regions are shown by blue, brown, and green colors. Bottom: Schematic presentation of 250 bp domains structure. **b** Proposed distribution of 250 base pair domains within human genome. The domains located in TSPs and in more than 200 cancer-related genes are called aggressive liver cancer domains (ALCDs). **c** List of cancer-related genes containing ALCDs. Red color shows tumor suppressor proteins. **d** List of downregulated genes identified by RNA-Seq analysis that contain an inactive 250 bp domain. **e** Active ALCDs: QRT-PCR confirmation of activation of genes containing ALCDs in HBL samples with high levels of TSPs. Inactive ALCDs: QRT-PCR confirmation of low expression of genes containing inactive 250 bp domain in HBL samples with high levels of TSPs. **f** Western blotting revealed that additional cancer-related genes are also elevated to higher degree in patients with aggressive HBL that contain high levels of TSPs. HACE1 protein is expressed in aggressive HBL samples as three isoforms. Western blotting for PGAP1 and corresponding loading control are performed with a separate gel. **g** 2D gel electrophoresis of HACE1 using nuclear extracts isolated from a fresh tumor liver sample. Red arrows indicate isoforms that have modifications. Error bars represent standard error of the mean (**e**)

We next investigated mechanisms which support elevation of the posttranslationally modified TSPs in aggressive pediatric liver cancer. We inspected the genomic regions of C/EBPα, HNF4α, RB, p53, and CUGBP1 and discovered a conserved (100% homological) 18 base pair sequence (18BPS) (Fig. 4a, red). Examination of surrounding sequences in each *TSP* gene revealed that the 18BP sequences are located within a larger 250 base pair domains that are 70–80% homologous (Fig. 4a top, ALCDs of p53 and β-catenin shown as examples). The 250 base pair domain consists of the core 18BPS (shown in red) with a TAC-box (shown in blue), a GTCCC-box (shown in brown) and a poly-A box (shown in green) (Fig. 4a, bottom), with entire sequence sharing homology to the AluY family of short interspersed nuclear element (SINE) retro-transposons. A portion of these genes is involved in various cancer-related pathways (Fig. 4b and Supplementary Figs. 2–12). Among these pathways, we have identified *β-catenin* gene which is a well-characterized marker of aggressive hepatoblastoma and strong initiator of liver cancer. Note that further studies revealed that many of these domains are selectively activated in cancer-related genes, and inactive in other locations of the genome (see below). We henceforth refer to these active domains as aggressive liver cancer domains (ALCDs) and to silenced domains as 250 bp inactive chromosomal domains (Fig. 4c, d). To further characterize the ALCDs, we examined genes that were identified by RNA-Seq of HBL samples as having reduced expression (Supplementary Fig. 12). Among the top 42 downregulated genes identified by RNA-Seq, we identified five genes that contain the core 18BPS and subsequent 250 bp domains (Fig. 4d and Supplementary Fig. 12). Further studies revealed that activators of ALCDs are not bound to these domains in these five genes (see below).

To determine if the newly identified genes that housed ALCDs were elevated in our HBL sample cohort, we examined the expression of RUNDC1, HACE1, β-catenin, and PGAP1. Figure 4e (active ALCDs) shows that these genes are all expressed at higher levels in HBL samples that also express high levels of TSPs, compared to HBL samples that express low levels of TSPs. Supplementary Fig. 13A shows activation of an additional ALCD-containing gene MYO18B. However, p21, PON1, and CYP2C8 that house the inactive 250 bp domain are not activated in HBL and therefore serve as negative controls in our analysis (Fig. 4e, inactive ALCDs and Supplementary Fig. 13B). The activation of ALCD-containing genes was also confirmed at the protein level by western blot analysis (Fig. 4f). It is important to note that one of the identified genes with an ALCD, HACE1, codes for a tumor suppressor protein^36^. Interestingly, we determined that HACE1 is observed as three isoforms in samples that express high levels of TSPs. This suggests possible posttranslational modifications that could alter the function of this protein in aggressive HBL, in this case, possibly converting HACE1 into an oncogene. To examine this possibility, we performed 2D gel electrophoresis of HBL samples with high levels of TSPs. These studies revealed that three isoforms of HACE1 have different charges, indicating that they have different posttranslational modifications (Fig. 4g). Therefore, in addition to five TSPs, these studies identified the elevation of posttranslationally modified HACE1 in aggressive pediatric liver cancer.

### PARP1, Ku80, and Ku70 bind to the core 18BPS of ALCDs

**Fig. 5:**
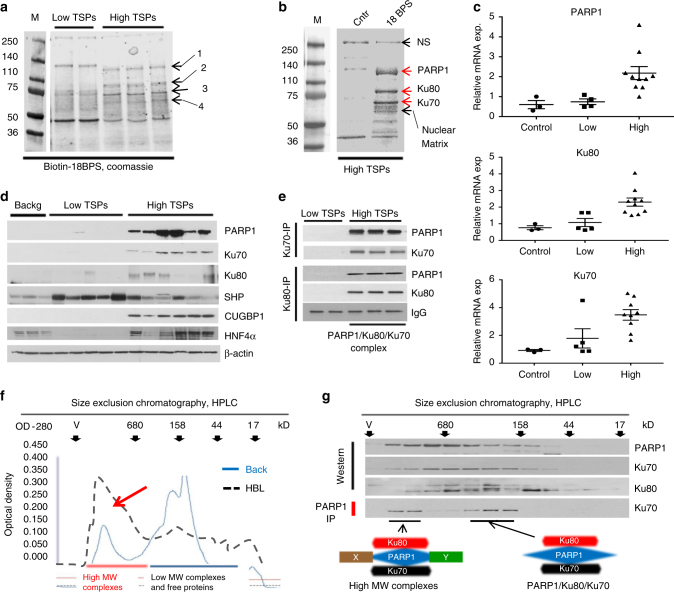
PARP1, Ku80, and Ku70 are dramatically elevated in patients with aggressive HBL and bind to the core sequence of ALCDs. **a** The core 18BPS was linked to biotin beads and was incubated with nuclear extracts isolated from HBL with low and high levels of TSPs. Coomassie staining identified four proteins (shown on the right) that specifically interacted with the 18BPS in nuclear extracts from aggressive HBL. **b** Large-scale isolation and mass spec analysis of the interacting proteins. The proteins which are specifically interacting with 18BPS are PARP1, Ku80, Ku70, and several additional proteins, some of them belong to nuclear matrix (Supplementary Fig. 14). **c**, **d** Expression of PARP1, Ku80, and Ku70 is dramatically increased in patients with aggressive HBL. QRT-PCR (**c**) and western blotting (**d**) were performed with mRNAs and proteins isolated from HBL samples with low and high TSPs levels. SHP is a small heterodimer partner, which was detected by re-probe of the PARP1 membrane and which serves as a good control for protein loading. **e** PARP1, Ku80, and Ku70 form a complex in livers of patients with aggressive HBL. Ku70 and Ku80 were precipitated and PARP1 and Ku70 or Ku80 were determined in these IPs. IgG: heavy chains of IgG. **f** Fractionation of nuclear proteins from background (blue) and HBL (black) sections of aggressive HBLs by HPLC-based size exclusion chromatography (SEC). Red arrow shows elevation of optical density in the area of high MW protein–protein complexes. **g** Top: Examination of PARP1/Ku80/Ku70 complexes in SEC fractions. Western blotting shows the amount of proteins in each fraction. Bottom: PARP1-IP shows immunoprecipitation of PARP1 and analysis of Ku70 in these IPs. Images below show hypothetical compositions of PARP1 complexes. Error bars represent standard error of the mean (**c**)

To understand the role of ALCDs in cancer, we synthesized biotin-labeled oligonucleotides that correspond to the core 18BPS located within ALCDs. After incubation with HBL samples that express either low or high levels of TSPs, proteins bound to our 18BPS were separated by SDS electrophoresis. Figure 5a shows that four proteins specifically interact with the 18BPS oligonucleotide in samples with high TSPs expression. Mass spectrometry analysis revealed PARP1, Ku80, and Ku70 specifically interact with the 18BPS in samples that contained high levels of TSPs as compared to control DNA (Fig. 5b). Several additional proteins of the nuclear matrix that show weak interaction with 18BPS were also identified (Supplementary Fig. 14). Figure 5c, d indicates that PARP1, Ku80, and Ku70 were elevated to a higher degree in aggressive HBL samples that expressed high levels of TSPs than in HBL samples that expressed low levels of TSPs. Furthermore, co-immunoprecipitation assays revealed that PARP1, Ku80, and Ku70 form a complex in aggressive HBL, which is not seen in HBL samples that express low levels of TSPs (Fig. 5e). Taken together, we conclude that PARP1, Ku80, and Ku70 bind as a complex to the core 18BPS of ALCDs and that these proteins are dramatically elevated in patients with aggressive HBL.

### PARP1 activates the ALCDs in aggressive HBL

We next investigated the composition and activity of the PARP1/Ku80/Ku70 complex. For these studies, we used fresh HBL and corresponding background regions from patients with aggressive, chemo-resistant HBL. Nuclear extracts were subjected to HPLC-based size exclusion chromatography (SEC). Surprisingly, analysis of optical density of SEC shows a significant enrichment in regions of high MW complexes (Fig. 5f, *t*-test, *p* < 0.05). We next asked if the PARP1/Ku80/Ku70 complex was present within the high molecular weight fractionated sections. Indeed, western blotting showed that, in the tumor tissue, PARP1, Ku80, and Ku70 are located in high molecular weight regions of SEC. Co-IP studies showed that these proteins form two distinct complexes: a complex in high MW region (between 1 mln and 680 kD) and another complex between 168 and 680 kD (Fig. 5g). This analysis suggests that high MW PARP1 complexes contain additional components; while lower MW complexes presumably represent these three proteins.

**Fig. 6:**
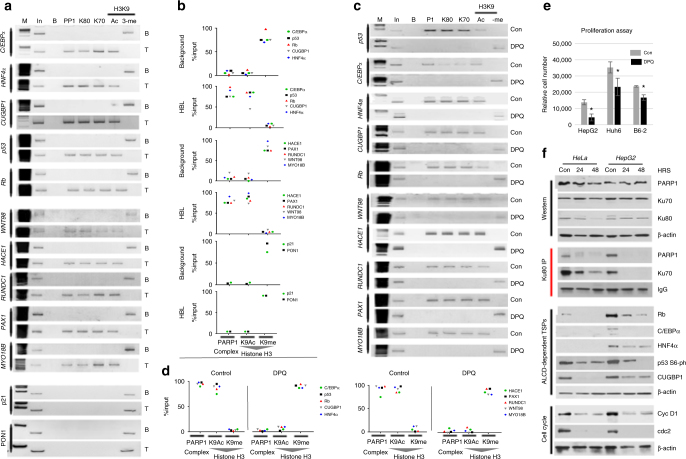
ALCDs are activated in aggressive HBL samples and in hepatoblastoma cancer cells by PARP1/Ku80/Ku70 complexes. **a** ChIP analysis of the ten representative ALCDs and two negative controls, which contain the inactive 250 bp domain (shown on the left and right) in background and HBL sections of the livers. M marker, In input, B beads, PP1 PARP1, H3K9-Ac histone H3 acetylated at K9, 3-me histone H3 trimethylated at K9. **b** Quantitative presentation of ChIP analysis. Amounts of PARP1/Ku80/Ku70 complexes were calculated as average of PARP1, Ku80, and Ku70 signals and then as percentage of this average signal to input. **c** ChIP analysis of ten representative ALCDs in HepG2 cells with and without treatment with 100 µM DPQ. **d** Quantitative presentations of ChIP analysis. Calculations were performed as described above. **e** Cell proliferation assay in HepG2, Huh6, and B6-2 cells treated with 100 µM DPQ for 72 h. Representative images highlighting changes in cell proliferation after DPQ treatment are shown in Supplementary Fig. 18a. **f** Inhibition of PARP1 by DPQ reduces PARP1/Ku80/Ku70 complexes in HeLa and in HepG2 cells and reduces expression of TSPs and cell cycle proteins. Cells were treated with DPQ for 24 and 48 h and western blotting and Co-IPs were performed as described above. Error bars represent standard error of the mean (**e**)

Based on these studies, we hypothesized that PARP1/Ku80/Ku70 complexes activate chromosomal regions of ALCDs. To test this hypothesis, we asked if these complexes occupy and regulate chromatin structure around ALCDs of five TSPs and five other cancer-related genes. As negative controls, we have included p21 and PON1, which house the inactive 250 bp domain. Figure 6a demonstrates that in all ALCDs examined, PARP1, Ku80, and Ku70 bind directly to the ALCDs in HBL tumor sections and not in background tissues. However, PARP1, Ku80, and Ku70 are not bound to the 250 bp domain of p21 and PON1 in background and HBLs sections of the liver. Figure 6b presents quantitative ChIP results for these genes (additional genes shown in Supplementary Fig. 15). It has been recently shown that PARP1 complexes activate transcription of genes by acetylation of histone H3^16^. ChIP analysis reveals that histone H3 is acetylated at K9 in all aggressive HBL samples for all genes examined, signifying activation of ALCDs in HBL samples and not in background tissue where histone H3 is instead trimethylated at K9. The 250 bp domains of negative controls contain trimethylated H3K9 showing that these domains are silenced (Fig. 6a, b). Taken together, our results indicate that PARP1/Ku80/Ku70 complexes activate cancer-specific genes via binding to ALCDs.

### Inhibition of PARP1 by DPQ silences ALCDs

PARP1 plays a role in the regulation of multiple transcriptional pathways that may be involved in cancer formation including promotion of cell proliferation. These pathways include decrease in FXR expression and activation of c-myc^18^. It is interesting that PARP1 inhibitors have long been used for cancer therapy and are currently in use in multiple clinical trials^37^. Pharmacological inhibition of PARP1 by DPQ has been shown to reduce tumor growth and progression in mouse models of liver cancer^38^. To understand if inhibition of PARP1 modifies its interactions with ALCDs within C/EBPα, p53, HNF4α, CUGBP1, and RB genes, as well as with ALCDs of the additional cancer-related genes, we performed ChIP analysis on HepG2 cells treated with 100 µM DPQ. Figure 6c, d demonstrates that inhibition of PARP1 by DPQ blocks the interactions of PARP1, Ku80, and Ku70 with ALCDs in all genes tested. Furthermore, the inhibition of PARP1 leads to methylation of histone H3 at K9 for all genes, showing repression of ALCDs in treated HepG2. Additional information for quantitative ChIP is shown in Supplementary Figs. 16 and 17. These results suggest that in background human tissue as well as in HepG2 cells treated with a PARP1 inhibitor, the ALCDs are not active. PARP1 regulates transcription of genes and cell proliferation through several pathways^19,24,26,27^. Therefore, we next examined if DPQ-mediated inhibition of PARP1/Ku80/Ku70 complexes and subsequent repression of the ALCDs in hepatoblastoma cells might inhibit cell proliferation. Figure 6e reveals that the inhibition of PARP1 by treatment with DPQ decreases cell proliferation. Representative images of cells with decrease in cell proliferation are shown in Supplementary Fig. 18a.

**Fig. 7:**
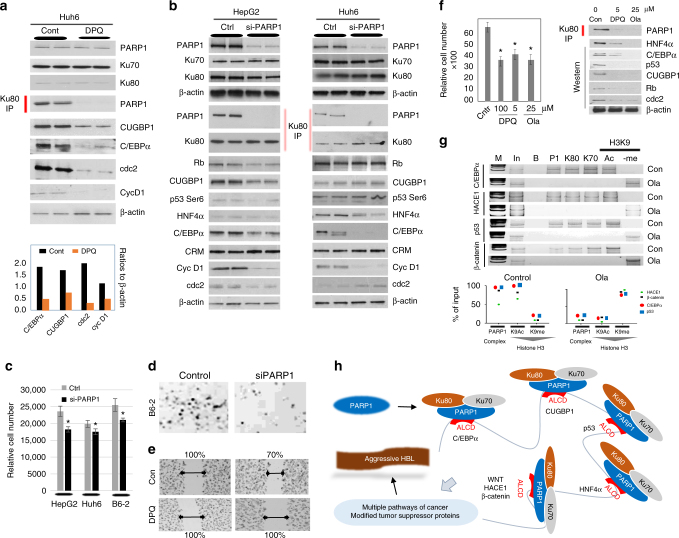
Inhibition of PARP1 by FDA-approved inhibitor DPQ and by si-PARP1 siRNA eliminates PARP1 complexes leading to silence of ALCDs and to inhibition of cell proliferation. **a** Western blotting of proteins isolated from Huh6 cells treated with 100 µM DPQ for 48 h. Ku80-IP: Ku80 was immunoprecipitated and PARP1 was examined in these IPs. Bar graphs below show levels of proteins as ratios to β-actin. **b** HepG2 and Huh6 cells were transfected with siRNA-targeting PARP1. Expression of proteins shown on the left and right was analyzed by western blotting. Ku80-IP: western shows the disruption of PARP1/Ku80/Ku70 complexes by inhibition of PARP1. **c** Cell proliferation assay of HepG2, Huh6, and B6-2 cells transfected with si-PARP1. The assay was performed 48 h after transfection of siRNA. **d** Representative images highlighting changes in cell proliferation with si-PARP1 transfection in B6-2 cells. **e** Scratch assay of proliferation of HepG2 cells untreated (con) and treated with DPQ. Percentage shows % of not-closed scratches (inhibition of proliferation) at 48 h after scratch. **f** Cell proliferation assay shows inhibition of proliferation of HepG2 cells by low concentrations of DPQ and olaparib (Ola). Right image shows Co-IP of PARP1/Ku80/70 complexes and western blotting of downstream targets of ALCDs. **g** ChIP assay of the ALCD regions of within C/EBPα, HACE1, p53, and β-catenin genes in control and olaparib-treated HepG2 cells. Experiment was performed as described in legend to Fig. 6. Bottom images show quantitation of ChIP for ALCDs in these genes. **h** A diagram showing hypothesis which is based on the results of these studies. Aggressive liver cancer is associated with elevation of PARP1, which forms complexes with Ku80 and Ku70 and subsequent chromatin remodeling around ALCDs that leads to a dramatic activation of multiple pathways of liver cancer. An important part of this hypothesis is that certain TSPs are also activated by PARP1-ALCDs axis, but they are posttranslationally modified and are converted into proteins with potential oncogenic activities. Oncogenic activities of posttranslationally modified C/EBPα are shown in our recent publication^39^. Error bars represent standard error of the mean (**c**, **f**)

Previous studies have shown that the inhibition of PARP1 in HepG2 cells transfected in nude mice prevented liver cancer^38^. To elucidate if PARP1 inhibition regulates proliferation through ALCD-based mechanisms, we examined protein levels of ALCD-driven tumor suppressor proteins and cell cycle proteins under DPQ treatment in HeLa and HepG2 cells. The cells were treated with 100 μM DPQ for 24 and 48 h and proteins were analyzed by western blotting analysis and by Co-IP. Figure 6f demonstrates that while expression of the individual proteins PARP1, Ku80, and Ku70 is not altered, PAPR1/Ku70/Ku80 complexes are disrupted, perhaps by DPQ-mediated change of the PARP1 configuration. Levels of tumor suppressor proteins are substantially reduced with DQP treatment in both HeLa and HepG2 cells (Fig. 6f). Interestingly, HeLa cells do not express detectable levels of C/EBPα and HNF4α; however, levels of TSPs such as p53, Rb, and CUGBP1 are reduced by inhibition of PARP1. Examination of cell cycle proteins cyclin D1 and CDC2 showed that proliferation of HeLa and HepG2 cells is also inhibited. To confirm our results in HepG2 cells, we further examined the effects of PARP1 inhibition by DPQ treatment on the PARP1/Ku80/Ku70 complexes and TSPs in another hepatoblastoma cell line, Huh6. Figure 7a shows that the inhibition of PARP1 in Huh6 also disrupts the PARP1/Ku80/Ku70 complexes, reduces levels of C/EBPα and CUGBP1 and reduces proliferation as is shown for CDK2 and cyclin D1. These results confirmed the changes that were seen in HeLa and HepG2 cells.

Although the inhibition of PARP1 by DPQ is specific, it is difficult to rule out additional unknown effects of this drug. Therefore, we performed a set of experiments with a very specific siRNA-mediated inhibition of PARP1 using HepG2 and Huh6 cell lines. As seen in Fig. 7b, si-PARP1 inhibits PARP1 expression (more than 90% inhibition) leading to complete elimination of PARP1/Ku80/Ku70 complexes. In agreement with DPQ data, we found a substantial decrease in tumor suppressor proteins and a decrease in cell cycle protein expression. We next examined proliferation of three hepatoblastoma-derived cell lines, HepG2, Huh6, and B6-2, in which PARP1 was inhibited by si-PARP1. Cell proliferation assay (Fig. 7c, d and Supplementary Fig. 18b) and scratch assay (Fig. 7e) show that, similar to results with DPQ treatment, inhibition of PARP1 by siRNA results in a decrease of proliferation of these cells. Quantification of siRNA results shown in Fig. 7b for HepG2 is shown in Supplementary Fig. 19.

Our previous experiments were performed with relatively high concentrations of PARP1 inhibitor DPQ. To further examine if the inhibition of PARP1 might be potentially considered as a pharmaceutical tool for treatment of patients, we have examined if lower doses of DPQ and lower doses of another inhibitor of PARP1, olaparib, might inhibit proliferation of hepatoblastoma cells HepG2 through the ALCD–TSP pathway. Figure 7f shows that lower concentrations of DPQ (5 µM) and olaparib (25 µM) inhibit proliferation of HepG2 cells. Examination of PARP1/Ku80/70 complexes and TSPs revealed that treatments of HepG2 cells with 5 µM DPQ and 20 µM olaparib substantially reduced PARP1 complexes and levels of all examined TSPs and cdc2 (marker of mitosis, Fig. 7f, right image). ChIP assay showed that PARP1/Ku80/Ku70 complexes are removed from ALCDs by these inhibitors and the chromatin regions are silenced after this removal (Fig. 7g). Thus, these studies demonstrated that low doses of DPQ and olaparib effectively inhibit proliferation of hepatoblastoma cells via silencing ALCDs. Taken together, the data demonstrate that the inhibition of PARP1 by inhibitors and by specific siRNAs blocks the interaction of the PARP1/Ku70/Ku80 complex with genes containing ALCDs, resulting in a repression of ALCD and subsequent inhibition of proliferation of cancer cells. Figure 7h summarizes studies in this paper and shows the pathways by which elevation of PARP1 leads to the development of aggressive HBL.

## Discussion

In this paper, we present several findings toward the advancement of the field of pediatric liver cancer research. These new findings include elevation of posttranslationally modified and inactive TSPs; discovery of ALCDs that drive aggressive liver cancer with PARP1 as the main activator; and evidence toward the use of PARP1 inhibitors to treat aggressive pediatric liver cancer. We have previously reported that mild, chemo-sensitive HBL is triggered by Gank-mediated elimination of TSPs and failure of haptic stem cells to differentiate into hepatocytes^14^. In the course of our studies of HBL, we unexpectedly found that these TSPs and a new tumor suppressor protein, CUGBP1^13^, were dramatically elevated in aggressive and chemo-resistant HBL samples. In this regard, we have recently published that the posttranslationally modified C/EBPα possesses oncogenic activities and converts hepatocytes into cancer stem-like cells^39^. Thus, we identified two types of HBL, which are characterized by different expression and activities of TSPs and by different cell origins (Fig. 3i).

The second major discovery of our work is the identification of multiple 250 bp human chromosomal domains, a subset of which comprises aggressive liver cancer domains, ALCDs. Sequences resembling these domains can be found in many locations of the human genome. Based on sequence homology, most are likely derived from the AluY family of short interspersed nuclear element (SINE) retro-transposons. Although transposable elements are estimated to represent up to half of the human genome^40^, many human regulatory regions are thought to be transposon-derived^41^, with a growing number of examples of transposable elements capable of driving tissue-specific gene expression patterns^42–46^. Given the large number of instances of the 18BPS (and associated ALCDs) within the human genome, we focused our studies on ALCDs that are located proximal to genes involved in multiple pathways of liver cancer. We found that these ALCDs are under strong control of PARP1 and Ku80/Ku70 complexes. It is important to note that a strong activator of aggressive liver cancer, β-catenin, is also under control of PARP1-ALCD axis and is dramatically elevated in examined aggressive HBL (Fig. 4). It would be interesting to test if Yap/Hippo pathway might be also controlled by PARP1-ALCD axis. Importantly, we also identified sequences bearing resemblance to the ALCDs (which we term inactive 250 bp domains) that are not regulated by PARP1, Ku80, and Ku70. We searched for possible structural features of active ALCDs and inactive 250 bp domains; however, no significant differences were observed. Therefore, it is not clear at this stage how PARP1 is directed to active ALCDs, but not to inactive domains. Future studies are required to determine the precise mechanisms that discriminate between active and inactive domains.

The third and critical aspect of our work is in the translational nature of our findings. We have found that the PARP1/Ku70/Ku80 complex is elevated in aggressive HBL and directly binds to the newly identified ALCDs. Furthermore, inhibition of PARP1 by low doses of two inhibitors, DPQ and olaparib, blocks activation of TSPs and cancer-related genes in hepatoblastoma cell lines, HepG2, Huh6, and B6-2, which facilitates the development of clinical trials for patients with aggressive HBL. It has been shown that PARP1 has been considered as a putative initiator of cancer and that inhibition of PARP1 in HepG2 cells transfected into nude mice prevents liver cancer^38^. Additionally, PARP1 inhibitors have been shown to mitigate the effects of liver inflammation, fibrosis, and alcoholic and non-alcoholic steatohepatitis, which are all critical steps toward the progression of liver cancer^47,48^. PARP1 inhibitors are also being used in more than 100 clinical trials for cancer therapy and for clinical implications such as stroke and cardiac infarction^18^. Currently, there are two clinical trials (Phase I: NCT00526617 and Phase II: NCT01205828) in which the PARP1 inhibitor ABT-888 is used in combination with a DNA alkylating agent, temozolomide, for the treatment of hepatocellular carcinoma^37^. It is also important to note that our work with inhibitors of PARP1 does not exclude that other members of the PARP family (such as PARP2) might be also inhibited and contribute to inhibition of cell proliferation. The identification of a PARP1-dependent ALCDs in multiple pathways of liver cancer provides a strong rationale for the utilization of PARP1 inhibitors to treat aggressive liver cancer.

## Methods

### Antibodies

Antibodies to C/EBPα (14AA), Cyclin D1 (H-295), CUGBP1 (3B1), FXR (H-130), HNF4α (sc-6556), CYP3A4 (HL3), PEPCK (sc-32879), Thy-1 (sc-9163), p53 (sc-6243), cdc2 (sc-53) AFP (sc-8399), PCNA (sc-7907), RB (sc-50), FOXM1 (c-20), and SOCS1 (H-93) were purchased from Santa Cruz Biotechnology (Santa Cruz, CA). Oct4 (Pa1-16943) and EpCam (ab32392) were purchased from ThermoFisher (Fremont, CA). Gankyrin (12985S) was purchased from Cell Signaling (Danvers, MA), monoclonal anti-β-actin antibody was purchased from Sigma (St. Louis, MO). The majority of these antibodies were previously used by many investigators including our group. The results are published in previous papers^12,31^.

### Tissue culture

HepG2 cells were purchased from ATCC (HepG2 ATCC HB-0865). Cells were authenticated by ATCC prior to sending. B6-2 cells were generated by Dr. Bissig and were characterized elsewhere^49^. Scratch assay was performed with HepG2 cells as described elsewhere^50^.

### Normal, background, and HBL pediatric liver samples

Six normal, seven background, and 51 HBL liver samples were obtained from the Cincinnati Children’s Hospital Medical Center BioBank repository in collaboration with the Division of Pathology. Among these samples, six HBL samples were from patients with aggressive HBL. Depending on amounts of liver samples, four–six aggressive HBL were examined for each target. Normal tissues were used as the controls in QRT-PCR and western blotting assays. Background regions (non-tumor sections from the same livers) were also used in some experiments. These background regions were preliminary checked for the expression of hepatocyte and stem cell markers. Background sections with no stem cell markers and with high levels of markers of hepatocytes were used for further studies. We used background sections since it is important to show differences between tumor and non-tumor sections of the same patients. Samples we obtained from patients between the ages of 0.01 months and 6 years of age over the course of 10 years. Additionally, four background and five HBL fresh liver tissue samples were collected by the Division of Pediatric Surgery at CCHMC from four patients at the time of surgical resection. Informed consent was obtained by the patients and families prior to initiation of studies. These protocols were reviewed and approved by the Institutional Review Board at CCHMC (IRB protocol #2015-8826).

### ChIP analysis

Chromatin immunoprecipitation was performed as described in our previous publications^12,31,51^. Table of primers used located in Supplementary Fig. 20.

### Examination of proteins

Protein extracts were isolated from human livers as previously described^12,31^. Proteins (50–100 µg) were loaded on to 4–20% gradient gels (BioRad) and transferred to nitrocellulose membranes (BioRad). Membranes were probed with corresponding antibodies.

### Co-immunoprecipitations and 2D examinations of the proteins

Co-IPs and 2D examinations of proteins were performed as described^12,31^. For co-immunoprecipitations, we used the TrueBlot system which dramatically reduces signals of the immunoglobulins^12^. The protocol for this system was improved by an additional boiling of protein samples (in 2× loading buffer) for 30 min. Under these conditions, the native conformation of IgGs was destroyed and, in several experiments, IgG signals were not detectable. Full-length immunoblots corresponding to the blots shown in the main figures are presented in Supplementary Figs. 21–42.

### Real-time quantitative reverse transcriptase PCR

Total RNA was isolated as described^10^. TaqMan probes were purchased from Applied Biosystems. Human: β-actin: Hs01060665_g1, NR1H4: Hs01026590_m1, PSMD10: Hs01100439_g1, CYP3A4: Hs00604506_m1, PCK1: Hs01572978_g1, POU5F1: Hs04260367_gH, EPCAM: Hs00901885_m1, THY-1: Hs00264235_s1, AFP: Hs00173490_m1, HNF4α: Hs00230853_m1, RB1: Hs01078066_m1, TP53: Hs01034249_m1, CELF1: Hs00198069_m1, CEBPα: Hs00269972_s1, ALB: Hs00910225_m1, CDKN1A: Hs00355782_m1, RUNDC1: Hs00405433_m1, HACE1: Hs00410879_m1, MYO18B: Hs00261714_m1, PGAP1: Hs01088726_m1, REG3A: Hs01055563_gH, PARP1; Hs00242302_m1, Ku70: Hs01922655_g1, and Ku80: Hs00897854_m1.

### RNA-seq analyses of HBL samples

RNA samples were previously sequenced and reported in our previous publication^14^. As reported previously, RNA sequencing was performed for RNA isolated from 31 HBL patients and from 4 liver samples from healthy patients. RNA-sequencing libraries were prepared using Illumina TruSeq RNA preparation kit and sequenced on the Illumina HiSeq 2500, using paired end, 100 bp reads (Illumina, San Diego, CA). Reads were aligned using hg19 annotations produced by UCSC, and quantified using Kallisto. Statistical analysis was performed in GeneSpring 13.0. Thresholds were set at 1 for raw counts and normalized using quantile normalization procedure. Baseline was set to the median of all samples (*n* = 25,240 transcripts). A filter was applied to ensure analysis of reasonably expressed transcripts, requiring at least two reads in >50% of samples in at least one experimental condition (*n* = 12,551 transcripts). Ontological analysis of significantly differential genes was performed in the ToppGene Suite. RNA-sequencing data are available at the Gene Expression Omnibus (GEO) at NCBI with accession number GSE81928. In this manuscript, we have used these results for the analysis of groups of genes that belong to tumor suppressor proteins, stem cell markers, and markers of hepatocytes.

### HPLC-based examination of protein–protein complexes

Nuclear extracts from background and tumor sections of HBL samples were fractionated by size exclusion chromatography (SEC) using SEC400 column (BioRad) as described in our previous papers^29,51^. Optical density (280 nM) was monitored for each SEC run and compared for tumor and background sections. Location of C/EBPα and Rb in SEC fractions was determined by western blotting with specific Abs. For detection of protein–protein complexes, Rb was immunoprecipitated from SEC fractions and the IPs were probed with Abs to C/EBPα.

### Proliferation assay

HepG2, Huh6, and B-62 cells were seeded in 96-well plates at 3.0 × 10^4^ in 10% FBS DMEM with 1% pen/strep antibiotics. Cells were incubated at 37 °C in a CO_2_ incubator. Images were taken at 24 h after seeding prior to treatment. Cells were treated with 100 µM DPQ for 48 h, at 48 h post treatment, media was removed and cells were washed with 1× PBS. As per the CyQUANT Cell Proliferation Assay Kit (Invitrogen MP07026), 200 µL CyQUANT GR dye/cell lysis buffer was added to each well and incubated for 2–5 min at room temperature, protected from light. Fluorescence was measured using a fluorescence plate reader with filters for 480 nm excitation and 520 nm emission maxima.

### siRNA transfection

HepG2, Huh6, and B-62 cells were seeding in 10 cm dishes at 1.2 × 10^6^ cells per dish in antibiotic-free DMEM supplemented with 10% FBS. Cells were incubated at 37 °C in a CO_2_ incubator until cells reached 60% confluency. For each transfection, si-PARP1 siRNA duplex (0.5 µg) (Thermo Fisher Scientific, AM16708A) was diluted into siRNA Transfection Medium (Santa Cruz, sc-36868). Separately, siRNA transfection reagent (Santa Cruz, sc-29528) was diluted into siRNA transfection medium. The si-PARP1 siRNA transfection duplex was then added directly to the diluted transfection reagent and incubated at room temperature for 45 min. Additional siRNA transfection medium was then added to the transfection reagent mixture. Cells were washed once with siRNA transfection medium. After aspiration, transfection reagent mixture was added directly onto the washed cells. Cells were incubated for 7 h at 37 °C in a CO_2_ incubator at which time additional DMEM with 10% FBS and 1% antibiotics was added to the transfection mixture without removing transfection mixture. Protein was isolated as described elsewhere after 48 h.

### Computational identification of ALCDs

In our initial screens, the genomic sequence of C/EBPα was used to search for similar sequences located proximal to other tumor suppressor proteins of interest. Subsequently, exact matches to the 18BPS were identified in the human genome using the basic local alignment search tool (BLAST) available through the National Center for Biotechnology Information (NCBI) web portal. Identification of additional sequence properties of the full ALCD was achieved through investigation of the DNA sequence surrounding the 18BPS of individual genomic loci using the University of California Santa Cruz Genome Browser (GRCH37/hg19 human reference sequence). Genomic DNA spanning 200 base pairs upstream and 200 base pairs downstream of the 18BPS was analyzed. The resulting representative ALCD sequences and negative controls are provided in Supplementary Figs. 2–11.

### Statistical analysis

All values are presented as means ± SD. Differences between animal groups and background and tumor sections of HBL were determined using a Student's *t* test. A *p < 0.05 was considered statistically significant. ANOVA statistical analysis and dot plot graphs were generated with GraphPad Prism software. Dot plot graphs have error bars representing the standard error of the mean.

### Data availability

All data generated during this study are included in this published article and its Supplementary Information Files. We have also analyzed data of RNA-Seq which have been previously published in our paper^14^ and are available via NCBI GEO at accession number GSE81928.

## Electronic supplementary material

Supplementary Information

## References

[1] Kalish JM (2017). Surveillance recommendations for children with overgrowth syndromes and predisposition to wilms tumors and hepatoblastoma. Clin. Cancer Res..

[2] Trobaugh-Lotrario AD, Meyers RL, O’Neill AF, Feusner JH (2017). Unresectable hepatoblastoma: current perspectives. Hepat. Med..

[3] Czauderna P (2014). Hepatoblastoma state of the art: pathology, genetics, risk stratification, and chemotherapy. Curr. Opin. Pediatr..

[4] Meyer. A (2014). The natural history of clinically complete responders to neoadjuvant chemotherapy for urothelial carcinoma of the bladder. J. Urol..

[5] Eichenmüller M (2014). The genomic landscape of hepatoblastoma and their progenies with HCC-like features. J. Hepatol..

[6] Crippa S (2017). Mutant *CTNNB1* and histological heterogeneity define metabolic subtypes of hepatoblastoma. EMBO Mol. Med..

[7] Lee H (2017). General paucity of genomic alteration and low tumor mutation burden in refractory and metastatic hepatoblastoma: comprehensive genomic profiling study. Hum. Pathol..

[8] Jia D (2014). Exome sequencing of hepatoblastoma reveals novel mutations and cancer genes in the Wnt pathway and ubiquitin ligase complex. Hepatology.

[9] Martin J, Dufour JF (2008). Tumor suppressor and hepatocellular carcinoma. World J. Gastroenterol..

[10] Aguirre E, Renner O, Narlik-Grassow M, Blanco-Aparicio C (2014). Genetic modeling of PIM proteins in cancer: proviral tagging and cooperation with oncogenes, tumor suppressor genes, and carcinogens. Front. Oncol..

[11] Timchenko NA, Lewis K (2015). Elimination of tumor suppressor proteins during liver carcinogenesis. Cancer Stud. Mol. Med..

[12] Jiang Y (2013). FXR inhibits gankyrin in mouse livers and prevents development of liver cancer. Hepatology.

[13] Lewis K (2017). RNA binding protein CUGBP1 inhibits liver cancer in a phosphorylation dependent manner. Mol. Cell. Biol..

[14] Valanejad L (2017). FXR-Gankyrin axis is involved in development of pediatric liver cancer. Carcinogenesis.

[15] Wang C, Cheng L (2017). Gankyrin as a potential therapeutic target for cancer. Invest. New Drugs.

[16] Zhao X (2015). Gankyrin drives malignant transformation of chronic liver damage-mediated fibrosis via the Rac1/JNK pathway. Cell Death Dis..

[17] Huang SJ (2017). Inducible liver-specific overexpression of gankyrin in zebrafish results in spontaneous intrahepatic cholangiocarcinoma and hepatocellular carcinoma formation. Biochem. Biophys. Res. Commun..

[18] Feng FY, de Bono JS, Rubin MA, Knudsen KE (2015). Chromatin to clinic: the molecular rationale for PARP1 inhibitor function. Mol. Cell.

[19] Schiewer MJ (2012). Dual roles of PARP-1 promote cancer growth and progression. Cancer Discov..

[20] Gibson BA (2016). Chemical genetic discovery of PARP targets reveals a role for PARP-1 in transcription elongation. Science.

[21] Goodwin JF, Knudsen KE (2014). Beyond DNA repair: DNA-PK function in cancer. Cancer Discov..

[22] Roper SJ (2014). ADP-ribosyltransferases Parp1 and Parp7 safeguard pluripotency of ES cells. Nucleic Acids Res..

[23] Wang C (2013). Poly(ADP-ribose) polymerase 1 promotes oxidative-stress-induced liver cell death via suppressing farnesoid X receptor α. Mol. Cell. Biol..

[24] Simbulan-Resenthal C (2003). PARP1 binds E2F-1 independently on its DNA binding and catalytic domains, and acts as a novel coactivator of E2F1-mediated transcription during re-entry of quiescent cells into S phase. Oncogene.

[25] Mostocotto C (2014). Poly(ADP-ribosyl)ation is required to modulate chromatin changes at c-MYC promoter during emergence from quiescence. PLoS ONE.

[26] Gottschalk AJ (2009). Poly(ADP-ribosyl)ation directs recruitment and activation of an ATP-dependent chromatin remodeler. Proc. Natl Acad. Sci. USA.

[27] Yang L (2013). Identification of poly(ADP-ribose) polymerase-1 as a cell cycle regulator through modulating Sp1 mediated transcription in human hepatoma cells. PLoS ONE.

[28] Yao D, Pengm S, Daim C (2013). The role of hepatocyte nuclear factor 4alpha in metastatic tumor formation of hepatocellular carcinoma and its close relationship with the mesenchymal-epithelial transition markers. BMC Cancer.

[29] Wang GL, Iakova P, Wilde M, Awad S, Timchenko NA (2004). Liver tumors escape negative control of proliferation via PI3K/Akt-mediated block of C/EBPα growth inhibitory activity. Genes Dev..

[30] Wang GL, Timchenko NA (2005). Dephosphorylated C/EBPα accelerates cell proliferation through sequestering retinoblastoma protein. Mol. Cell. Biol..

[31] Wang GL (2010). Elimination of C/EBPα through the ubiquitin-proteasome system promotes the development of liver cancer in mice. J. Clin. Invest..

[32] Cozzolino AM (2013). TGFβ overrides HNF4α tumor suppressing activity through GSK3β inactivation: implication for hepatocellular carcinoma gene therapy. J. Hepatol..

[33] Abella A (2015). Cdk4 promotes adipogenesis through PPARgamma activation. Cell Metab..

[34] Vaughan C, Pearsall I, Yeudall A, Deb SP, Deb S (2014). p53: its mutations and their impact on transcription. Subcell. Biochem..

[35] Lee MG (2014). XAF1 directs apoptotic switch of p53 signaling through activation of HIPK2 and ZNF313. Proc. Natl Acad. Sci. USA.

[36] Tortola L (2016). The tumor suppressor Hace1 is a critical regulator of TNFR1-mediated cell fate. Cell Rep..

[37] Muñoz-Gámez JA (2015). Synergistic cytotoxicity of the poly (ADP-ribose) polymerase inhibitor ABT-888 and temozolomide in dual-drug targeted magnetic nanoparticles. Liver Int..

[38] Quiles-Perez R (2010). Inhibition of poly adenosine diphosphate-ribose polymerase decreases hepatocellular carcinoma growth by modulation of tumor-related gene expression. Hepatology.

[39] Cast A (2017). C/EBPα-dependent pre-neoplastic tumor foci are the origin of hepatocellular carcinoma and aggressive pediatric liver cancer. Hepatology.

[40] Landers ES (2001). Initial sequencing and analysis of the human genome. Nature.

[41] Jordan IK, Rogozin IB, Glazko GV, Koonin EV (2003). Origin of a substantial fraction of human regulatory sequences from transposable elements. Trends Genet..

[42] Hambor JE, Mennone J, Coon ME, Hanke JH, Kavathas P (1993). Identification and characterization of an Alu-containing, T-cell-specific enhancer located in the last intron of the human CD8 alpha gene. Mol. Cell. Biol..

[43] Xie M (2013). DNA hypomethylation within specific transposable element families associates with tissue-specific enhancer landscape. Nat. Genet..

[44] Yang Z, Boffelli D, Boonmark N, Schwartz K, Lawn R (1998). Apolipoprotein(a) gene enhancer resides within a LINE element. J. Biol. Chem..

[45] Bejerano G (2006). A distal enhancer and an ultraconserved exon are derived from a novel retroposon. Nature.

[46] Feschotte C (2008). Transposable elements and the evolution of regulatory networks. Nat. Rev. Genet..

[47] Mukhopadhyay P (2014). Poly (ADP-ribose) polymerase-1 is a key mediator of liver inflammation and fibrosis. Hepatology.

[48] Mukhapadhyay P (2017). PARP inhibition protects against alcoholic and non-alcoholic steatohepatitis. J. Hepatol..

[49] Bissig-Choisat B (2016). Novel patient-derived xenograft and cell line models for therapeutic testing of pediatric liver cancer. J. Hepatol..

[50] Liang C, Park A, Guan J (2007). *In Vitro* scratch assay: a convenient and inexpensive method for analysis of cell migration *in vitro*. Nat. Protoc..

[51] Iakova P, Timchenko LT, Timchenko NA (2011). Intracellular signaling and hepatocellular carcinoma. Semin. Cancer Biol..

