# Supplementary material for: PARP1 activation increases expression of modified tumor suppressors and pathways underlying development of aggressive hepatoblastoma
Source: Commun Biol. 2018 Jun 11;1:67. doi: 10.1038/s42003-018-0077-8 (PMC6123626; doi:10.1038/s42003-018-0077-8)
Supplement: Supplementary file 1 — Supplementary Information [file 42003_2018_77_MOESM1_ESM.pdf]

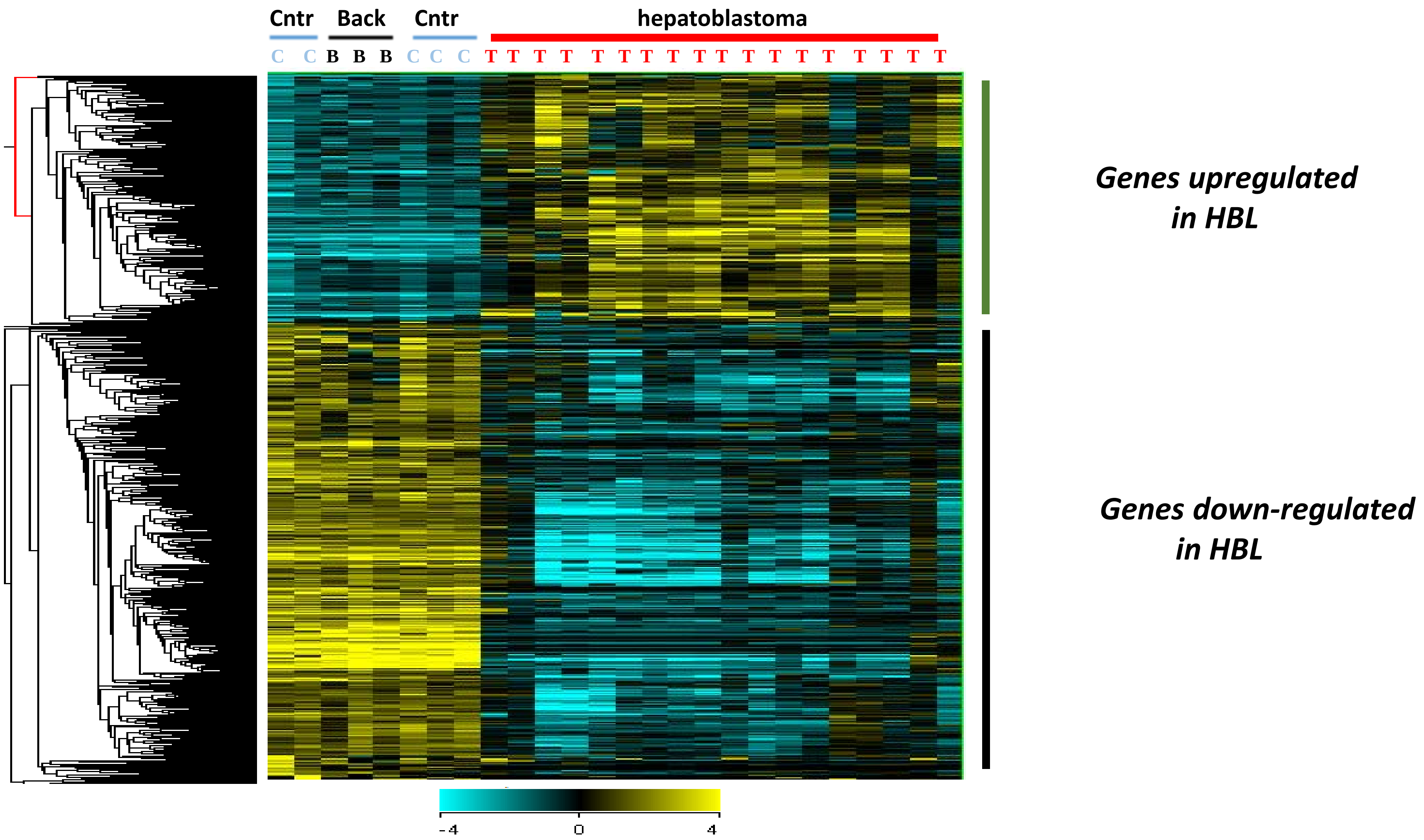

Supplementary Figure 1. Heat map of RNA-Seq analyses performed with 31 HBL samples from patients with classic hepatoblastoma.
Cntr: control livers, Back: background regions of livers from patients with HBL. Hepatoblastoma RNA-Seq with tumor sections of the liver. More information about differentially expressed genes can be found in our recent paper Valanejad et al. Carcinogenesis 2017, ref #14.

Whole Exome Sequencing of Aggressive Hepatoblastoma<br>**Mutations**	
**<u>Tumor Suppressor Proteins</u>:**<br>**C/EBPα, HNF4α, p53, CUGBP1 & RB**	None
**<u>Additional Representative Genes</u>**	NFE2L2, MDM4, ARID1A, ARSD, MAGEB16, SLC25A5, UBE2NL

Supplementary Figure 2. Mutations observed in aggressive HBL samples used in our studies.
No mutations in 5 TSPs were found.

CUGBP1 Sequence 1: chr11:47567333-47567750
CTCCTATTTTTCTCCCTTAAAAAGATGTAGGGAGGCCGAGGCAGGCAGAT
CACAAGGTCAGGAGATCGAGACCATCCTGGCTAACACG**GTGAAACCCCGT**
CTCTACTAAAAATACAAAAAATTAGCTGGGCATGGTGGCAGGCACCTGTA
GTCGCAGCTACTTGGGAGGCTGAGGCAGGAGAATGGCGTGAACCCGGGAG
GCGGAGCTTGCAGTGAGCTGAGATGGCACCACTGCACTCCAGCCTGGGTG
AGAGTGGGAGACT**CCATCTCAAAAAAAAAAAAAATATATATATACA**CACAC
ACACACACACACACACACACACACACACGCAGACACACACACACACACGT
ATACATATGAGAGAGAGGGGCTGGGCATGGTGGTGCATGCCTGTAATCCC
AGCACTTTGGAAGACTGA

CUGBP1 Sequence 2: chr11:47534882-47535299
CGCAGTGGCTCACGCCTGTAATCCTAGCACTTTGGGAGGCCGAGGTGGGC
GGATCACGAGGTCAGGAGATCGAGACCATCCTGGCTAACATG**GTGAAACC**
CCATCTCTACTAAAAATACAAAAAATTAGGCGTGGTGGCGGGTGCCTGTA
GTCCCAGCTACTCGGAAGGCTGAGGCAGGAGAATGGCATGCACCCGAGAG
GCGGAGCTTGCAGTGAGCTGAGATCACGCCACTGCACTCCAGCCTGGGTG
ACAGAGCGAGA**CTCTGTCTCAAAAAAAAAAAAAAGAG**GGGGTGCTGCCCAC
ATGTTGCTTTTACAGTACCTGCAATTTCAGGGGGGAAAACCCCCAAAAAA
CTAGGAAGTGCAAGTATGTCTCAATTCAGGAAAGTTCATAGAAACTTTAC
AAATTACACCAGATAAAC

P53 Sequence 2: chr17:7598141-7598558
GCTCACGCCTGTAATCCCAGCACTTTGAGAGGCCAAGGCGGGCGGATCAC
GAGGTCAGGAGATCGAGACCATCCTGGCTAACATG**GTGAAACCCCGACTG**
TACTAAAAATACAAAAAATTAGCCTGGCGTGGTGGCAGTGGGTGCGTGTA
GTCCCAGCTACTCGAGAGGCTGAGGCAGGAGAATGGCGTGAACCCGGGAG
GCGGAGCTTGCAGTGAGCTGAGATCGCGCCACTGCACTCCAGCCTGGGGG
ACACAGCAAGACTCCA**TCTCAAAAAAAAAAAA**GTTATCTATTTCAAATGCT
AAGCTTGTAGACAC**TAAAAATGAAAATACCTAAT**GAGAGAGGTTGCGATA
GAACTGGTACACTAGCAGG**AAAAAAAAAAAAAAAAAGCACAAAAA**GGTTTT
GGTTGTTTGTTTTGAGAC

HNF4α Sequence: chr20:42998537-42998954
TGGCTCACGCCTGTAATCCCAGCACTTCGGGAGGCCGAGGCGGGCAGGTC
ACAAGGTCAGGAGATCGAGACCATCCTGGCTAACACG**GTGAAACCCCGTC**
TCTACTAAAAATACAAAAAATTAGCCGGGCGCAGTGGCGGGCGCCTGTAG
TCCCAGCTACTTGGGGGGGCTGAGGCGGGAGAATGGCGTGAACCCGGGAG
GCGGAGCTTGCAGTGAGCCGAGATCGCGCCACCGCACTCCAGCCTGGGTG
AAAGAGTGAGACTCCGT**CGCAAAACAAACAAACAAACAAAAAAAAACAAAA**
GTCCAGTGTGGCTGACTGCAGTGACCAAAGGGAGAAAGACGAGAGACCAT
CAGACAAGAGTCAGAGCCTGTGAGAGCCTTTTGGTCAAAGAAAAGAGCCC
AGGGCCGGGCGCGGCGGC

C/EBPα Sequence: chr19:33793460-33793877
TGGTTCTGGCTTTGAAAGAGAATCCGCGCCCCAGCAGCTCAAGACCAAG
ACTCGCCCTCCGCCCCCCACCCCTACCCCGTGCAGCCTCGGGATACTCCT
GGGCTCCCGGCCGTGGCTGGATACGGGCGCCTAGGGCAGGCAGGAGGAGG
GGGCCCCCGCTACCGACCACGTGGGCGCGGGGGCGACGGCCGGGCCGGGG
GCGGAGCTTGGAGCGAGCGCCGCGGCTCTGCTGGGCGCGCTGGAGGCGGT
GGGCGTTGCGCCGCGGCCTGCCTGGGGAGCGCGGCGCTGTGCCGCGCTGG
TTCGCCGCCCCATGCCGGCCGCGCGCTAGGACCCAGCAGGCGCCGCGCCG
CCGCAGCCCGGGGACAGAGGCCGCCTCGGACTCTAGGGGGCGACGCGGCC
TGCCGGGTATAAAAGCTG

RB Sequence: chr13:48887761-48888172 76% CONSERVED
GTGGCTCACGCCTGTAATCCCAGCACTTTGGGAGGCCGAGGTGGGCGATC
ACGAGGTCAGGAGATCGAGACCATCCTGGCTAACACA**GTGAAACCCCGTC**
TCTACTAAAAATACAGAAAAATTAGCCGGGCGTGGTGGCAGGCACCTGTA
GTCCCAGCTCTTTGGGAGGCTGAGGCAGGAGAATGGCATGAACCCGGGAG
GCGGAGCTTGCAGGAAGCCGAGATTGTGCCACCGCACTCCAGCCTGGGCG
ACAGAGCGAGACTCCG**TCTCAAAAAAAAAAAAAAAAAAAAAAGAATA**AACA
TACAGAGCAATGGAATAGAACTGAGACTCCAGAAATAAACCCTGGTTTTT
ATAGTCAACTGATTTTCATCAAGGGCACCAAAACAATTCAATGAAGAAAG
AATAGTCTTTTC

Supplementary Figure 3. Nucleotide sequences of ALCDs of five TSPS: CUGBP1, p53, HNF4α, C/EBPα and Rb. 100% homologous 18BP sequence is shown in red. 18BP sequence is located in the coding region of C/EBPα which does not have a strong homology for other regions of ALCD.

PMPCA: chr9:139315732-139316149
GCTCCCGCCTGTAATCCCAGCTCTTTGGGAGGCTGAGGCAGGCGGATCAC
GAGGTCAGGAGATTGAGACCATCCTGGCTAACAGAGTGAAACCCCTTCTC
TACTAAAAATACAAAAAGAAATTAGCTGGGCGTGGTGGCGGGCGCCTGTA
GTCCCAGCTGCTCAGGAAGCTGAGGCGGGAGAATGGCATGAACCCGGGAG
GCGGAGCTTGCAGTGAGCCCAGATCGCACCACTGCACTCCAGCCTGGGCG
ACAGAGCAAGACTCTG**TCTCAAAAAAAAAAAAAAAAAAAAAAAAC**GGGAAC
CCAGGCCCAAGTCACAGGCAACCCTGCTGGTCTCCCCTTCAGGTTTTTA
GGGAAGGGCTGTGCTACGTGTTACCCGGCTTCTACCACACTTGAAATACA
GGAGCAATGTGCCTCCCT

RUNDC1: chr17:41138385-41138802
AGCTCACGCCTGTAATCCCAGCACTTTGGGAGGCCGAGGCGGGTGGATCA
CGAGGTCAGGAGATTGACCATCCTGGCTAACACAGTAAAACCCCGTCTCT
ACTAAAAATACAAAAAAAAAATTAGCCGGGTGTGGTGGCGGGCGCCTGTA
GTCCCAGCTACTTGGGAGGCTGAAGCAGGAGGATGGCGTGAACCCGGGAG
GCGGAGCTTGCAGTGAGCAGAGATCGCGCCACTGCACTCCAGCCTTGGTG
ACAGAGCGAGACTCCG**TCTCAAAAAAAAAGAAAAAAACTCATAA**CCAGCT
TTCCTAAGGTTCACCAAAGTCCCTTTGATGTACGATAGACCACTTCTGAA
AAATCAAAAGAATCAAACGCACCAGGAGTGGTGTCTCACGCCTGTAACTG
CAGCACTCTGGGAGGCCA

RUNDC1: chr17:41143962-41144379
TGGCTCACACCTGTAATCCCAGCACTTTGGGAGGCCGAGGCGGGCGGATC
ACAAGGTCAGGAGTTCGAGACCATCCTGTCTAACACGGTGAAACCCCATC
TCTACTAAAAATACAAAAAAATTAGTCGGACATGGTGGCAGGCACCTGTA
GTCCCAGCTACTCGGGAGGCTGAGGCAGGAGAATGGCGTGAACCCAGGAG
GCGGAGCTTGCAGTGAGCCAAGATCGCGCCACTGCACTCCAGCCTGGGCG
ACAGAGTGAGACTCCG**TCTCAAAAAAAAAAAGAA**GGTTGTACCCACAGTA
TACGTGTGGGCACTTGTGCTTGAGCCTGTATTAAAGGAATCAGGCCAGGC
ACAGTGGCTCACGCCTGTAATCTCAGCACATTGGGAGGCTGAGGTGGGCG
GATCACCTGAGGTCAGGA

PGAP1: chr2:197751838-197752255
GTCAATTAAAAGCAGTGTGACCGCACTGTGGGAGGCCAAGGCAGGCGGAT
CACAACATCAGGAGATCGAGACCCTCCTGGCTAACACGGTGAAACCCCGT
CTCTGCTAAAAATACAAAAAATTAGCTGGGCGTGGTGGTGGGCGTCTGTA
GTCCCAGCTACTCAAGAGGCTGAGGCAGGAGAATGGCATGAACCTGGGAG
GCGGAGCTTGCAGTGAGCCGAGATCGCGCCACTGCACTCCAGCCTGGGCG
ACAGAGCGAGACTCCA**TCTCAAAAAACAAAACAAAACAAAACAAAAACA**A
ACAAACAAAAACAGCAGTGTGACCTGAGATCTTAACTGCAACTTTTAAGG
AAGTAAGAGCAATGTTCTGAGATATGATTATCTAACTAGTGAAATCCTAC
CAAGACATAAGAGGATGA

PGAP1: chr2:197706112-197706529
GTGGCTCACGCCTGTAATCCCAGCATTTTGGGAGGCCAAGGCGGGTGGAT
CACGAGGTCAGGAGATCGAGACCATCCTGGCTAACACAGTGAAACCCCGT
CTCTACTAAAAATACAAAAAATTAGCCAGGTGTGGTGGCGGGCGCCTGTA
GTCCCAGCTACTCGGGAGGCTGAGGCAGGAGAATGGCATGAACCCGGGAG
GCGGAGCTTGCAGTGAGCCGAGATTGCGCCACTGCACTCCAGCCTGGGCA
ACAGAGTGAGACTC**CATCTCAAAAATAAAAAAAGAAATTATGAATAG**GTA
AATTCATATCATTAAAAATTCATTTAAATTCACTTAGAATATTTATCTTT
CCAAAACTGTATAAAAGATTGATATAATTTTGGCTCACAATGTAGTTCAC
ATAAGTAATGTATATTTC

PGAP1: chr2:197759256-197759673
GTGGCTCATGCCTGTAATTCCAGCACTTTGGGAGGCCAAGACGGGTGGAT
CACAAGGTCAGGAGATCGAGACCATCCTGGCTAACACGTAGAAACCCTGT
CTCTACTAAAAATACAAAAAATTAGCCGGGTATGGTGGTGGGCACCTGTG
GTTCCAGCTACTCGGGAGGCTGAGGCAGAAGAATGGCATGAACCCGGGAG
GCGGAGCTTGCAGTGAGCCGAGATCGCGCCACTGCACTCCAGCCTGGGTG
ACAGAGCAAGACTCCGT**CTCAAAAAACAAAAACAAAAACAAAAACAAAAA**
AACTTTAAGACTGGATTCCACCATTCTTACTCACTCTCAAGGTAATCCAT
CTACTCACAAGTTTTTAGCTATGACTTTTATGCTAATAACCACTAGAAAT

Supplementary Figure 4. Nucleotide sequences of ALCDs of RUNDC1, PMPCA1 and PGAP1.

PGAP1: chr2:197696815-197697232
ATGCCTGTAATCCCAGCACTCTGGGAGGCCGAGGCAGGCGGATCACAAGG
TCAGGAGATCGAGACCATCCTGGCTAACACAGTGAAACCCCGTCTCTACT
AAAAAAAAAAAAATACAAAAAATTAGCTGGGCCTGGTGGCGGGCACCTGTA
GTCCCAGCTACTTGGGAGGCTGAGGCAGGAGAATGGCGTGAACCCGGGAG
GCGGAGCTTGCAGTGAGCTGAGATCGCGCCACTACACTCCAGCACTCCAG
CCTGGGAGACAGAGCCAGACTCCGT**CTCCAAAAAAAAAAAAAAAAGAAAAA**
AGAAATGTGAAAAGCATAGTCTCTGGGAGAAAAATATCTACAATTCTTTA
AAACAGAATGTGAACCATGAAATGTTCATACAAATGCATGTGTGTGGAAA
ACTACAAAATACAAAGTG

HACE1: chr6:105221835-105222252
TGGCTCACGCCTGTAATCCCAGCACTTTGGGAGGCCGAGGCGGGCGGATC
ACGAGGTCAGGAGATCGAGACCATCCCGGCTAAAACGGTGAAACCCCGTC
TCTACTAAAAATACAAAAAAATTAGCCGGGCGTAGTGGCGGGCGCCTGTA
GTCCCAGCTACTTGGGAGGCTGAGGCAGGAGAATGGCGTGAACCCGGGAG
GCGGAGCTTGCAGTGAGCCGAGATCCCGCCACTGCACTCCAGCCTGGGCG
ACAGAGCGAGACTCCGT**CTCAAAAAAAAAAAAAAAAAAAAAAAAAAAAGAG**
AAAAGATGATAACCTATTTTTTGTAATGTGGTAAGAACTCAGTTAGCTGA
TATGTGAAAATGCTTAGTTGGTGCCTGGCATATAGAAAGTATAAATATTA
TATGCTATTATTATTACA

HACE1: chr6:105279498-105279915
GTGGCTCACGCCTGTAATCCCAGCATTTTAGGAGGCCGAGGCAGGCGGAT
CATGAGGTCGGGAGATTGAGACCATCCTGGCTAACGCAGTGAAACCCCAT
CTCTACTAAAAATACAAAAAATTAGCCGGGCGTGGTGGCCGGTCCCTGTA
GTCCCAGCCACTCGGGAGGCTGAGGCAGGAGAATGGCATGAACCCGGGAG
GCGGAGCTTGCAGTGAGCCGAGATGGCGCCACTGCACTCCAGCCTGGGGA
ACAGAGTGAGACTCCAT**CAAAAAAAAGAGAGAGAAAGAAAAGAGAAAGAA**
GAGAAGAGAAGAGAAGAGAAGAGAAGAGAAGAGAAGAGAAGAGAAGAGAA
GAGAAGAGAGGAAGGAAGGAAGGAAGGAAGGAAGGAAGGAAGGAAGGAAG
GAAGGAAGGAGAAAGAAA

HACE1: chr6:105297394-105297811
GGCTTACGCCTGTAATCCTAGCACTTTGGGAGGCCGAGGCGGGCGGATCT
TGAGGTCAGGAGATTGAGACCATCCTGGCTAACATGGTGAAACCCCGTCT
CTGCTAAAAATACAAAAAAAAGTAGCCTGGCATGGTGGCAGGTGCCTGTA
GTCCCAGCTACTCGGGAGGCTGAGGCAGGAGAATGGCGTGAACCCGGGAG
GCGGAGCTTGCAGTGAGCTGACATCGCACCACTGCACTCCAGCCTGGGTG
ACATGCGAGACTCTGT**CTCAAGAAAAAAAAAAAAAAT**GTGTTTAAGGCCAG
GCATGGCGGCTCACACCTGTAATCCCAGCACTTTGGGAGGCTGAGGTAGG
AGGATTGCCTGAGTTCAGGAGTTTGAAATCAGCCTGGGCAACATAATGAG
GTCTCTTCTCTACAGAAA

MYO18B: chr22:26148025-26148442
GTGGCTCACGCCTGTAATCCCAGCACTTTGTGAGGCCGAGGCGGGCGGAT
CACGAGGTCAGGAGATCGAGACCATCCCGGCTAAAACGGTGAAACCCCGT
CTCTACTAAAAATACAAAAAATTAGCCGGGCGTAGTGGCGGGCGCCTGTA
GTCCCAGCTACTTGGGAGGCTGAGGCAGGAGAATGGCGTGAACCCGGGAG
GCGGAGCTTGCAGTGAGCCGAGATCCCGCCACTGCACTCCAGCCTGGGCG
ACAGAGCGAGACTCCG**TCTCAAAAAAAAAAAAAAAAATTAATTAAAATAA**
AAGTTAAATATTTGTCAAATGAATAAGTAGGTGAATGAATGAACAGTTAG
AAATGAGCCTGAGAAGGGACAGAGCAGCAAGTCTGTGGGCAGAGATTTCT
GATGTCCCCCTGTGCTGT

MYO18B: chr22:26177307-26177724
TGGCTCACGCCTGTAATCCCAGCACTTTGGGAGGCTGAGACGGGTGGATC
ATGAGGTCAGGAGATTGACACTATCCTGGCTAATGCGGTGAAACCCCGTC
TCTACTAAAAAAACAAAAAAATTAGCCGGGCGTGGTGGTGGGCGCCTGTG
GTCCCAGCTACTCGGGAGGCTGAGGCAGGAGAATAGCGTGAAGCTGGGAG
GCGGAGCTTGCAGTGAGCCGAGATCACGCCACCGCACTCCAGCCTGGGCA
ACAGAGCGAGACTCCGT**CTCAAAAAAAAAAAAAAAAAGAGT**GGATCAGAGT
GGGGCACCCCAATGGGGCTTCTGGGTAGCTGCACTTCAGGCATGTGCAGA
TGTACCCAAAGCACTGACTGGGTGCCCCTCTTCTCTCCCTGCAGACAATG
CTTTTGGAGAAGAGCCGC

Supplementary Figure 5. Nucleotide sequences of ALCDs of PGAP1, HACE1 and Myo188.

MYO18B: chr22:26205935-26206352
TGGCTCACACCTGTAATCCCAGCACTTTGGGAGGCTGAGGCAGGCGGATC
ACGAGGTCAGGAGCTCGAGACCATCCTGGCTAACACAGTGAAACCCCATC
TCTACTAAAAATACAAAAAAAACATCTGGGCATGGTGGTGGGCGCCTGTA
GTCCCAGCTACTTGGGAGGCTGAGGCAGGAGAATGGCATGAACCCGGGAG
GCGGAGCTTGCAGTGAGCCGAGATGGTGCCACTGCATTCCAGCCTGGGCA
ACAGAGCAAGACTCTGTC**TCAAAAAAAAAAAAAAAAAG**CCTGAAGGAAGG
GTATACCCCAAACTGTTCACAGTGGTCACCTTTCATAGCAATGGGATTTG
GCACTGGGGTGTGAGGAAAGACCTTTTTTTTTTTTTAAACTTTCTATTC
TTCTGGACTATTTCCATT

MYO18B: chr22:26217474-26217891
GGGGCTCACGCCTGTAATCCCAGCACTTTGGGAGGCTGAGGTGGGCAGAT
CATGAGGTCGGGAGATGGAGACCATCCTGGCTAACACAGTGAGACCCTGT
CTCTACTAAAAATACAAAAAATTAGCTGGGCGTGGTGGTGGGTGCCTGTA
GTTCCAGCTACACGGGAGGCTGAGGCAGGAGAATGGTGTGAACCCAGGAG
GCGGAGCTTGCAGTGAGCTGAGATTGTGCCACTGCACTCCAGCCTGGGCG
AGAGTGAGACTCCAT**CTCAAAAACAAAACAGAACAAAACAAAACAAACAA**
AAAAAAGAAGTCATTTATTTTTCTATGGATTAAAAAAGTAGTGCTCAACC
ATGGTAGAAAATTTGGAAAATAAAAAATCCCTTTACCTAGCTGTGATGGT
TAATATTTAGCCATATTT

MYO18B: chr22:26263467-26263884
GGCTCACGGCTGTTATCTCAGCACTTTGGGAGGCTGAGGTGGGCGGATCA
CGAGGTCAGGAGATCGAGACCATCCTGGCTAACATGGTGAAGCCCCGTCT
CTACTGAAAATACAAAAAATTATAGTCGGGCATGGTGGCGGGCGCCTGTA
GTCCCAGCTACTCGGGAGGCTGAGGCAGGAGAATGGCATGAACCTGGGAG
GCGGAGCTTGCAGTGAGCCGAGACTGCACCACTGCACTCCAGCCTGGGCA
ACAGAGCGAGACTCCG**TCTCAAAAAAAAGAAAGAAAGAAAGAAAGAAATC**
AGGGTCTTGAAGAGATATTTATATATTTGTGTTCATAGCAGCATTATACT
CAATACCCAAAATGTGGAAGCAACCCAAATGTCCATTGATGGATGAATGG
ATAAACACTATGTGGTCT

MYO18B: chr22:26309201-26309618
TCACGCCTGTAATCCCAGCACTTTGGGAGGCCAAGGCGGGTGGATCACGA
GGTCACGAGATCGAAACCATCCTGGCTAACACGGTGAAACTGTGTCTCTA
CTAAAAATACACACAAAAAAATTAGCCAGGCGTGGTGGCGGGCGCCTGTA
GTCCCAGCTACTCAGGAGGCTGAGGCAGGAAAATGGCATGAACCCAGGAA
GCGGAGCTTGCAGTGAGCCCAGATCACACCACTGCACTCCAGCCTGGGAG
GCAGAGCGAGACTCCG**TCTCAAAAAAAAGAAAAGAAAAGAAAAAG**CCACAA
TCAACATGTGAAAAATGCACAGCATCCCTGTGGAGGGCCACGCAATACCA
TTTAGCACCCGATGCCTGGAAAAAATGTTTAAAAATGTCTGACAGTGCCA
CATGTTGGTGGGGACATG

FBXL18: chr7:5514209-5514626
TGGCTCACGCCTGTCATCCCAGCACTTTGGGAGGCCGAGGCGGGCGGATC
ACGAGGTCAGGAGATCAAGACCACCCTGGCGAACACGGTGAAACCCCGTC
TCTACTGAAAATACAAAAATATTAGCTGGGCGTGGTGGCGGGCGCCTGTA
GTCCCAGCTGCTCGGGAGGCTGAGGCAGGAGAATGGCCTGAACCCGGGAG
GCGGAGCTTGCAGTGAGCCGAGATCGCGCCACTGCACTCCAGCCTGGGTG
ACAGAGCGAGACTCTGTCTC**AAATAAATAAATAAATAAATAAATAAATAA**
ATAAATAAAAAATAAAGTGCATAATTTGCCAGCGGAGGATTTTTTTGAAG
AAAAAAAAGTAAAATAAAGTGCATAGTTCAAGCTGGGGGCGGTGGCTCAT
GCTTGTAATCCCAGCACT

FBXL18: chr7:5516389-5516806
GTGGCTCACGCCTGTAATCCCAGCACTTTAGGAGGCTGAGGCAGGCGGAT
CATGAGGTCAGGAGATCGAGACCGTCCTGGCTAACACAGTGAAACCCCGT
CTCTACTAAAAATACAAAAAAGTAGTCGGGTGTGGTGGCGGGCGCCTGTG
GTCCCAGCCACTCAGGAGGCTGAGGCAGGAGAATGGCATGAACCTGGGAG
GCGGAGCTTGCAGTGAGCTGAGATCGCACCACTGCATTCCAGCCTGGGCG
ACAGAGTGAGACTCCG**TCTCAAAAAAAAAAAAAAACAAAAGAAG**TTTCTAG
ATCTACTGGGCATGATGAACACAAACCCCACAGACACTGAGGAACCCAGT
GGTGGCAGTGACTCGGGCTCCTCTGCTCTCTAAAGCTCCTTTGAGAAACA
TGGGAGGGGCCGGGCGTG

Supplementary Figure 6. Nucleotide sequences of ALCDs of Myo18B and FBXL18.

PAX2: chr10:102517768-102518185
GTGGCTCACGCCTGTAATCCCAGCAATCTGGGAGGCCGAGGCGGGCGGAT
CACGAGGTCAGGAGATCGAGACCATCCCGGCTAACATGGTGAAACCCCAT
CTCTACTAAAAATACAAAAAATTAGCCGGGCGTGGTGGTGGGCGCCTATA
GTCCCAGCTACTCGGGAGGTTGAGGCAGGAGAATGGCGTGAACCCGGGAG
GCGGAGCTTGCAGTGAGCTGAGATCGTGCCATTGCACTCCAGCCTGGGCA
ACAGAGCAAGACTCCGTCTCAAAAAAAAAAAAAAAAGAATGGCACCTGACT
CGCTCCCCCTGCCCTGCTCTCTTTCTATCTCTTTCCTTCTCCCTTGGAAA
ACACAAAATCTGTTCTCATTACATTATCTCTCAGAAAACAAAAGAAAGGG
CCTCATTTCTCCCATGGT

FAM46D: chrX:79597081-79597498
GTGGCTCACGCCTGTAGTCCCAGCACTTTGGGAGGCCGAGGCGGGCGGAT
CACGAGGTCAGGAGATCGAGACCATCCTGGCTAACACGGTGAAACCCCGT
CTCTACTAAAAATACAAAAAATTAGCCGGGCGTGGTAGCGGGCGCCTGTA
GTCCCAGCTACTCGGGAGGCTGAGGCAGGAGAATGGCGTGAACCCGGGAG
GCGGAGCTTGCAGTGAGCCGAGATCGCGCCACTGCACTCCAGCCTGGGCG
ACAGAGCGAGACTCCGTCTCAAAAAAAAAAAAAAAAAAAAGAAAAGAATTA
TTTTCAGTATACCTCAGCTACAACTGACAAACAATGGTTTTTACAATTCT
GAGGTTTAGAGAGGATGAAAGCATACTACCAACCTAGATCAGAGCTAGCT
ATGCAAATCAGATAGGAA

FAM46D: chrX:79647886-79648303
GTGGCTCACGCCTGTAATCCCAGCACTTTGGGAGGCCGAGGCGGGTGGAT
CATGAGGTCAGGAGATCGAGACCATCCTGGCTAACAAGGTGAAACCCCGT
CTCTACTAAAAATACAAAAAATTAGCCGGGCGCGGTGGCGGGCGCCTGTA
CTCCCAGCTACTCGGGAGGCTGAGGCAGGAGAATGGCGTGAACCCGGGAA
GCGGAGCTTGCAGTGAGCCGAGATTGCGCCACTGCAGTCCGCAGTCCGGC
CTGGGCGACAGAGCGAGACTCCGTCTCAAAAAAAAAAAAAAAAAAAAAAAA
AAAAAAAAAGAATAAAATGAGAATAAAGCTATAAAATATTAAAAATTTGA
AACCAAGTAAGATCACAGAAATTGGCCCATTAAGAATGGTCTTCAATAGT
GCCTCAAAGTTATTTGTG

KRT86: chr12:52675887-52676304
CTCACGCCTGTAATCCCAGCACTTTGGGAGGCTGAGGCGGGCGGATCACG
AGGTCAGAAGATCGAGACCATCCTGGCTAACACAGAGAAACATCGTCTCT
ACTAAAAATACAAAAACAAAATTAGCCCATCACGGTGGCAGGCGCCTGTA
GTCCCAGCTATCTGGGAGGCTGAGGCGGAAGAATGGTGTGAACCCGGGAG
GCGGAGCTTGCAGTGAGCCAAGATTGCGCCACTGCAGTCCAGCCTGGGCG
ACAGAGCAAGGCTTTGTCTCAAAAAAAAAAAAAATTGTTTCAGGGGGCTC
AAAAACCTTCTAAAGAATTCCAGTAAGTGAAATAATAGTTACACTTACAT
AACTTCCTATGCTTTAAGCACTTTACATGCAGCAATACCTTTTCTCAAAC
AACCCTCTGATAGTTACT

WNT98: chr17:44960215-44960632
CTCACGCCTGTAATCCCAGCACTTTGGGAGGCTGAGGTGGGCGGATCACG
AGGTCAGGAGATCAAGACCATCCTGGCTAACGTGGTGAAACCCCGTCTCT
ACTAAAAATACAAAAACAAAATTAGCCGGGCGTGGTGGCGGGCACCTGGA
GTCCCAGCTACTCGGGAGGCTGAGGCAGGAGAATGGCATGAACCTGGGAG
GCGGAGCTTGCAGTGAGCAGAGATCATGCCACTGCACTCCAGCCTGGGAG
ACAGAGGGAGACTCTGTCTCAAAAAAAAAAATGGGGATCATGGTTGTATG
GACCCCTGGGGTTGCCCAGAGGGGAGTATGAGGAAATGGCCTGCACAGGA
ATGCCTGTGTGGGGTGGAGCCTGCACAGAGCCACCTATTGGAGTGCTGGA
CACCGTGCATGCCAGCCA

GSE1: chr16:85704776-85705193
ATAAGTTTCAGAGAGGAAATGTGCTCTTAAGAGATCAAAATTTGGCGGAT
CACGAGGTCAGGAGATCGAGACCATCCCGGCTAAAACGGTGAAACCCTGT
CTCTACTAAAAATACAAAAAATTAGCCGGGCGTAGTGGCGGGCGCCTGTA
GTCCCAGCTACTTGGGAGGCTGAGGCAGGAGAATGGCGTGAACCCGGGAG
GCGGAGCTTGCAGTGAGCCGAGATTGCGCCACTGCACTCCAGCCTGGGCG
ACAGAGCGAGACTCCGTCTCAAAAAAAAAAAAAAAAAGATCAAAATTTG
GACTGCATGCAATGGCTTACACCTGTAATCCCAGCACTTTGGGAGGCTGA
GGCAGGATCACATGAGCCCAGGAGTTCAAGGCTGCAGTGAGCTCTGACCA
CACCAGTGCACTCCAGCC

Supplementary Figure 7. Nucleotide sequences of ALCDs of PAX2, FAM460, KRT86, and GSE1.

DYM: chr18:46762580-46762997
GTGGCTCACACCTGTAATCCCAGCACCTTGAGAGGCTGAGATGGGCGGAT
CACCAGATCAGGAGATTGAGACCATCCTGGCTAACACGGTGAAACCCTGT
CTCTATTAAAAATACAAAAAATTAGCAGGGTGTGGTGGCAGGCGCCTGTA
GTCCTAGCTACTCGAGAGGCTGAGGCAGGAGAACGGTGTGAACCCAGGAG
GCGGAGCTTGCAGTGAGCTGAGATCGTGCCAATGCACTCCAGCCTGGGCG
ACAGAGCGAGACTCTGTCTCAAAAAAAAAAAATAAATAAATAAATAAATAG
CCATTCTCAGTCATGTTACTGGTACTTCAGAACTATTTGAATTTGATCAA
ATATACCCCACTTTAGGAAAACTAATATATGGAAAGAAGAAATTGCTTTA
TAAATGGCAAGATTCAGG

ARSG: chr17:66296989-66297406
CAAGCTTTAAAAAAAAAAAAAGCCACTTTGGGAGGCCAAGGTGGGTAGAT
CACGAAGTCAGGAGATCGAGACCATCCTGGCTAACATGGTGAAACCCCGT
CTCTACTAAAAATACAAAAAATTAGCCGGGCGTGGTGGCGGGCGCCTGTA
GTCCCAGCTACTCGGGAGGCTGAGGCAGGAGAATGGTGTGAACCTGGAAG
GCGGAGCTTGCAGTGAGCCGAGATCGCACCACTGCACTCCAGCCTGGGTG
ACAGAGTGAGACTCCGTCTCAAAAAAAAAAAAAAAGCATTTTAATTACGT
AAGTGAAATTGCTCTCCCAGGCTGGATAGAGGCATTCAAAGATGTCTTGT
GCATCTTCTGTTGAGCTTAGACTACTACATCTTGGCCCCTACACTCTAAG
AAAGCAGTCTTTCCAAGT

ACBD5: chr10:27505805-27506222
GTGGCTCACGCCTGTAATCTCAGCACTTTGGGAGGCAGAGGAGGGCAGAT
CACAAGGTCAGGAGATCGAGACCATCCTGGCTAACACGGTGAAACCCCGT
CTCACTAAAAAAATACAAAAAATTAGCTGGGCATGGTGGCGGGCACCTGTA
GTCCCAGCTACTCGGGAGGCTGAGGCAGGAGAATGGCGTGAAACCAGGAG
GCGGAGCTTGCAGTGAGCTGAGATCGCATCACTGCACTCCACCCTGGGCG
ACAGAGCGAGACTCTGTCTCAAAAAAAAAAAAAAAAAAGATGTTTCTCGG
CCGGGCGCGTTGCCTCACACCTGTAATTCCAGCACTTTGGGAGGCCAAGG
CGGGTGGATCACTTAAGGTCAGGAGTTTGAGACCACCCTGGCCAACATGG
TGAAACCCCGTCTCTACT

ATP2B2: chr3:10397489-10397906
GGCTCACGCCTGTAATCCCAGCACTTTGGGAGGCCGAGGCGGGCGGATCA
CGAGGTCAGGAGATCGAGACCATCCTGGCTAACACGGTGAAACCCCGTCT
CTACTAAAAAAATACAAAAAATTAGCCGGGCGTGGTAGCGGGCGCCTGTA
GTCCCAGCTACTCGGGAGGCTGAGGCAGGGGAATGGCGTGAACCCGGGAG
GCGGAGCTTGCAGTGAGCCGAGATCGCGCCACTGCACTCCAGCCTGGGCG
ACAGAGCGAGACTCCGTCTCAAAAAAAAAAAAAAAAAAAAAAAAAAAAAA
AAAAAAAAAAAAAAAAAAGAACTACTTGTCCTTTCCTTTGTGCGTGTGTG
CGTGTGTGTGTGTGTGTGTGTGTGTGTGTGTGTGGCAACCAGGCAAAGGC
CCTGAGGCAGGGAGGCAG

GSN: chr9:123965135-123965552
TGGCTCACGCCTGTAATCCCAGCACTTTGGGAGGCCACGACGGGCGGATC
ACGAGGTCAGGAGATCGAGACCATCTTGGCTAACACGGTGAAACCCCGTC
TCTACTAAAAATACAAAAAAATTAGCCGGGCGTGGTGGCGGGCGCCTGTA
GTCCCAGCTACTCGGGAGGCTGAGGCAAGAGAACGGCGTGAACCCGGGAG
GCGGAGCTTGCAGTGAGCCGAGATCGCGCCACTGCGCTCCAGCCTGGGCA
ACAGAGCGAGACTGTCTCAAAAAAAAAAAAAAAAAGGAACGTAAGCAAGGA
GGGCTGGTAGTCTGCGCCTGTAATCCTAGCTACTGGGGAGGCTGAGGAGA
GAGGATCGCTTGAGCCCAGGAGTTCTGGGCTGTAGTGCACTGTGTCACTA
GATAGAGTGTCTGCACTT

ANKH: chr5:14721769-14722186
GGTGGCTCATGCCTGTAATCCAGCACTTTGGGAGGCCAAGGTGGGAGGAT
CACAAGGTCAGGAGATCGAGACCATCCTGGCTAACACGGTGAAACCCCAT
CTCTACTAAAAATACAAAAAATTAGCCAGGCATGGTGGCAGGCGCCTGTA
GTCCCAGCTACTCGTGAGGCTGAGGCAGGAGAATGGCGTGAATCGGGGAG
GCGGAGCTTGCAGTGAGCTGAGATCATGCCACTGCACTCCAGCCTGGGCG
ACAGAGCGAGACTCCGTCTCAAAAAAAAAAAAAAAAAAAAAAAAAGTGGG
TATGGTATGTATTAAAAGCTACAATACCTATCTGCAGGTGAGGTTTCCCT
AGCTTCCCCTAGGCAAAGTACTGAGCCCTAGCATAATAATCAGACTACAT
TTAACAAGTCCATTTGAA

Supplementary Figure 8. Nucleotide sequences of ALCDs DYM, ARSG, ACBD5, ATP2B2, GSN, and ANKH.

GABRA5: chr15:27130176-27130593
GCTCACGCCTGTAATCCCAGCACTTTGGGAGGCCGAGGCGGGAGGATCAC
GAGATCAGGAGATCGAGACCATCCTGGCTAACTTGGTGAAACCCCGTCTC
TACTAAAAAAAATACAAAAAATTAGCCGGGCGTGGTGGCGGATGCCTGCA
GTCCCAGCTACTTGGGAGGCTGAGGCAGGAAAATGGCGTGAACCTGGGAG
GCGGAGCTTGCAGTGAGCCCAGATCGCACCACTGCACTCCAGCCTGGGCA
ACAGAGCAAGACTCCGTTTCAAAAAAAAAAAAAAAAGAATGTGGTGACT
CAGGCAAGAGATGTGGGGGGCGTGAGCTCTATCCTGTTTGAGCTTCAAAG
CATCTTTCTGGAGACACAATCAGGGACTTTGTGAAATGATGCTGGCAGGT
GGAAATGAGCAAAGGTGG

TRIM16L: chr17:18637497-18637914
GTGGCTCACGCCTGTAATCCCAGCACTTTGGGAGGCCGAGGCGGGTGGAT
CATGAGGTCAGGAGATCGAGACCATCCTGGCTAACAAGGTGAAACCCCGT
CTCTACTAAAAATACAAAAAATTAGCCGGGCGCGGTGGCGGGCGCCTGTA
GTCCCAGCTACTCGGGAGGCTGAGGCAGGAGAATGGCGTGAACCCGGGAA
GCGGAGCTTGCAGTGAGCCGAGATTGCGCCACTGCAGTCCGCAGTCCGGC
CTGGGCAACAGAGCGAGACTCCGTCTCAAAAAAAAAAAAAAAAATGAAT
GAATGGATCAAATGTGGGTATAGGAGTACCTGCTTCCCCAAACACTTGCC
AACACTGGATAGGATAATGCCAGGCATTCAATACGTGTTTGTTGAATGAA
TGAATGAATGCATGAATG

LINC02242: chr5:66928100-66928517
GTGGCTCACGCCTGTAATCCCAGCACTTTGGGAGGCCGAGGCGGGCGGAT
CACGAGGTCAGGAGATCGAGACCATCCCGGCTAAAACGGTGAAACCCCGT
CTCTACTAAAAATACAAAAAATTAGCCGGGCGTAGTGGCGGGCGCCTGTA
GTCCCAGCTACTTGGGAGGCTGAGGCAGGAGAATGGCGTGAACCCGGGAG
GCGGAGCTTGCAGTGAGCCGAGATCCCGCCACTGCACTCTAGCCTGGGCG
ACAGAGCGAGACTCTGTCTCAAAAAAAAAAAAAAAAGTTGGCCTAAACA
GGGACACACAGTGTGGTCCAGAGACCTGGTGACAGTGTGTGAATTGTTAC
CAGTTCACAGAGCAGTAAGTATTACAAAACTTGGGAGTAGGTCATTAGAA
ATTTTATAGGGATTTCAG

YEATS2: chr3:183446790-183447207
GTGGCTCACACCTATAATCCAAGCACTTTGGGAGGCCAAGGCAGGCGGAT
CATGAGGTCAGGAGATTGAGACCATCCTGGCTAACACGGTGAAACCCGGT
CTCCACTAAAGATACAAAAAATTAGCTGGGCGTGGTGGCGGGCGCCTGTA
GTCCCAGCCACTAGGGAGGCTGAGGCAGGAGAATGGCGTGAACCAGGGAG
GCGGAGCTTGCAGTGAGCTGAGATCGCGCCACTGCACTCCAGCCTGGGCG
ACAGAGCGAGACTCTGTCTCAAAAAAAAAAAAAAGGTAGTTGTAGTTCA
CTCTCTCAGATTACTGTCTCACTGAGCAGGCATTGTCTTCTATCCAATTG
TTACCACACTAATTATATTAACTGGTTTTGCTCCCCTCTGGAAACAGCTA
AACTTGTTTATATAGATA

LRRC37A3: chr17:62913094-62913511
TGGCTCACGTCTGTAATCCCAGCACTTTGGGAGGCCGAGGCGGGCAGATC
ACGAGGTCAGGATATCGAGACCATCCTCGCTAACATGGTGAAACCCCGTC
TCTACTAAATATACACAAAAATTAGCCGGGCGTGGTGGTAGGCCCCTGTA
GTCCCAGCTACACGGGAGGCTGAGGCAGGAGAATGGCATGAACCCGGGAG
GCGGAGCTTGCAGTGAGCCGAGATCGCGCCACTGCACTCCAGCCTGGGTA
ACAGAGCCAGACTCCATCTCAAAAAAAAAAAAAAAAAAAAAAAACCTGA
AAATATCAAGTGCTAGGGAGGATACAGAGCAACCGGAACTCTTCATACAT
TTGCAGGTAAGAATGCAAAATGGTACAGCTACTTTGGAAAACAGTTTTGG
CAGTTTCTTATAAACATA

MAPKBP1: chr15:42074190-42074607
TAATCCTAGCATTTTGGGAGGCCGAGGCGGGCGGATCATGAGGTCAGGAG
ATCGAGACCATCCTGGCTAACACGGTGAAACCCTGTCTCTACTAAAAATA
CAAAAAAAAAAAAAAAAAAATTAGCTGGGCGTGGTGGCGGGCGCCTGTA
GGCCCAGCTACTCGCGAGGCTGAGGCAGGAGAATGGTGTGAACTCGGGAT
GCGGAGCTTGCAGTGAGCTGAGATCGCGCCACTGCCCTCCAGCCTGGGAG
ATAGCGAGGCTCTGTCTCAAAAAAAAAAAAAAAAATTATGAAATACAAC
AGACATTAAGAGAGTGTACAAATATAATATTGTATTATAAATAATGATAA
ATGATTTCCCCGTGTAACCACTGCCCAAGTGAAAAAATAGAACAATGGTA
AGGTATTGGCAGTTTCTG

Supplementary Figure 9. Nucleotide sequences of ALCDs of GABRA5, TRIM16L, LINC02242, YEATS2, LRRC37A3, and MAPKBP1.

RPS29: chr14:50041629-50042046
GCTTACGCCTGTAATCCTAGCAGTTTGGGAGGCTGAGGCGGGTGGATCAC
CTGAGGTCAGGAGTTAAAGACCAGCCTGGCCAATGCAGTGAAACCCCATC
TTTACTAAAAATACAAAAAAATTAGCCAGGCATGGTGGCGAGTGCCTGTA
ATCTCAGCTACTCAGGAGGCTGAGGCAGGAGAATGGCGTGAACCCGGGAG
GCGGAGCTTGCAGTGAGCGGAGATCAGGCCGCTGCACTCCAGCCTGGGCG
ACAGAGCGAGACTCCGTCTCAAAAAAAAAGATAAAGAAAGAGATCCCTGA
CTGCCTGTATAGTTTGAGAACCACTGATCCTGGGGCAAGGACTCAAAGTC
TCTGGCTCTAGAAGTGGCTGGTGTGGACCTCAAATGGACCCTGTTCTAAG
AGTCCAAAGGACACATTT

TM4SH19: chr3:196052716-196053133
TGGCTCACGCCTGTAATCCCAGCACTTTGGGAGGCCAAGATGGGCGGATC
ACCTGAGGTCAGGAGTTTGAGACCAGCCTGGCCAACATGGTGAAACCCCG
TCTCTACCAAAAATACAAAAATTAGCTGGGCGTGGTGGTGGGCGCCTGTA
ATCCCAGCAACTCAGGAGGCTGAGGCAGGAAAATCTCTTGAACCCGGGAG
GCGGAGCTTGCAGTGAGCCGAGATGGCGCCACTGCACTCCAGCCTGGGCG
ACAGAGCGAGACTCCGTTTCAAAATAAATAAATAAATAAGTACATAGATA
AATAAATAAACACTTGCACATTTCATTCACTGTCTGTTCTCATTCTGGTT
TTCCCCGTGGCACGTAGTTCTGACACTTGAATGTGCATGAGAATCACCTG
GAGGGCTTTTGCCGGAAC

MAST1: chr19:12959171-12959588
GGCCTGTAATCCCAGCACTTTGGGAGGCCGAGGGGGGCGGATCACGAGGT
CAAGAGATCGAGACCATCCTGGCTAACACGGTGACACCCCGTCTCTACTA
AAAACACACACACACACAAAATTAGCCGGGCGTGGTGGCGGACGCCTGTA
GTCCCAGCTACTCGGGAGGCTGAGGCAGGAGAATGGCGTAAACCCGGGAG
GCGGAGCTTGCAGTGAGCCGAGATTGCGCCGCTGTACTCCAGCCTGGGCG
ACAGAGCGAGACTCCGTCTCAAAAAATAATAATAGTAAATAAAATAAAAT
AAAATAAAATATTAAACTTAGCCGGGTGCGGTGGCTCAGGCCTGTAATCC
CAGCACTCTGGGAGGCTGAGGCAAGTGGATCACCTGAGGTCAGGAGTTCG
AGACCAGCCTGGCCAACA

PALM2-AKAP2: chr9:112686610-112687027
AGATGGTGCCTGTAATCCCAGCACTTTGGGAGGCCGGGGCGGGTGGATCA
CGAGGTCAGGAGATCGAGACCATCCTGGCTAACGTGGTGAAACCCTGTCT
GTACTAAAAAATACAAAAAAATTAGCCAGGTGCGGTGGCAGGCGCCTGTA
ATCCCAGCTACTGAGGAGGCTGAGGCAGAAGAATGGCATAAACCTGGGAG
GCGGAGCTTGCAGTGAGCTGAGATTACGCCACTGCACTCCAGCCTGGGCG
ACACAGCAAGACTGCGTCTCAAAAAAACAAAAACAAAAAACAAAGACAAA
CAAACAAAAAAACAGGTGGTGCCGTGTATCAAAGAGTAGAGTCCGTAAAA
CCAGTAGGCTCTGAACAAACATCTCAGGACCTCCCTCCATTTTATAACTG
TTTCTGCTGTACCTATGA

FAM120A02: chr9:96210770-96211187
CACGCCTGTAATCCCAGCACTTTGGGAGGCTGAGGTGGGCGGATCACAAG
GTCAGGAAATCCAGACCATCCTGGCTAACACGGTGAAACTCCGTCTCTAC
TAAAAAAAATACAAAAAAAAATTAGCCGGGCGTGGTGGCAGGCGCCTATA
GTCCCAGCTACTCAGGAGGCTGAGGCAGGAGAATGGCGTGAACCCAGGAG
GCGGAGCTTGCAGTGAGCCGAGATCACGCCACTGCATTCTAGCCTGGGCA
ACAGAGCGAGACTCCATCTTAAAAATAAAAAATAAAAAATAAACATCTGT
ATTTAAAATTTAATAAAAAGTTATGTCTTCTTGGAGGTTACAATATGACA
ACTAATGGTTGGGGACTTTTTCTTTTTTTATTTTGTATTTATTTTATTTA
TTTTTTCTCCCCCAAGAT

GSS: chr20:33541289-33541706
TGGGCGGGGTGGCTCACGCCTGTAATCCCAGCACTTTGGGAGGCCGAGAG
TGGGGGATCACGAGATCGAGACCATCCTGGCTAACACGGTGAAACCCCGT
CTCTACTAAAAATACAAAAAATTAGCCGGGCATGGTGGCAGGTGCCTGTA
GTCCCAGCTACTCGGGAGGCTGAGGCAGGAGAATGGCATGAACCCAGGAG
GCGGAGCTTGCAGTGAGCCGAGATCGTGCGGCTGCACTCCAGCCTGGGCG
ACAGAGCCAGACTCCGTCAAAAAAAAAAAAAAGAAAGAAAGAGGGAGGG
AGAGAGAGAGAAAGAAAGAAAAAGAAGGAAGGAAGGAAGGAAAGAAGGAG
GGAAGGGAAAGGGAAAGGGAAAAGGAAGGAAGGAAAGAAGGGAAAGGGAG
AAGTCGTATTATTATGGA

Supplementary Figure 10. Nucleotide sequences of ALCDs of RPS29, TMSH19, MAST1, PALM2-AKAP2, FAM120A02, and GSS.

C11orf65: chr11:108256139-108256556
GTGGCTCTCGCCTGTAATCCCAGCACTTTGGGAGGCCGAGGTAGTTGGAT
CACAAGGTCAGCAGATCAAGACCATCCCGGCTAACACAGTGAAACCCTGT
CTCTACTAAAAATACAAAAAATTAGCGGGGTGTGGTGGCGGGTGCCTGTA
GTTCCAGCTACTTGGGAGGCTCAGGCAGGAGAATGGTGTGAACCTGGGAG
GCGGAGCTTGCAGTGAGCCAACATCGCACCACTGCACTCCAGCCTGGGCG
ACAGAGCAAGACTCCGTCTCAAAAAAAAAAAAAAGATTCATATTTTTAAAA
TCACTTTATTCAAACTTAAATAATATAGGACATTTCCTTCTATTCCCAAA
TAACAATCTTACATACAAAGGTAAAAATGACTTGACAAATGCATCTAAAT
TTATATACTATGCAGATT

NLRP11: chr19:56305856-56306273
GTGGCTCACATCTGTAATCCCAGCACCTTGGGAGGCTGAAGTGGGTGGAT
CACGAGGTCAGGAGATCGAGACCATCCTGGCTGACACGGTGAAACCCCGT
CTCTACTAAAAATACAAAAAATTAGCCAGGCGTGGTGGCGGGTGCCTGTG
GTCCCAGCTACTCGGGAGGCTGAGGCAGGAGAATGGCGTGAACCCGGGAG
GCGGAGCTTGCAGTGAGCCGAGATCGCGCCACCGCACTCCAGCCTGGGCA
ATACAGCGAGACTCCGTCTCAAAAAAAAAAAAATAAATAAAAGCATACATA
ATGCGATATTACATAATATGAACAGGAGTTTCTGGTGTTATCCTGCAATC
AAACAAGTATTTTTTACAGAAAATATAAAGAATATAAAGAATATTATATC
AGTTTGTATTATGTTTTG

SIDT1: chr3:113287171-113287588
GGTGGCTCACTCCTGTAATCCTAGCACTTTGGGAGGCCGAGGAGGTGGAT
CACGAGGTCAGGAGATCGAGACCATCCTGGCTAACACAGTGAAACCCCAT
CTCTACTAAAAATACAAAAAATTAGCCAGGCACGGTGGCGGGCACCTGTA
ATCCCAGCTACTCGGGAGGCTGAGGCAGGAGAATGGCATGAACCTGGGAG
GCGGAGCTTGCAGTGAGCCGAGATCGCGCCACTGCACCCCAGCCTGGGCA
ACACAGCAAGACTCTGTCTCAAAAAAAAAAAAGAAAAGAAAAGAAAAGAA
AAGAAAAAAAAACAGAAATGTAAATTAAAACCATAATGTGATTCCATTACA
ACTACCAAATTGCTATAATTTGAAAGGCCTACATTCCACTCCTTTGATGT
ATACCCAACTCCTAGATA

TSPAN14: chr10:82252302-82252719
GTGGCTCACATCTGTAATCCCAGCACTTTGGGAGGCCGAGGCAGGCGGAT
CACGAGGTCAGGAGATCAAGACCATCCTGGCTAACACGGTGAAACTCCGT
CTCTACTAAAAATACAAAAAATTAGCCGGGCGTGGTGGTGGGCACCTGTA
GCCCCAGCTACTCAGGAGGCTGAGGCAGGAGAATGGCGTGAACCCGGGAG
GCGGAGCTTGCAGTGAGCCAAGATCGCGCCACTGCACTCCAGCCTGGGCG
ACAGAGCGAGACTCAGTCTCAAAAAAAAAAAAGACTTATTTTCACAATAG
CATTCTTTATAGATAAATACCGTTTGGTACCTAATAGATTTTTTTAAGCT
CTCTTTCTGACTTTGGGGCCAAGGTTTTCCCGGGATTGTTAAAACCTGCT
TGGGATAAGAAATGTGGA

GCLC: chr6:53413601-53414018
TGTTTTCACCAATTAAGAAATTTAACATTGGGAGGCCGAGGCGGGCGGAT
CACGAGGTCAGGAGATCGAGACCATCCCGGCTAAAACGGTAAAACCCCGT
CTCTACTAAAAATACAAAAAATTAGCCGGGCATAGTGGCGGGCGCCTGTA
GTCCCAGCTACTTGGGAGGCTGAGGCAGGAGAATGGCGTGAACCCGGGAG
GCGGAGCTTGCAGTGAGCCGAGATCCCGCCACTGCACTCCAGCCTGGGCG
ACAGAGCGAGACTCCATCTCAAAAAAAAAAAAAAAAGAAAGAAATTTAAC
AGACAAAAAGCTTAAGGACTTCACAGGCAGATCCGCGGGTTTTGGGGAAA
GGGTGTGTCAAGGGAGAAGAAAGGATGTAATGAAGGTTGGCATCAAGTCC
CAGGAAGAATCACACTGA

Supplementary Figure 11. Nucleotide sequences of ALCDs of C11orf65, NLRP11, SIDT1, TSPAN14 and GCLC.

CYP2C8: chr10:96801268-96801685
GTGGCTCACGCCTGTAGTCCCAGCACTTTGGGAGGCCGAGGCGGGTGGAT
CATGAGGTCAGGAGATCGAGACCATCCTGGCTAACAAGGTGAAACCCCGT
CTCTACTAAAAATACAAAAAATTAGCCGGGCGCGGTGGCGGGCGCCTGTA
GTCCCAGCTACTCGGGAGGCTGAGGCAGGAGAATGGCGTGAACCCGGGAA
GCGGAGCTTGCAGTGAGCCGAGATTGCGCCACTGCAGTCCGCAGTCCAGC
CTGGGCGACAGAGCGAGACTCCGTCTCAA**AAAAAAAAAAAAAAAAAAAAA**
AAAAATCTGTGCAAAACTACCAAGGTGCTCTGGGCTCTGCTCCCTGCCAT
CCCCAGTTTGCTAGCCTTATTTAGGGAAGGTACAGGAGTCTGATCACTTG
GAATGACATGAGCCTTCT

SLC10A1: chr14:70255132-70255549
GGCTCACGCCTGTAATCCCAGCACTTTGGGAGGCCCAGGCGGGTGGATCA
CGAGGTCAGGAGATCGAGACTATCCTGGCTAACACGGTGAAACCCCATCT
CTACTAAAAATACAAAAAAAAATTAGCCGGGCGTGGTGGCAGGCGCCTGTA
GTCCCAGCTACTCGGGAGGCTGAGACAGAAGAATAGCGTGAACCCAGGAG
GCGGAGCTTGCAGTGAGCCGAGACTGCGCCACTGCACTCCAGCCTGGGCG
ACAGAGCAAGACTCCATCT**CAAAAAAAAAAAAAAAAGAG**TTCTTCATATAT
ATTCTGAATATTAGACCCTTATCAGATATATGACTTGCAAATATTTCCTC
CCATTCTCTTGTTGGCTTTTCACTTTCTTGATGGTGTCCTTTGAATCACA
AGTTTTTAATATTAATGA

PON1: chr7:94931139-94931556
CCTGTAATCCCGGCACTTTGGGAGGCCGAGGCGGGTGGATCACAAGGTCA
GCAGATCAAGACCATCCTGGCCAACAGGATGAAACCCTGT CTCTACTAAA
AAAAAAAAAAAAATACAAAAAATTAGCCAGGCATGGTGGCATGCGCCTGTG
GTCCCAACTACTTGGGAGGCTGAGGCAGGAGAATGGTGTGAACCCGGGAG
GCGGAGCTTGCAGTGAGCCAAGATTGCGCCACTGCACTCCAGCCTGGGCG
ACAGAGCGATACTCTGTCAC**AAAAAAAAAAAAAAAAAAAAAAAAA**CCAAG
AATTGAGAATTATGTTGTCAACTGAAAAAACAGTAGCTGGGAATAAAGTC
ACTTATCTGGTTCATTTTTAAAACTTACCTCTGATGCAGGAGGATTCTCT
GAGTCATAGAAGAAGATT

p21: chr6:36649240-36649657
GTCTCACACCTGTAATCCCAGCACTTTGGGAGGCCGAGGAGGGCGGAGCA
CGAGGTCAGGAGATTGAGACCATCCTGGCTAACACGGTGAAACCCCGTCT
CTACTAAAAATACAAAAAAAAATTAGCCGGGCATGATGGCAGCACCTGTA
GTCCCAGCTACTCGGGAGGCTGAGGCAGGAGAATGGCGTGAACCCGGGAG
GCGGAGCTTGCAGTGAGCCGAGATCACGCCACTGCACTCCAGCCTGGGTG
ACAGAGCGAGACCGCATCTC**AAAAAAAAAAAAAAAAAAAAAAAAAAAT**CTGG
GCATGGGGGCGGGGGGATCACGCCTGTGGTCCCGGTTACTTGGGAGGCTG
AAACAGGAGGACTACTTGAGTCCAGGATGGTGCCATTGCACTCCAGCCTG
GGTGACAGAGTGCAACTC

TD02: chr4:156837190-156837607
TTTTAGACGCTTGTAATCCCAGCACTTTGGGAGGCCGAGGCGGGCCGATC
ACGAGGTCAGGAGATCGAGACCATCCTGGCTAACACGGTGAAACCCCGTC
TCTACTAAAAATACAAAAAAATTAGCCTGGCGTGGTGGTGGGCGCCTGTA
GTCCCAGCTACTCGGGAGGCTGAGGCAGGAGAGTGGCGTGAACCCGGGAG
GCGGAGCTTGCAGTGAGCCGAGATTGCACCACTGCACTCCCGCCTGGGCC
ACAGAGCGAGACTCCGTCTC**AAAAAAAAAAAAAAAAAAAAAG**GATATTTG
ATTAATTTGTGTAGTGTTTAACCCCTTTTAACCCTGTTCAGGGAAAAAGT
TATGCCTTTACTATTTAAAAGAAAAAAAAAGGACGGGGGCATAAAACTT
CATTACTAATTATCTTCC

Supplementary Figure 12. Nucleotide sequences of 250bp inactive domain CYP2C8, SLC10A1, PON1, p21 and TD02.

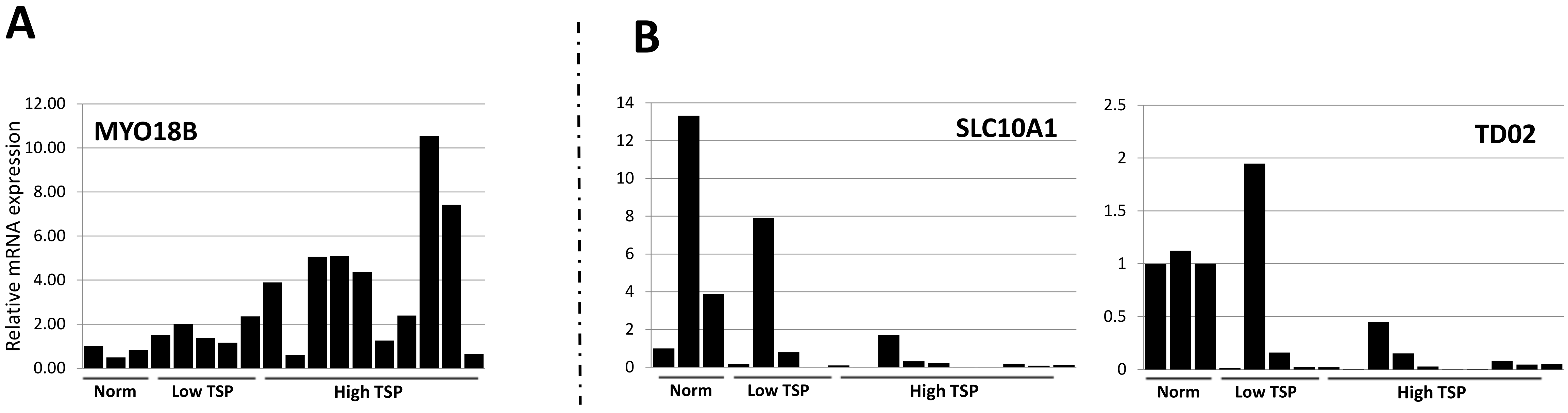

Supplementary Figure 13. (**A**) Expression of additional representative cancer related genes with active ALCDs in HBL with low and high levels of TSPs. **(B)** Expression of additional representative genes with inactive 250bp domains in HBL with low and high levels of TSPs.

1) X-ray repair cross-complementing protein 6 isoform 1
2) X-ray repair cross-complementing protein 6
3) 60kD heat shock protein, mitochondrial
4) Cytoskeleton associated protein 4
5) Heat shock protein HSP90
6) T-complex protein 1 subunit theta
7) Insulin-like growth factor 2 mRNA binding protein
8) Heterogeneous nuclear ribonucleoprotein U (Matrix Attached Protein,)
9) Elongation factor 1-alpha
10) Nucleolin OS
11) 26S proteasome non-ATPase regulatory subunit 3
12) High mobility group protein B1

Supplementary Figure 14. List of proteins determined in fourth “diffused band” that interact with 18BP core sequence of ALCDs.

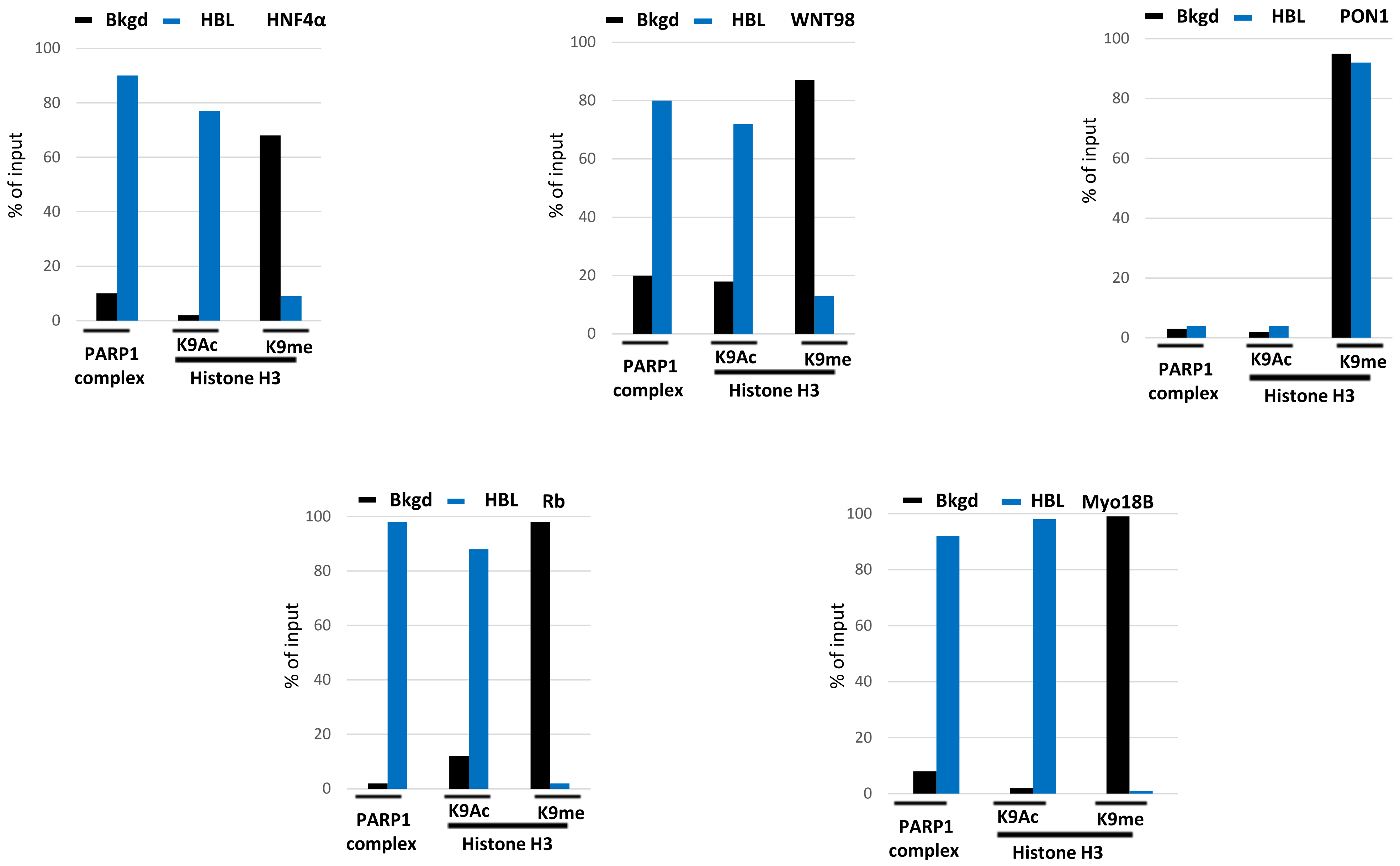

Supplementary Figure 15. Quantitative ChIP for Fig 6b.

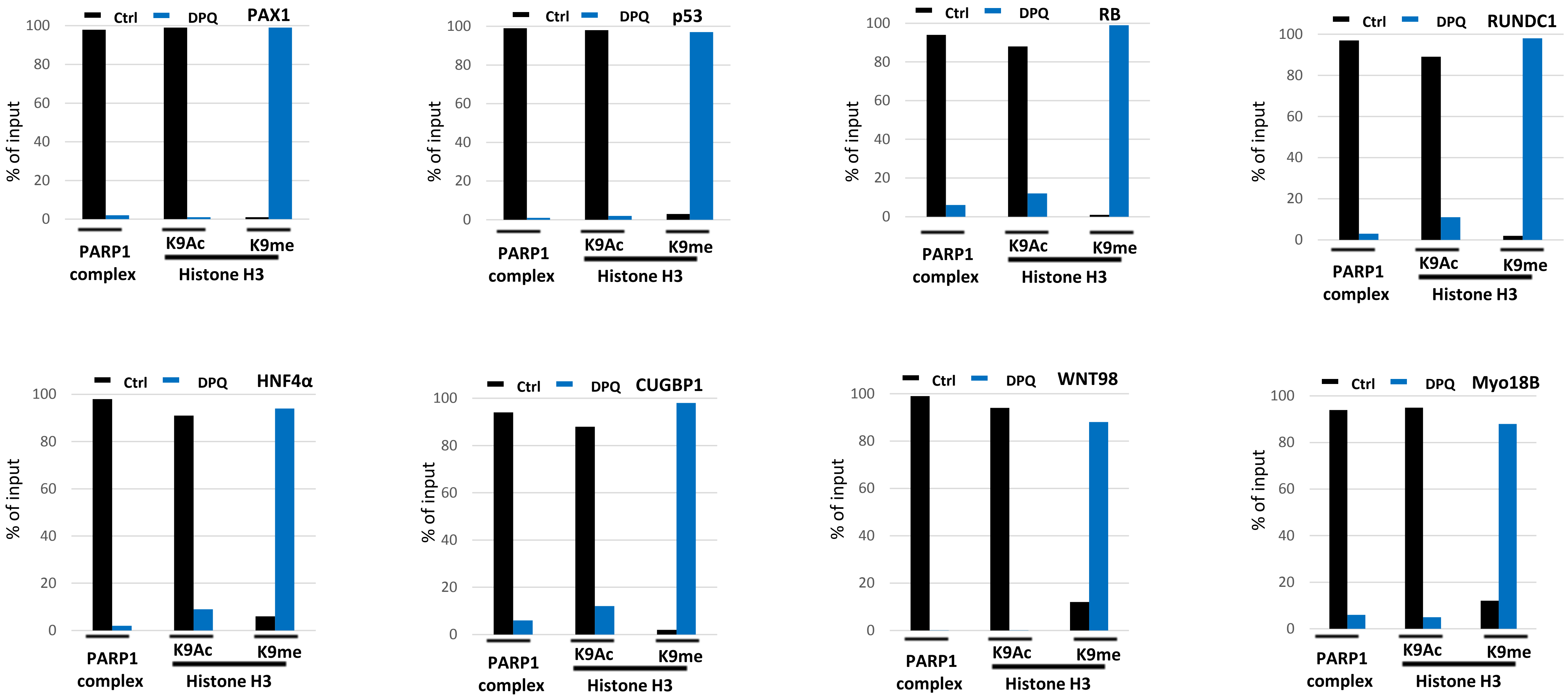

Supplementary Figure 16. Quantitative ChIP for Fig 6d.

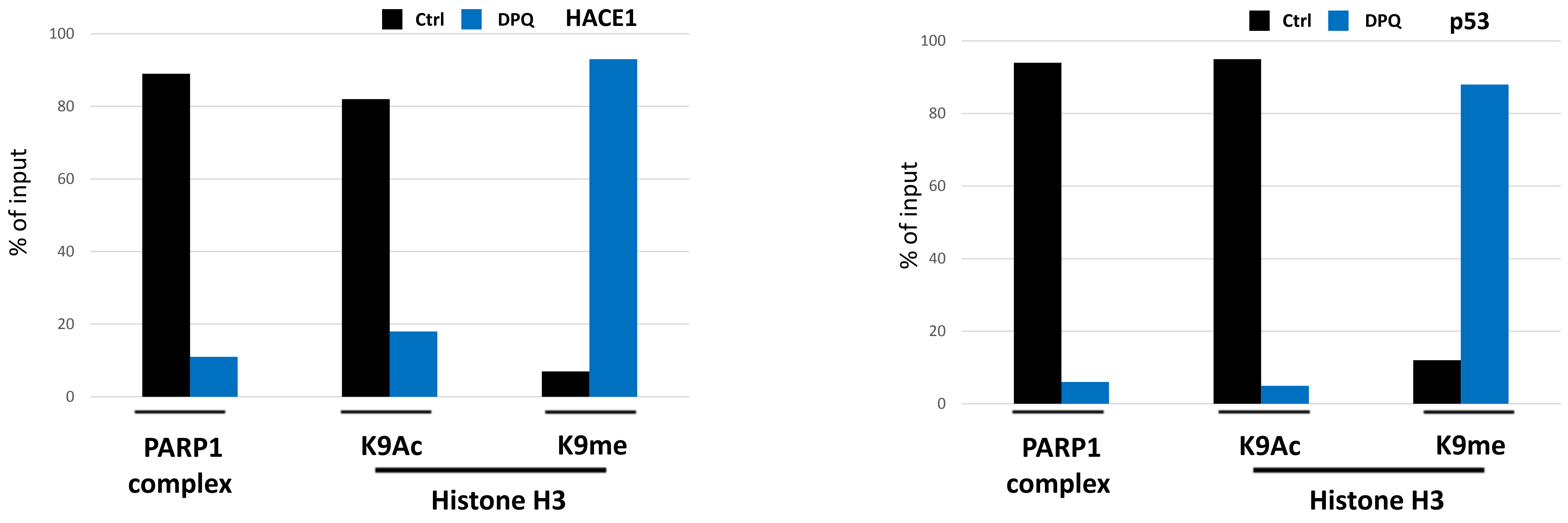

Supplementary Figure 17. Quantitative ChIP for Fig 7g.

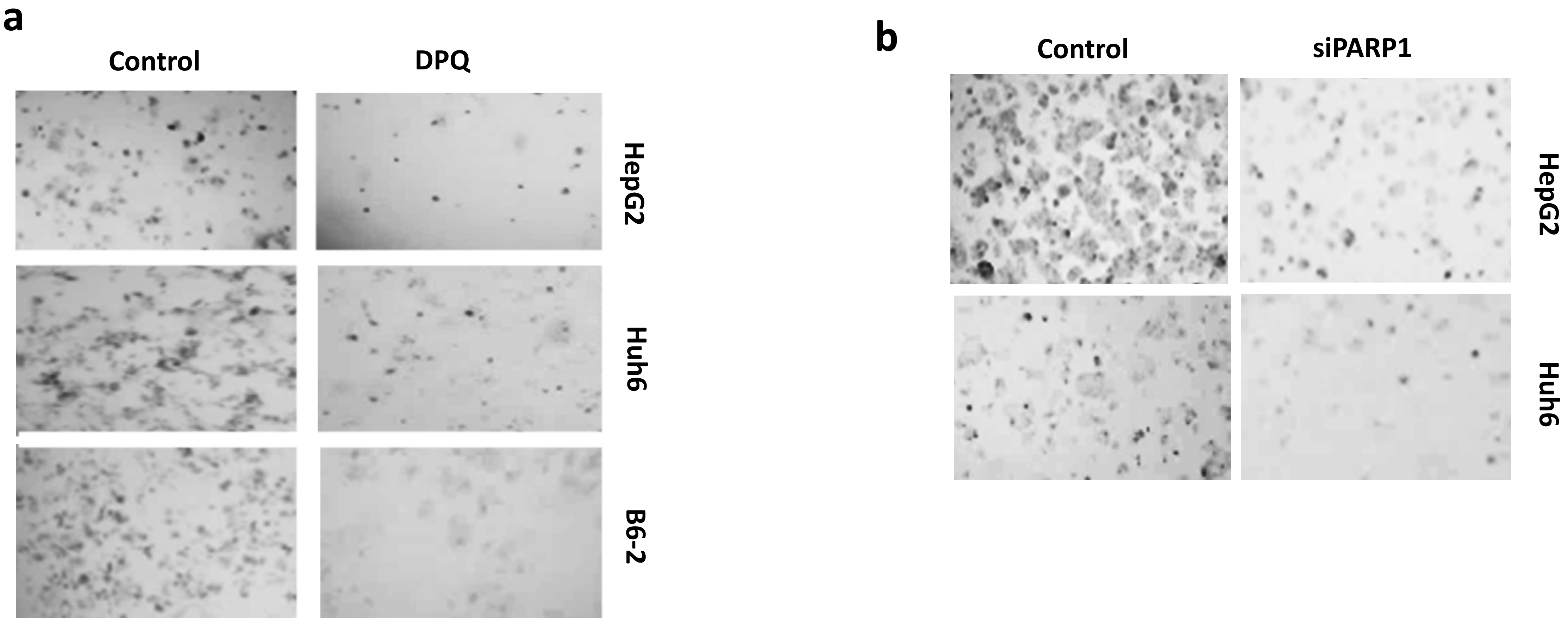

Supplementary Figure 18. **(a)** Representative images of cells with decrease in cell proliferation: supplements Fig 6e. **(b)** Representative images of cells with decrease in cell proliferation: supplements Fig 7d.

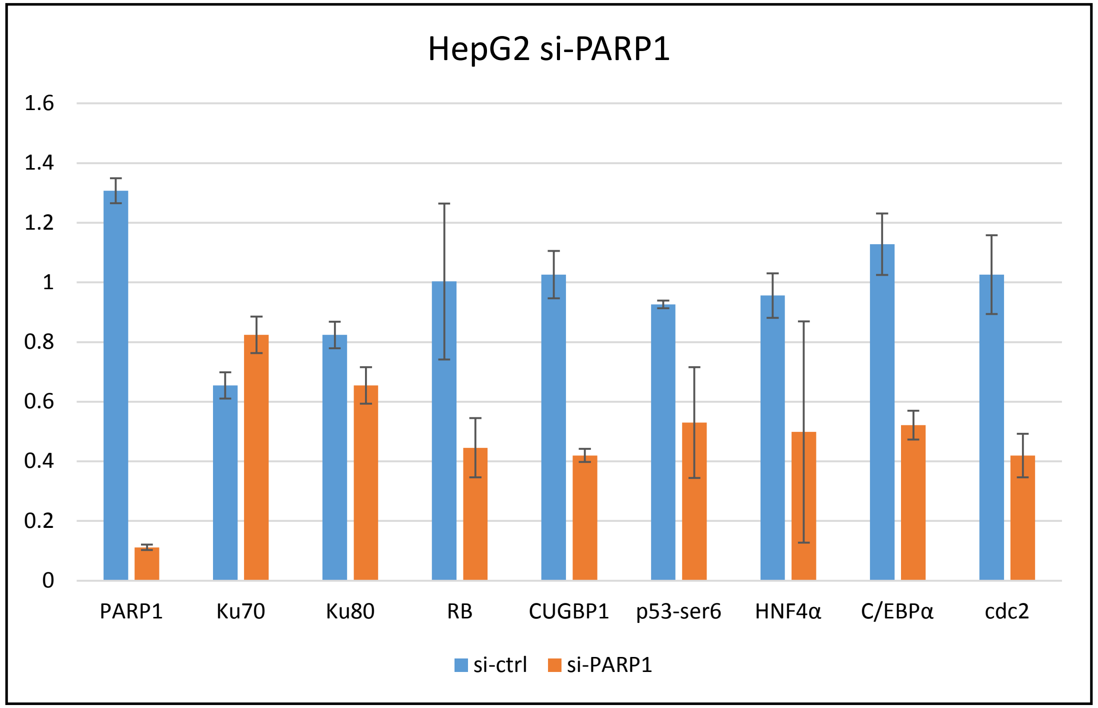

Supplementary Figure 19. Calculations of levels of proteins as ratios to β-actin in si-PARP1 treated HepG2 cells.

	Forward	Reverse
**CUGBP1**	CACAAGGTCAGGAGATCGAG	ATCTCAAAAAAAAAAAAATATATATATACACAC
**p53**	GAGGTCAGGAGATCGAGACC	ATACCTAATGAGAGAGGTTGCGATA
**HNF4α**	ACAAGGTCAGGAGATCGAGA	TGACTGCAGTGACCAAAGGG
**C/EBPα**	CAAGACCAAGACTCGCCCTC	GCCTGCCGGGTATAAAAGCT
**RB**	ACGAGGTCAGGAGATCGA	CCAGAAATAAACCCTGGTTTTT
**HACE1**	ACGAGGTCAGGAGATCGA	GTAATGTGGTAAGAACTCAGTTAGCTGA
**RUNDC1**	CGAGGTCAGGAGATTGACCA	GATGTACGATAGACCACTTCTGAA
**MYO18B**	CACGAGGTCAGGAGATCG	ATAAGTAGGTGAATGAATGAACAGTTAG
**PGAP1**	CACAACATCAGGAGATCGAG	GAGATCTTAACTGCAACTTTTAAGG
**PON1**	CCGGTTGTCCTACTTTGG	GACTTTATTCCCAGCTACTGTTTTT
**p21**	GCTCCAGTCCTCTAACTCTGG	CAGCCTCCCAAGTAACCG
**β-catenin**	ATTAAGGTCGTGAAACCCT	CATGTTATGGCTCTGCTTACC

Supplementary Figure 20. Nucleotide sequences of ChIP primers.
Note that the search for unique Forward primers was complicated by highly homological sequences in the 5’-region of 250bp domains. Therefore, some forward primers have a certain level of homology; however, reverse primers are quite different and the PCR reaction provided unique and specific products.

Supplementary Fig 21. Full-length immunoblots corresponding to the blots in Figure 1b.

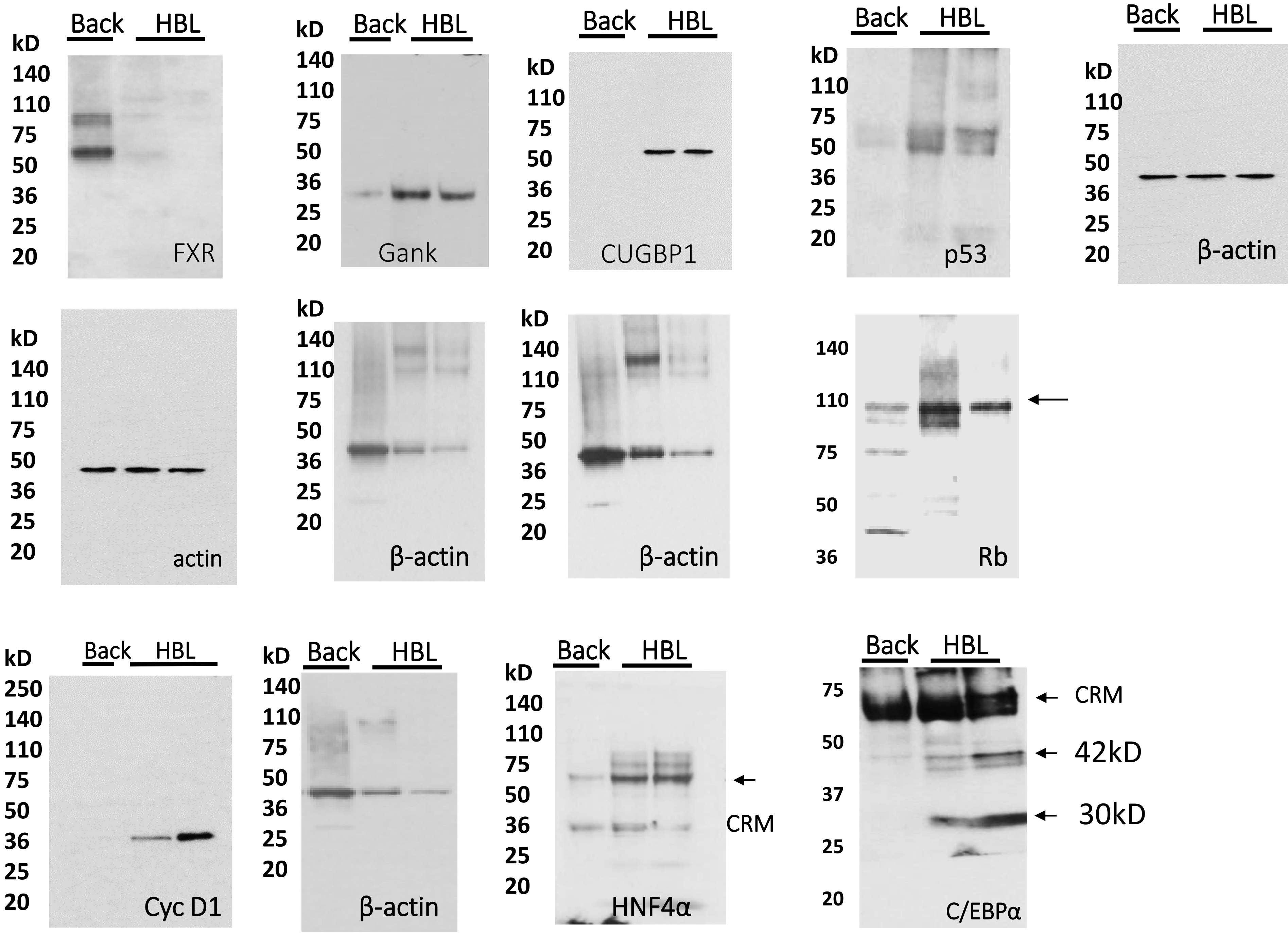

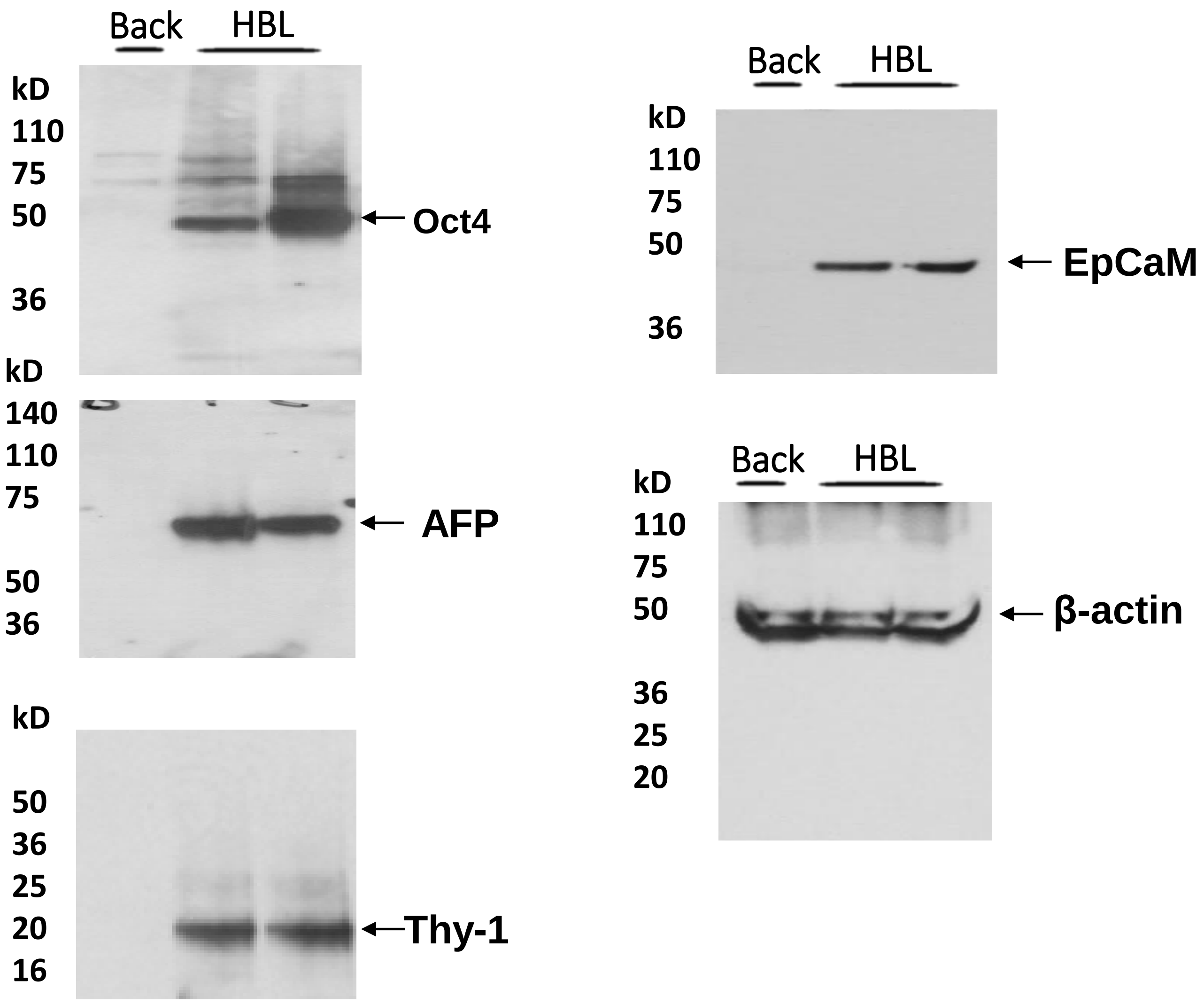

Supplementary Fig 22. Full-length immunoblots corresponding to the blots in Figure 1e.

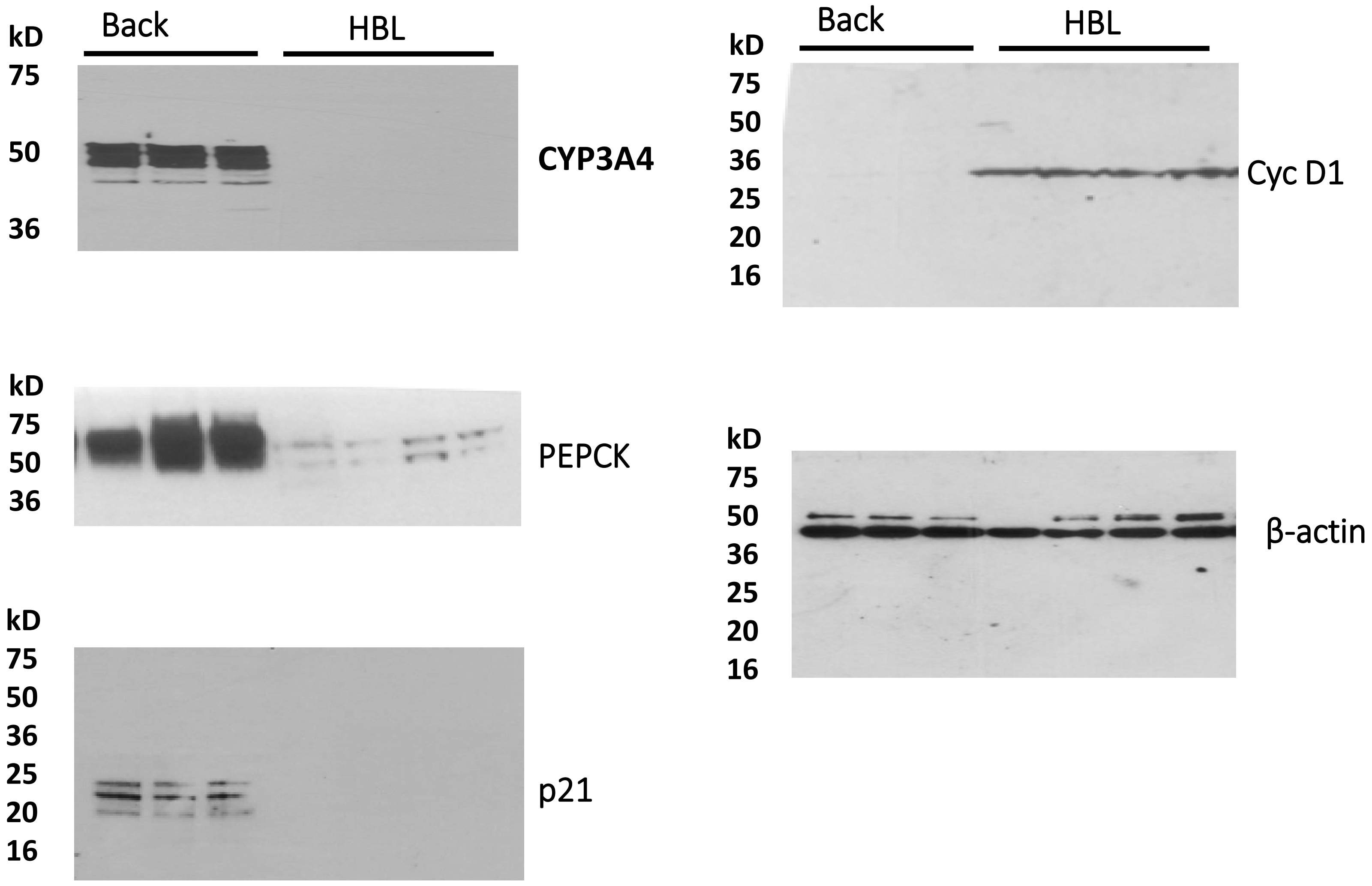

Supplementary Fig 23. Full-length immunoblots corresponding to the blots in Figure 1g.

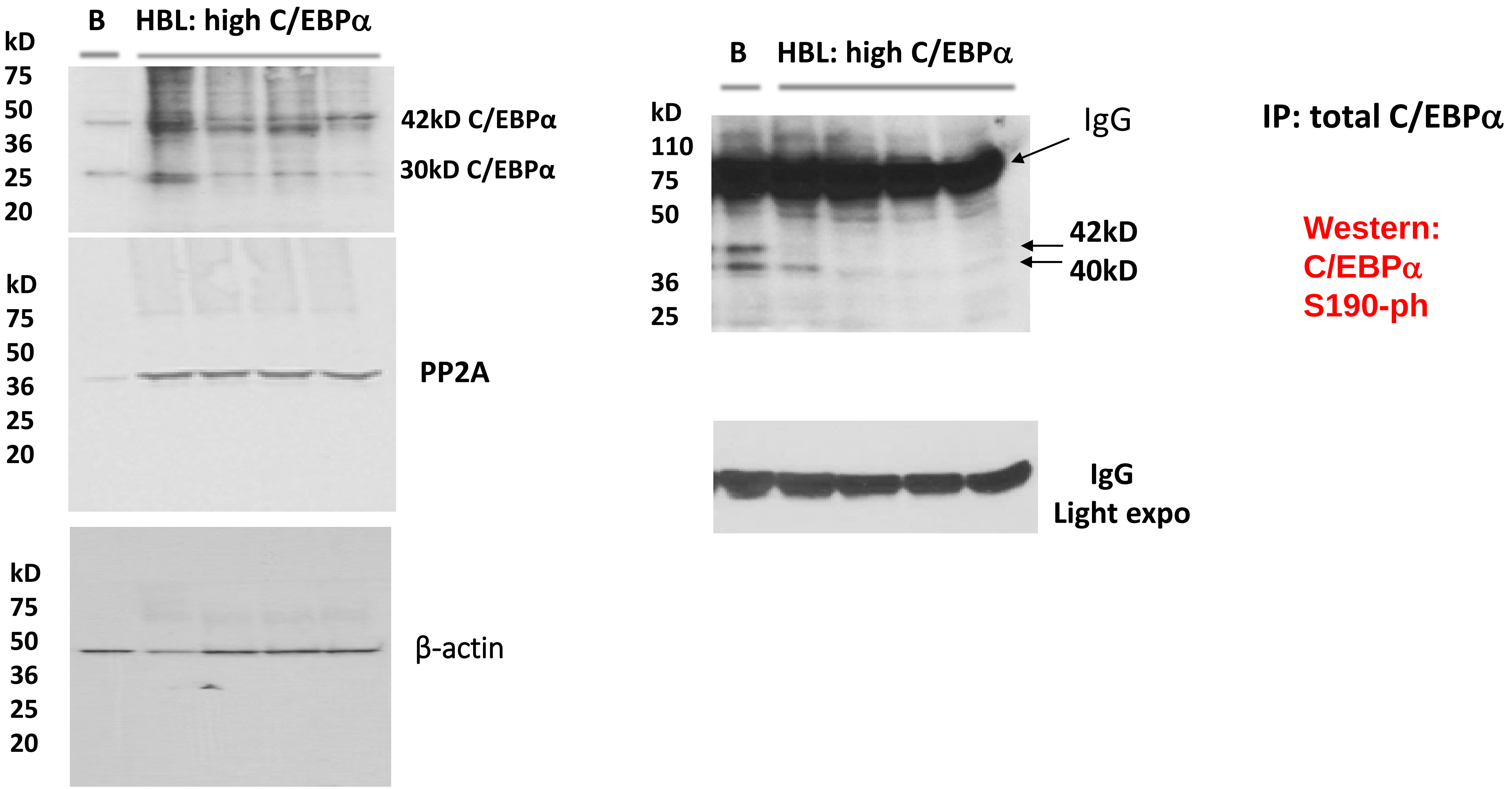

Supplementary Fig 24. Full-length immunoblots corresponding to the blots in Figure 2a.

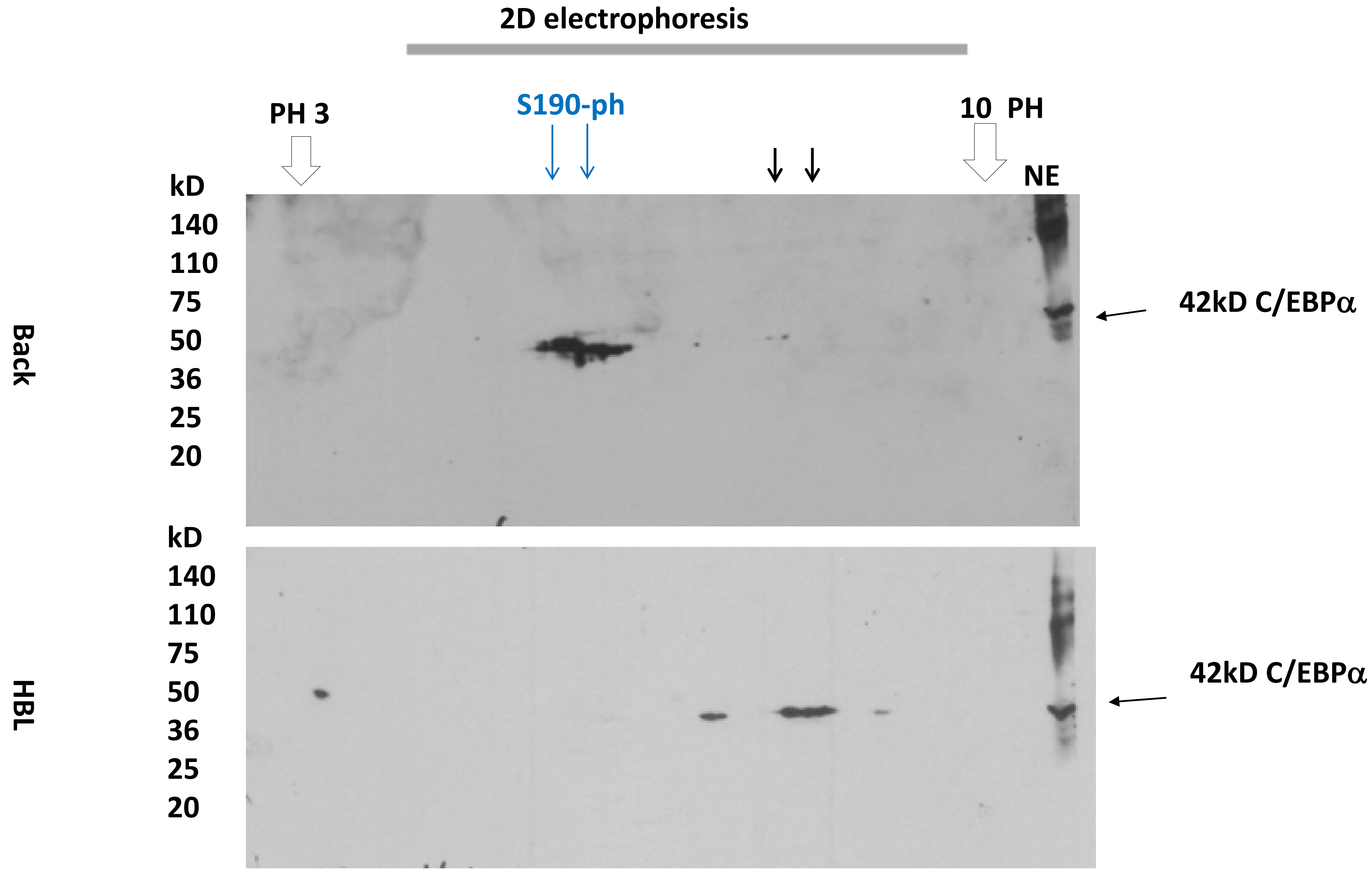

Supplementary Fig 25. Full-length immunoblots corresponding to the blots in Figure 2b.

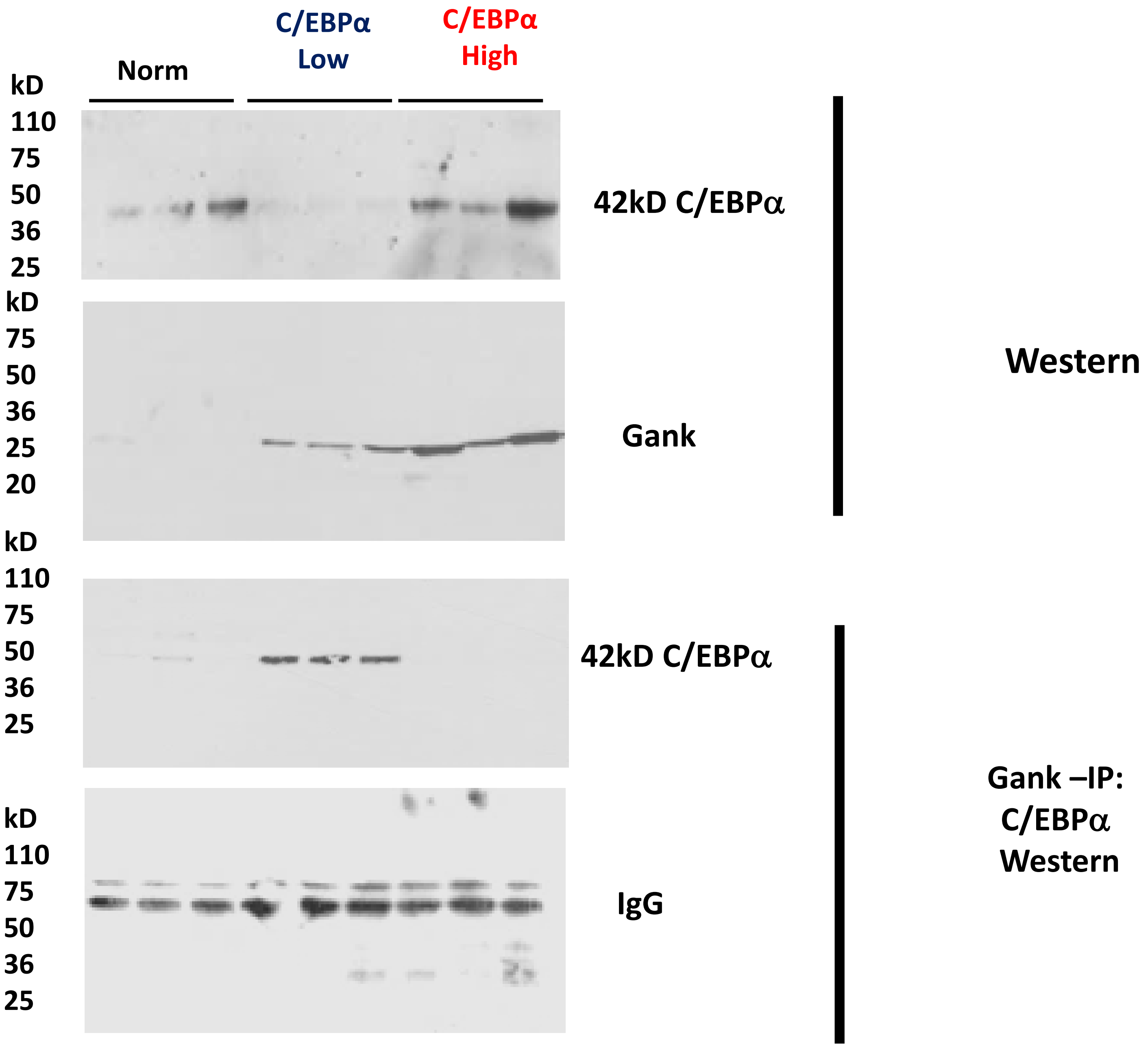

Supplementary Fig 26. Full-length immunoblots corresponding to the blots in Figure 2c.

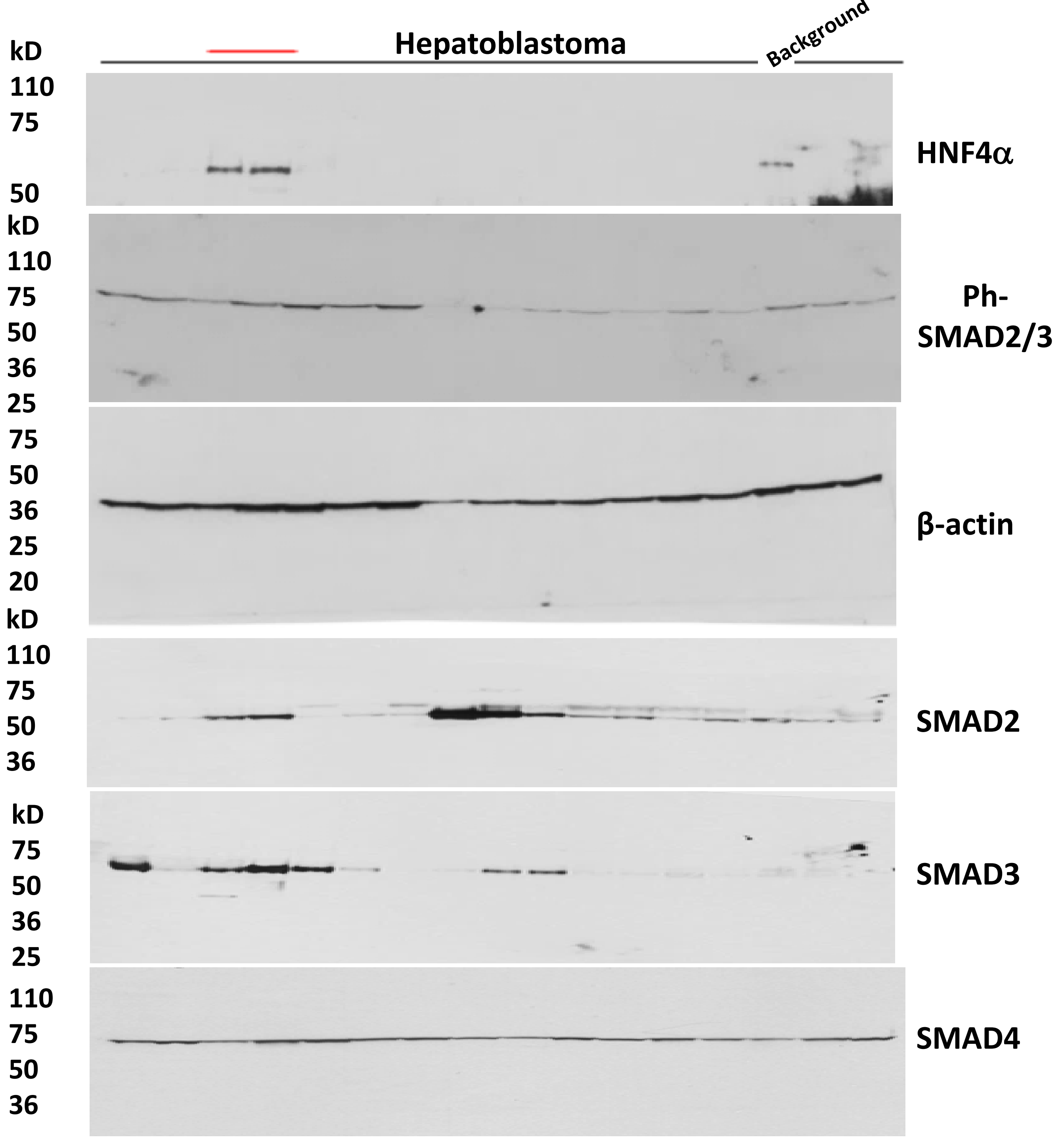

Supplementary Fig 27. Full-length immunoblots corresponding to the blots in Figure 2d.

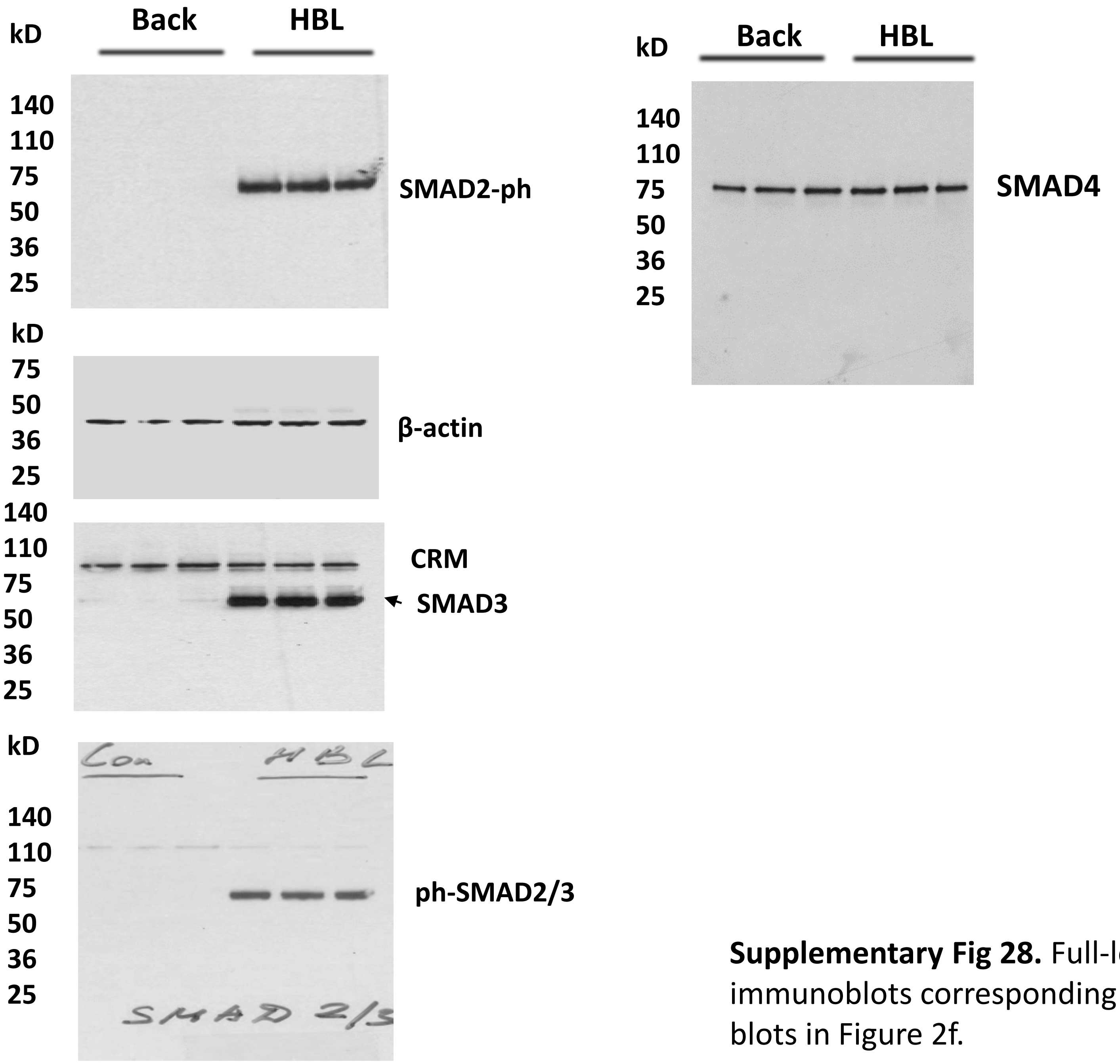

Supplementary Fig 28. Full-length immunoblots corresponding to the blots in Figure 2f.

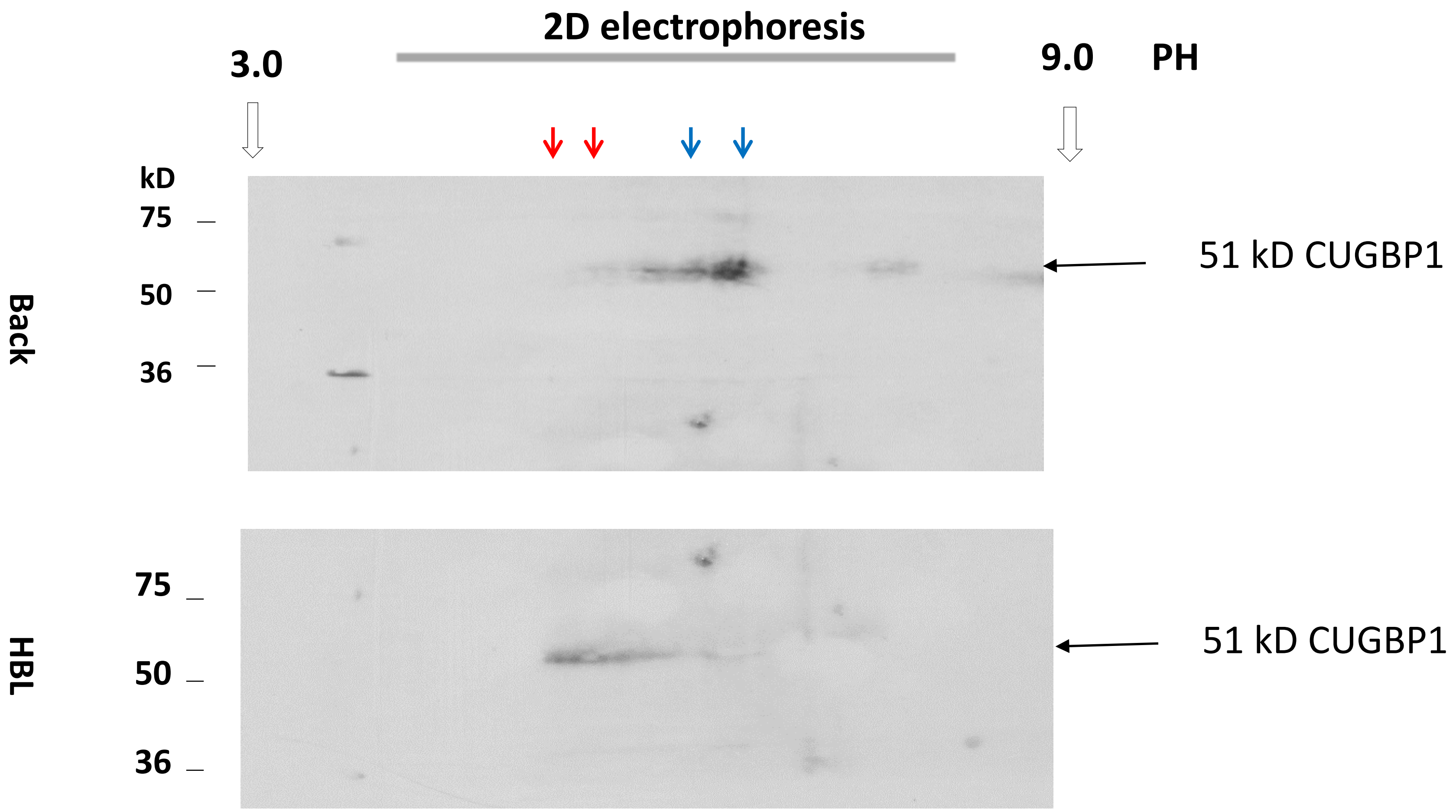

Supplementary Fig 29. Full-length immunoblots corresponding to the blots in Figure 2i.

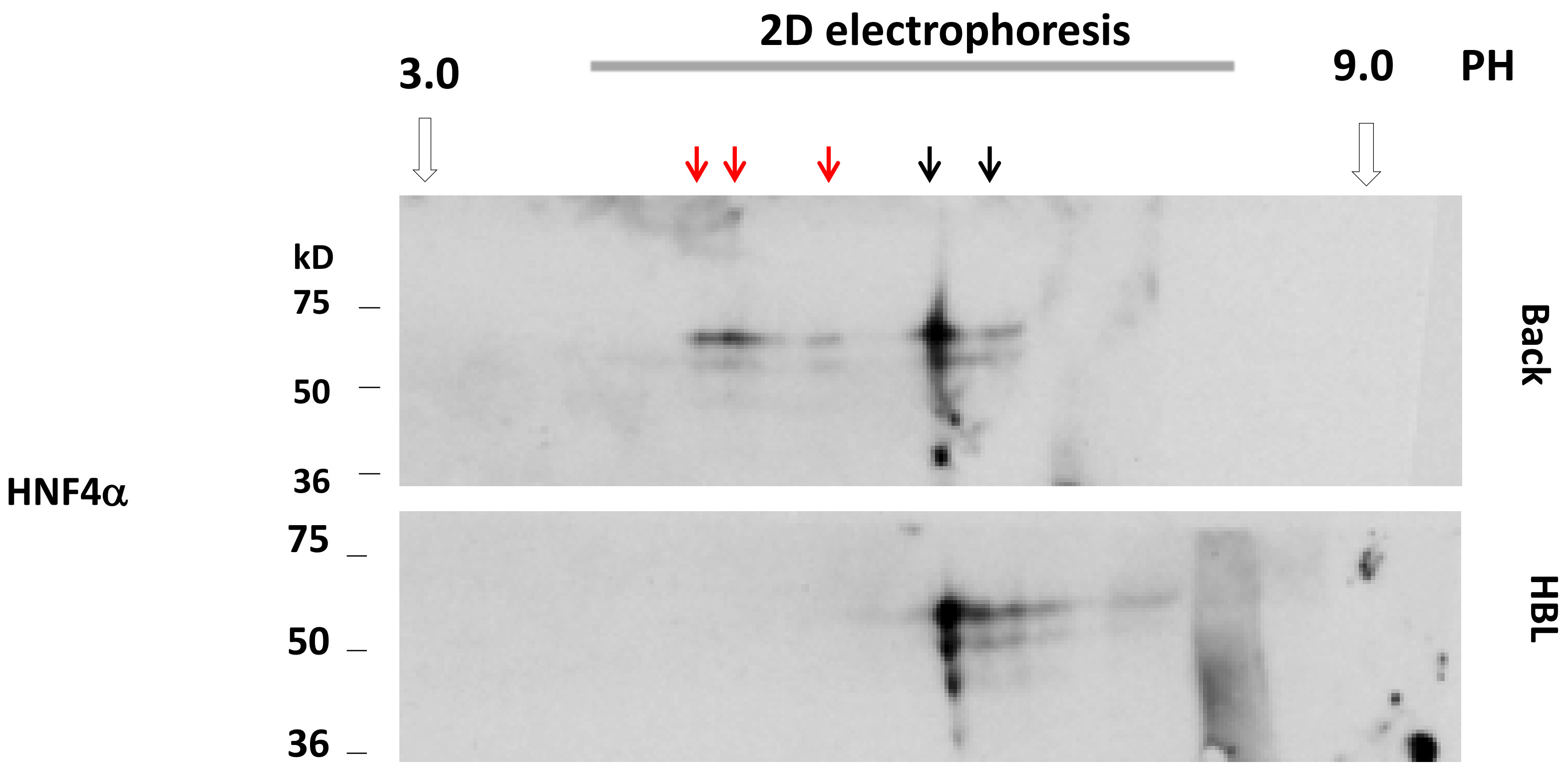

Supplementary Fig 30. Full-length immunoblots corresponding to the blots in Figure 2h.

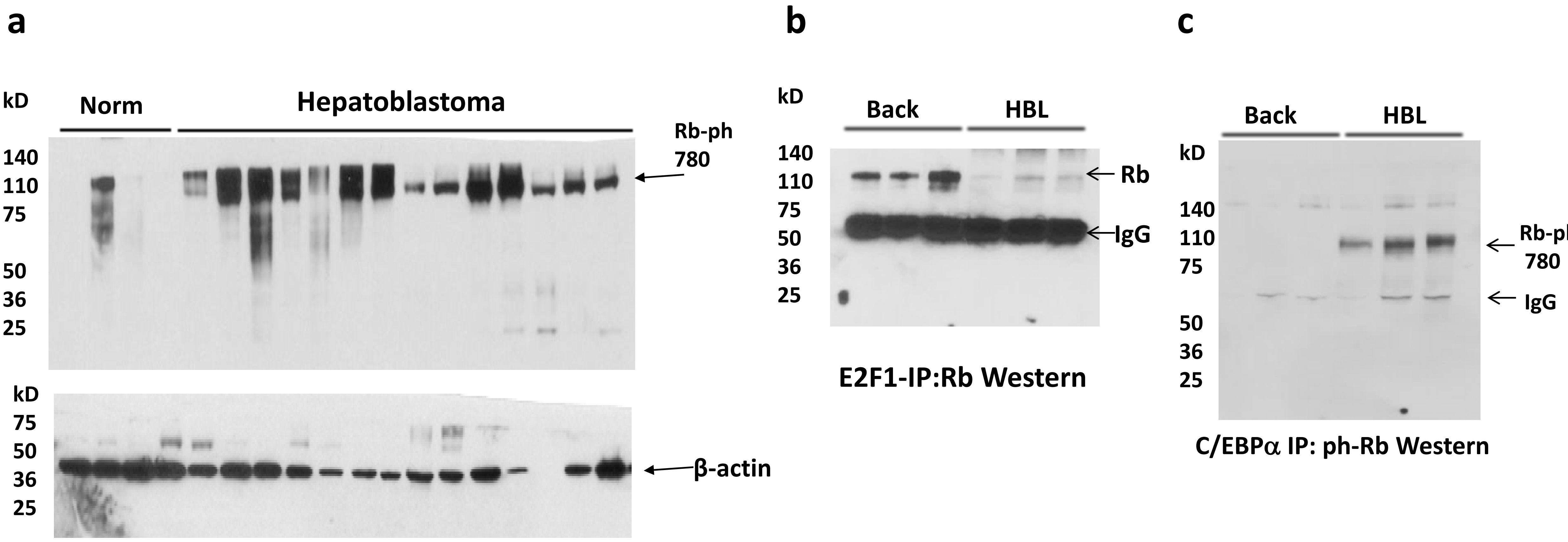

Supplementary Fig 31. Full-length immunoblots corresponding to the blots in Figure 3a, b and c.

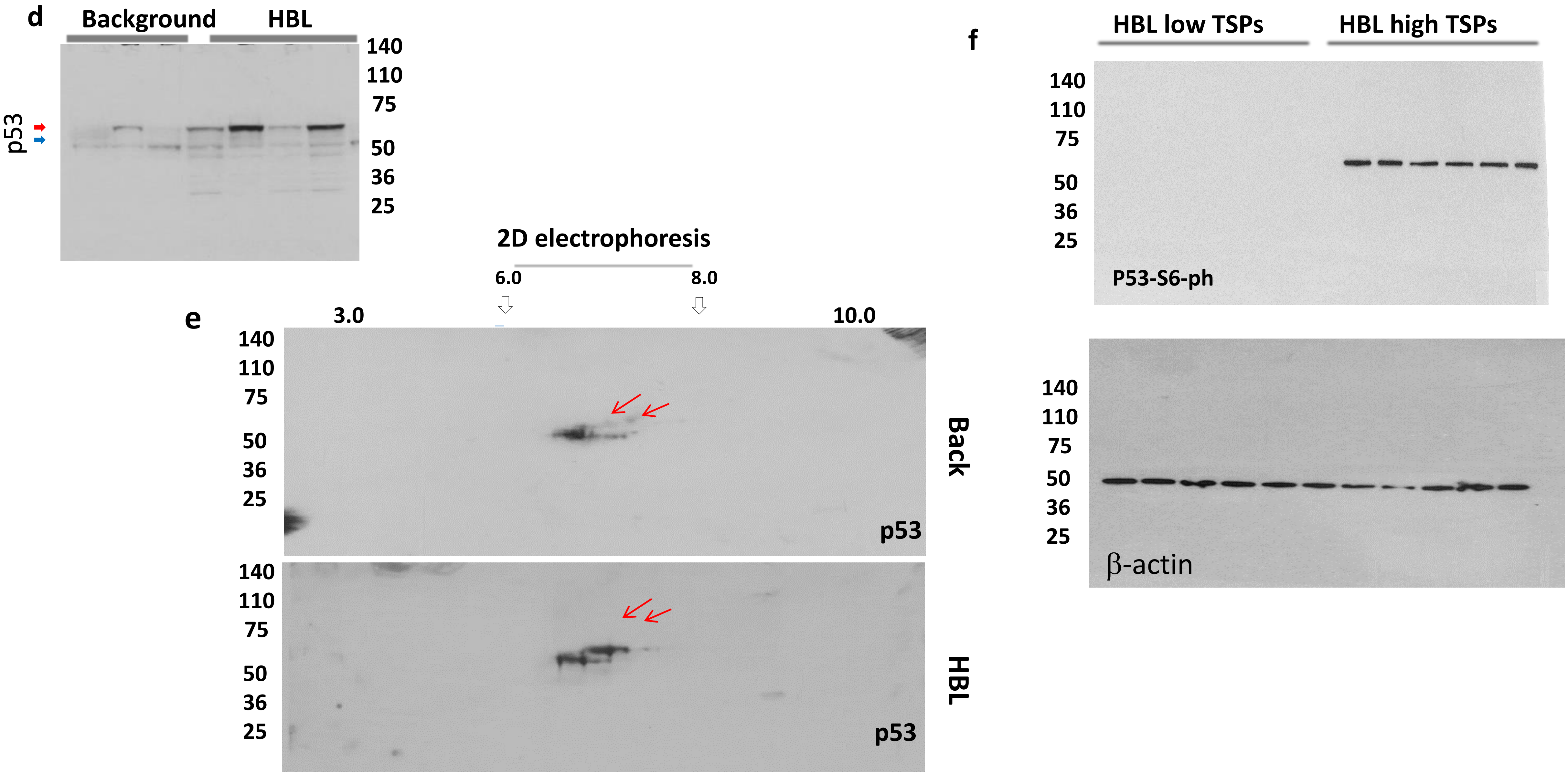

Supplementary Fig 32. Full-length immunoblots corresponding to the blots in Figure 3d, e and f.

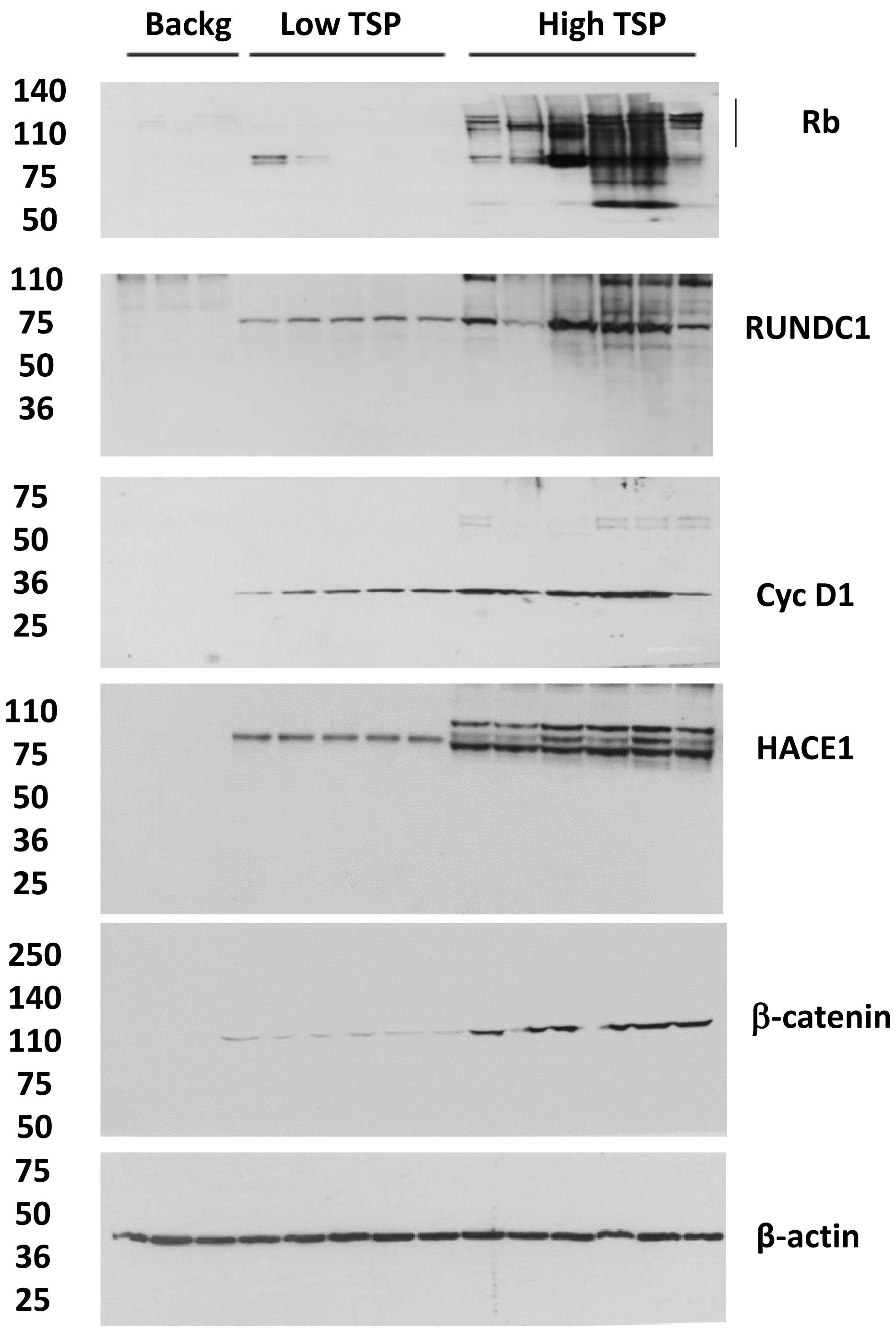

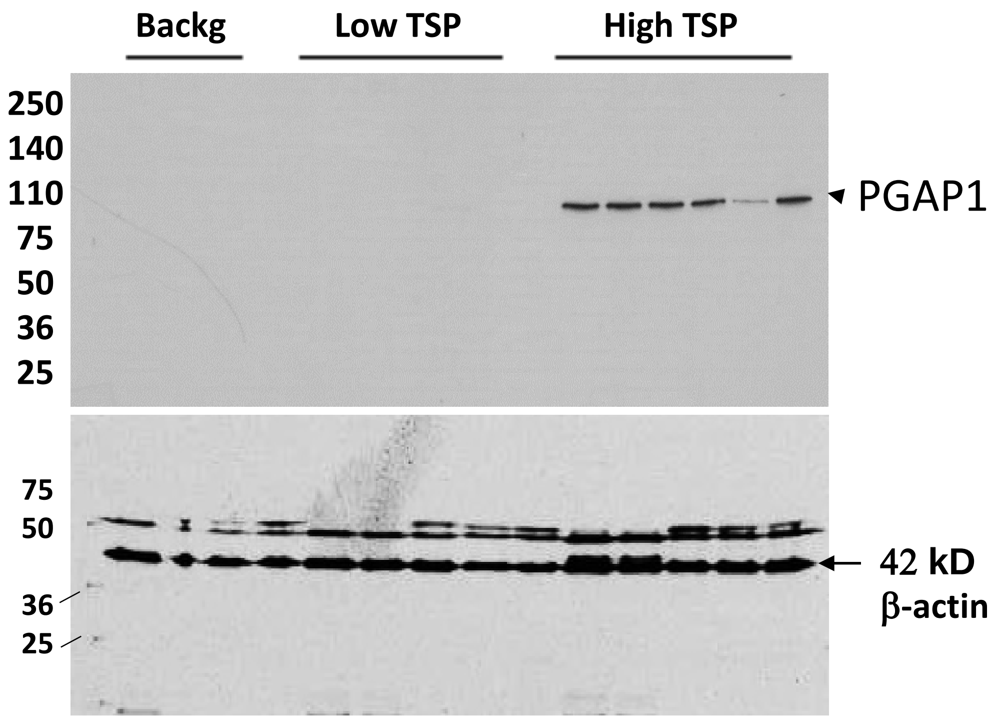

Supplementary Fig 33. Full-length immunoblots corresponding to the blots in Figure 4f. Note that Western blotting for PGAP1 and corresponding β-actin control are performed with a separate gel.

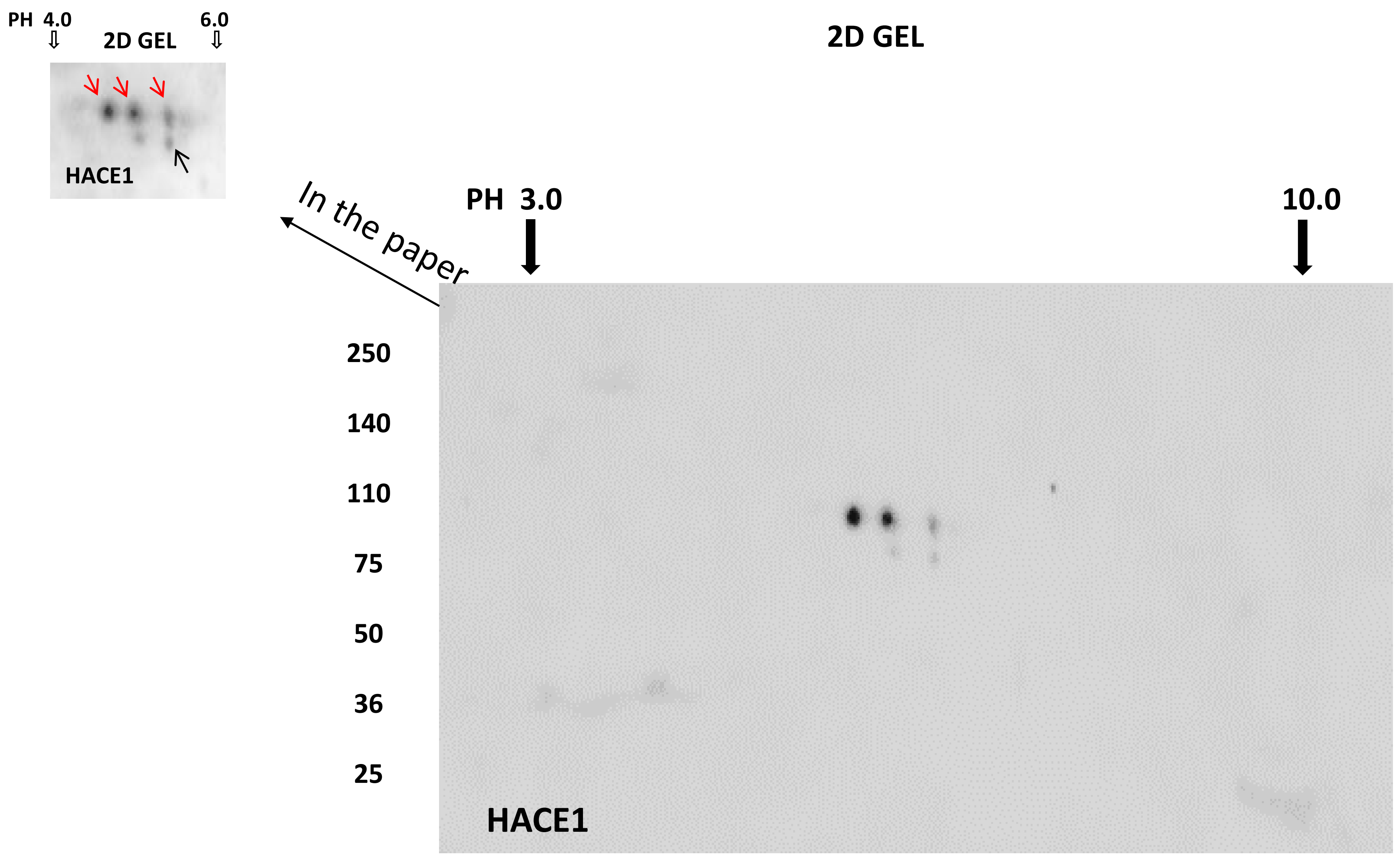

Supplementary Fig 34. Full-length immunoblots corresponding to the blots in Figure 4g.

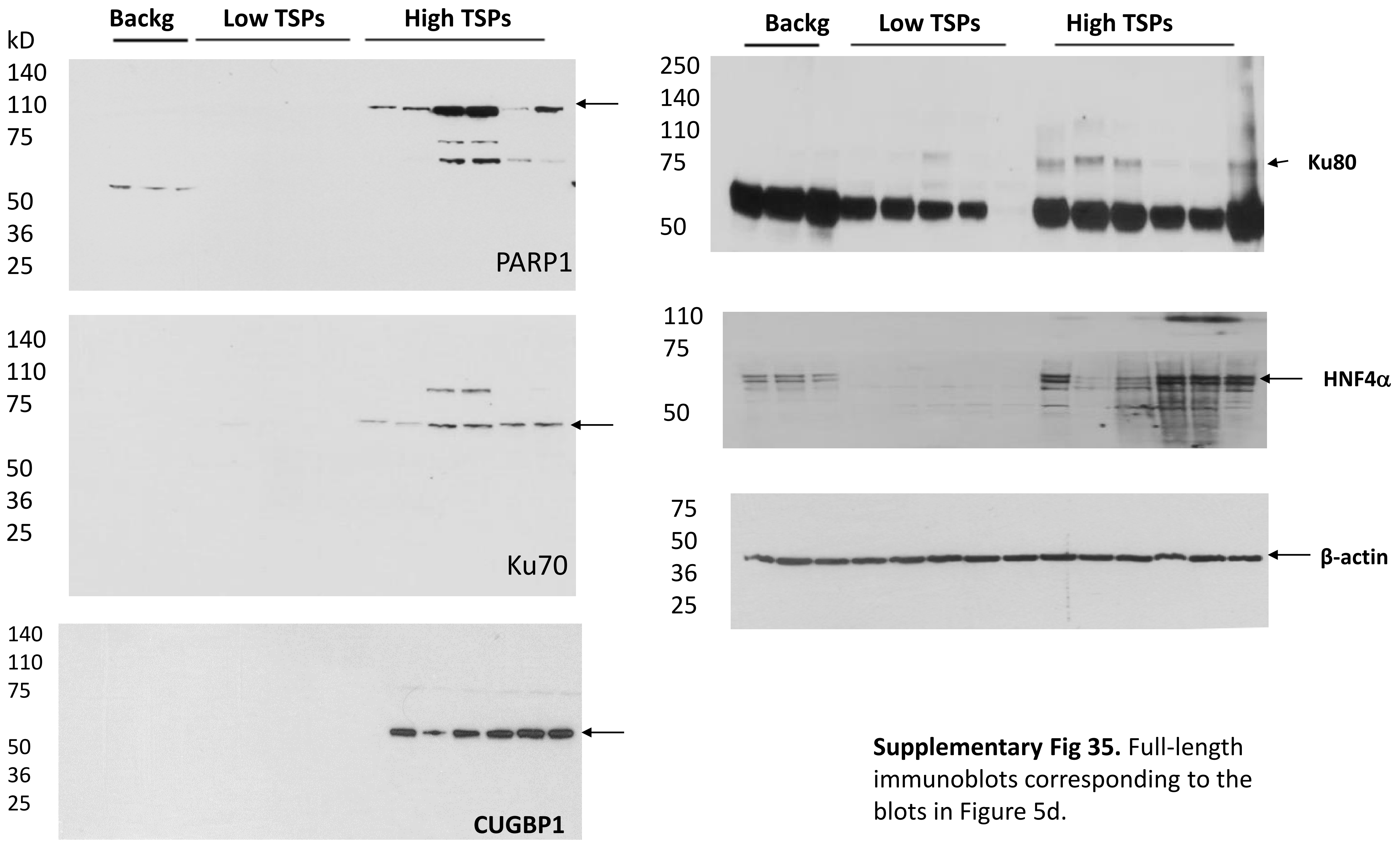

Supplementary Fig 35. Full-length immunoblots corresponding to the blots in Figure 5d.

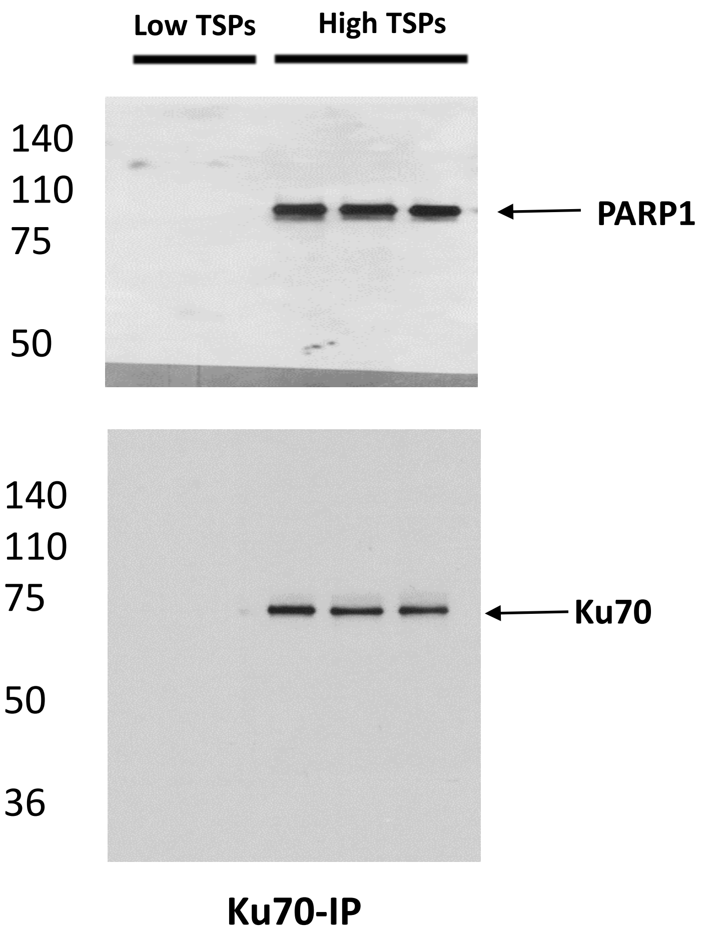

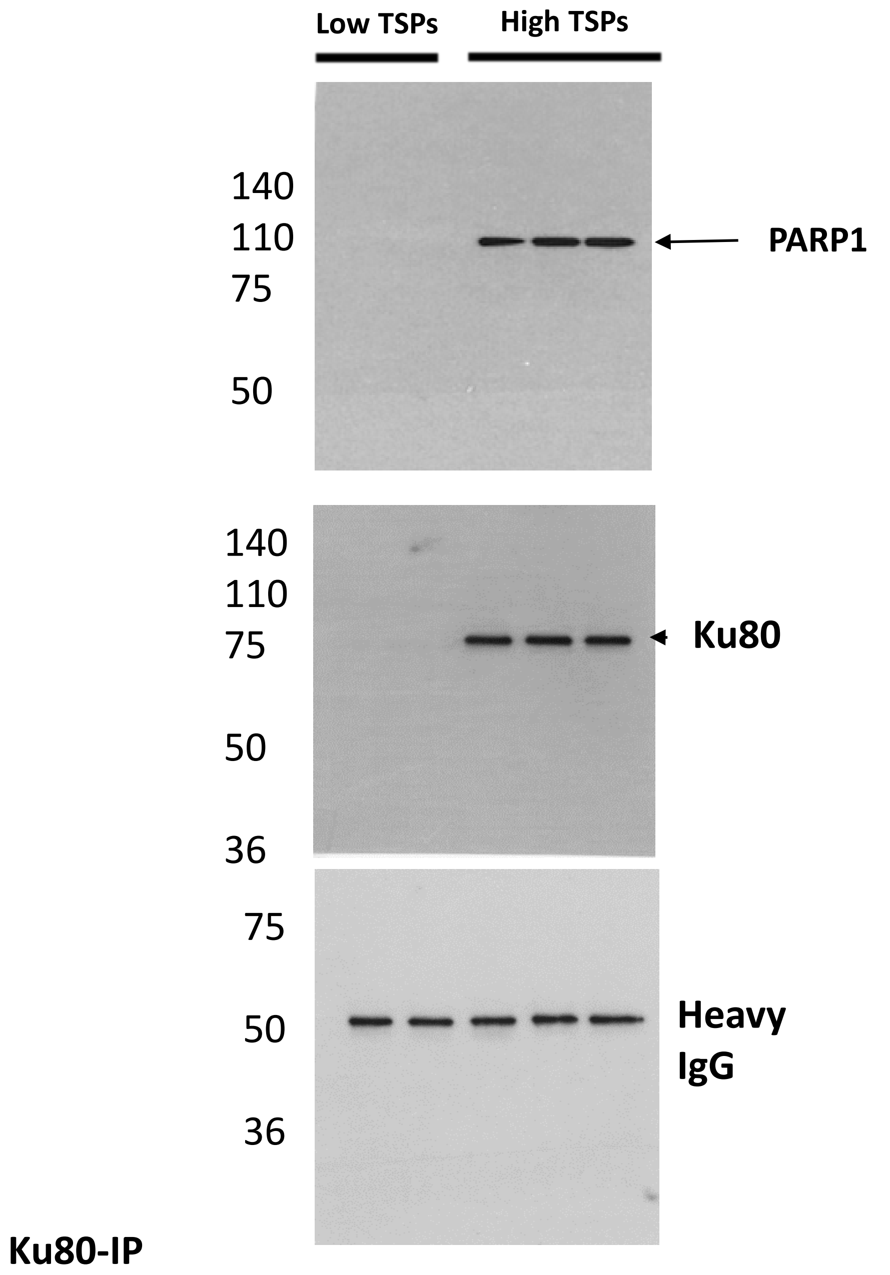

Supplementary Fig 36. Full-length immunoblots corresponding to the blots in Figure 5e.

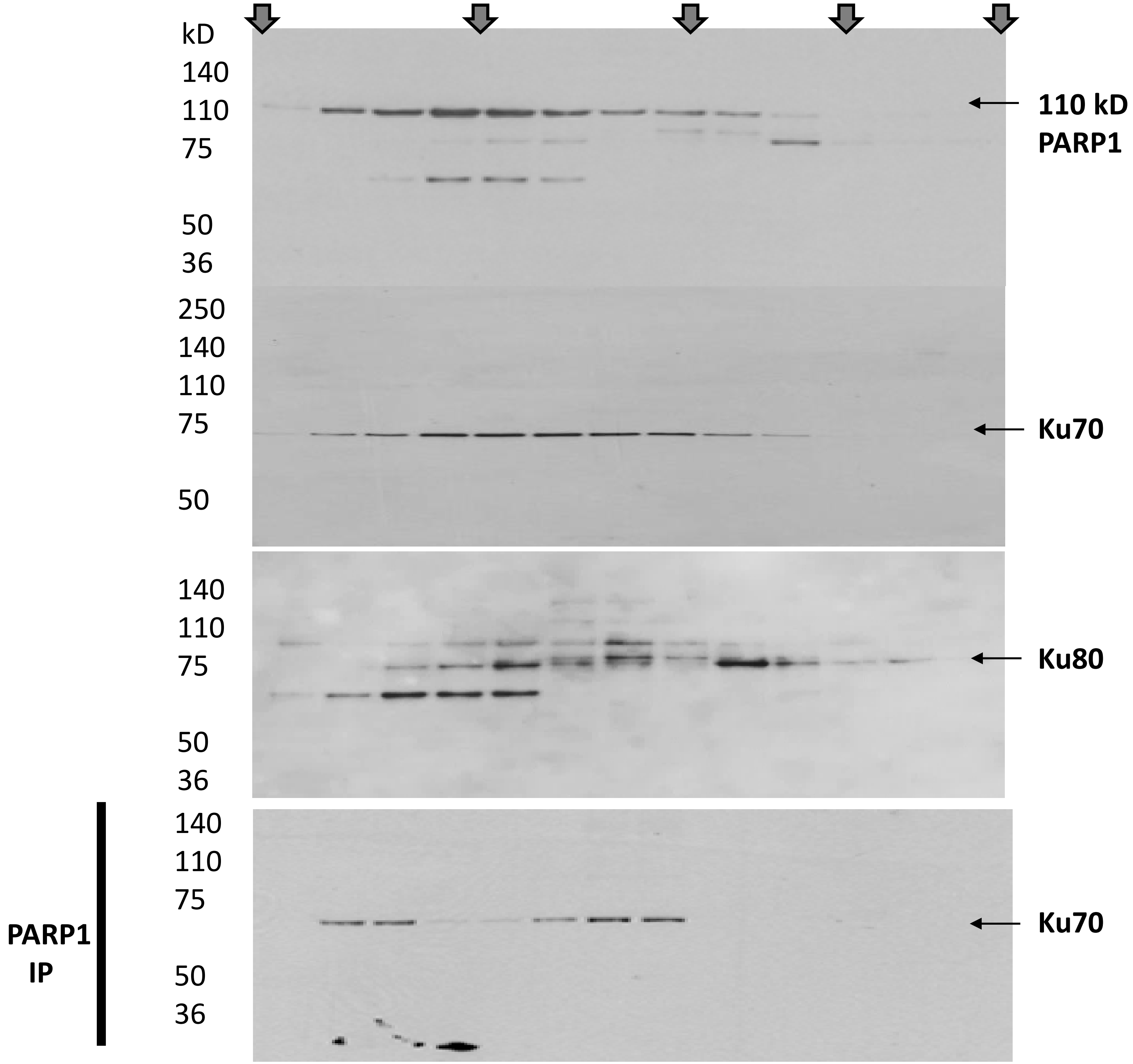

Supplementary Fig 37. Full-length immunoblots corresponding to the blots in Figure 5g.

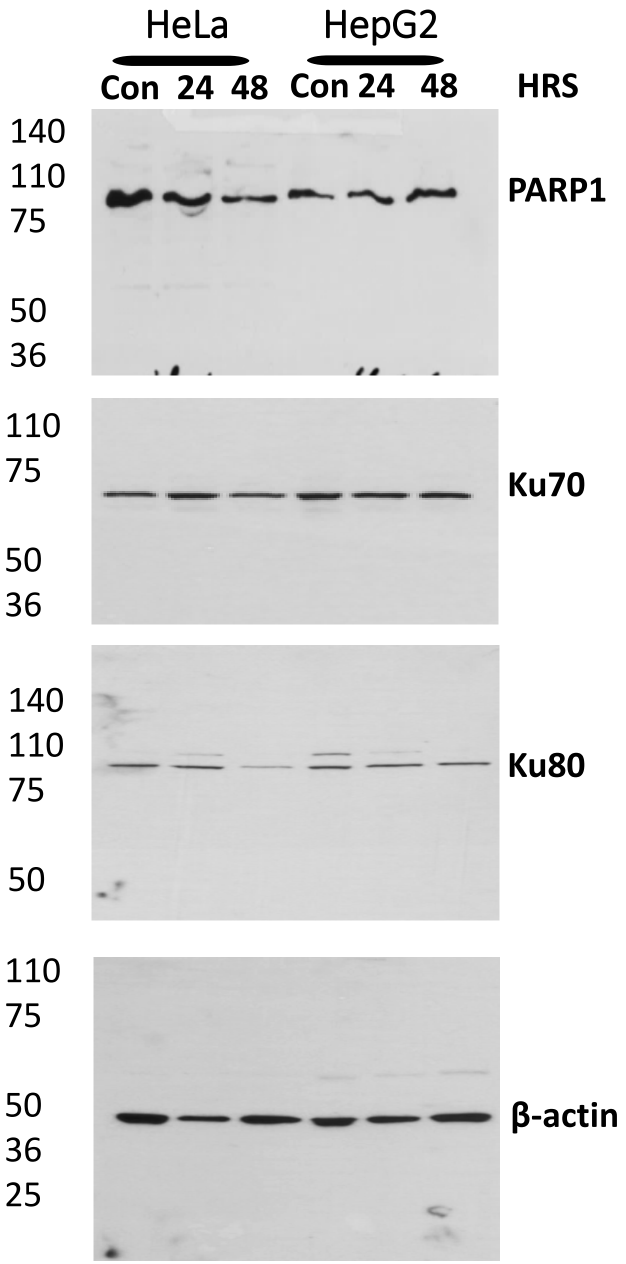

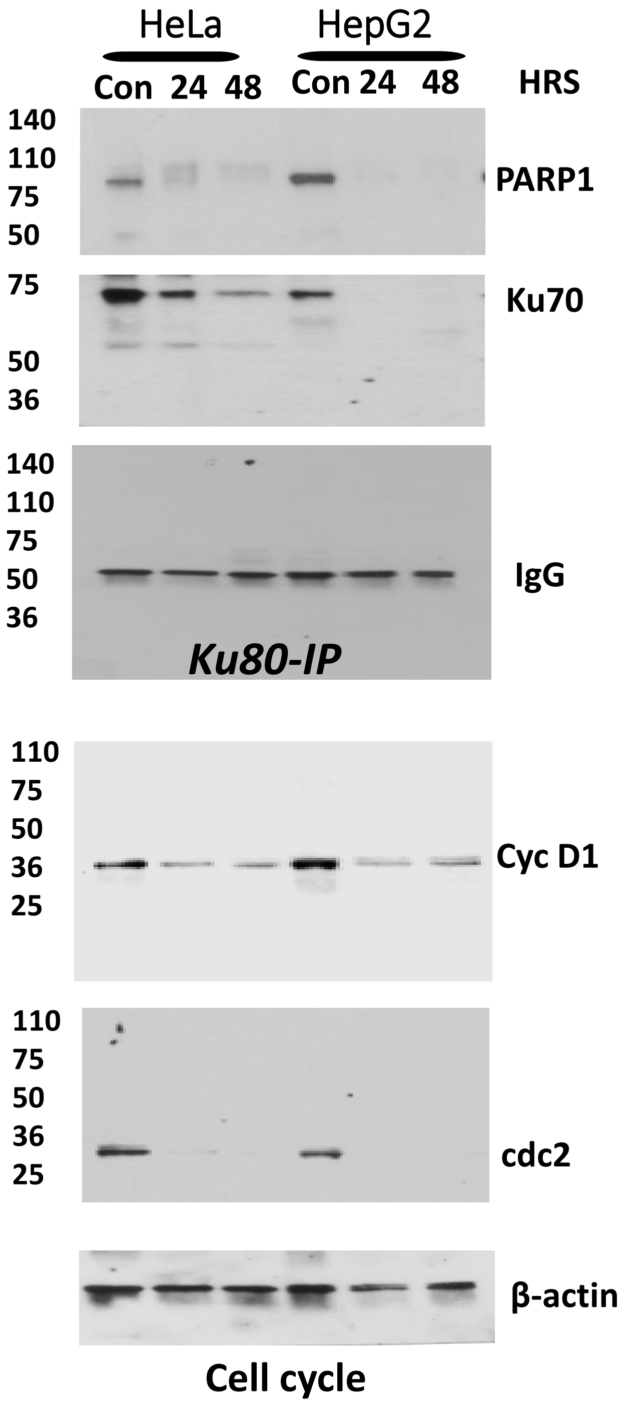

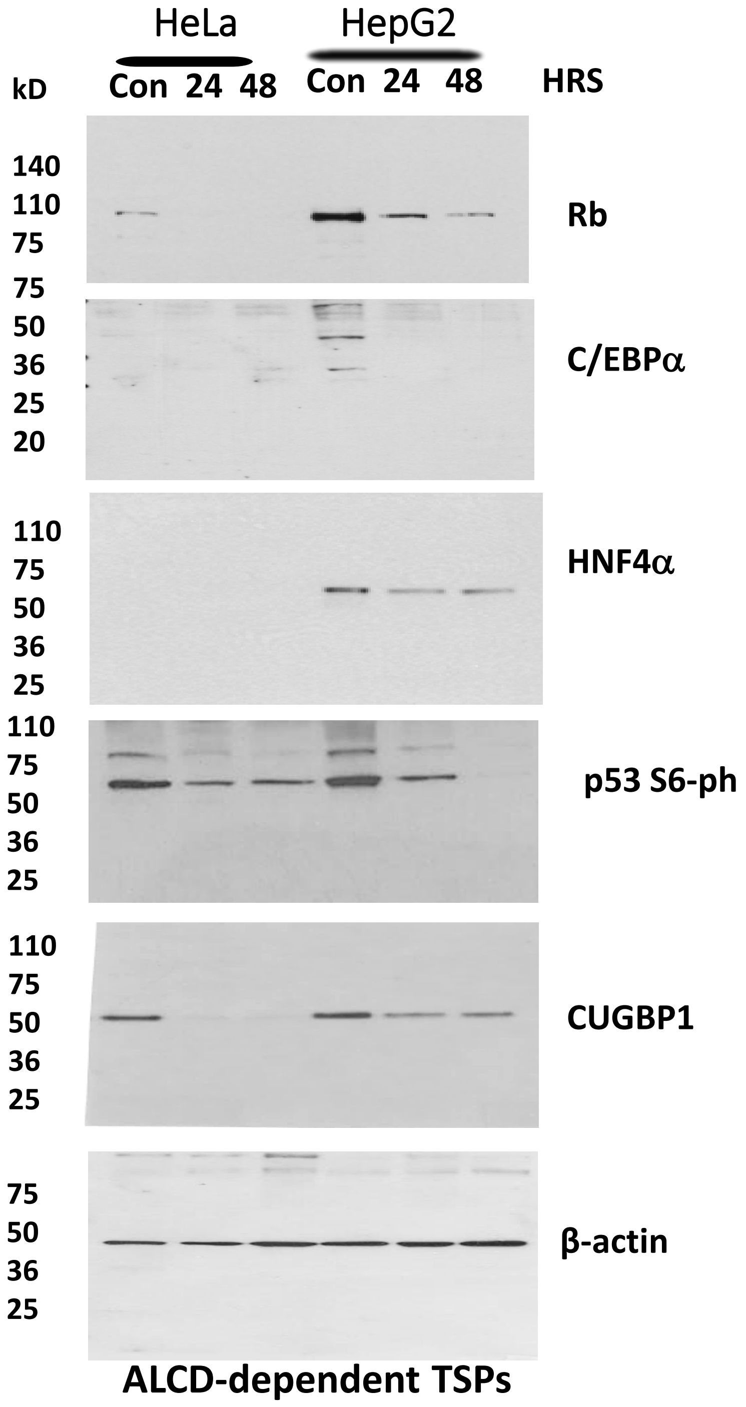

Supplementary Fig 38. Full-length immunoblots corresponding to the blots in Figure 6f.

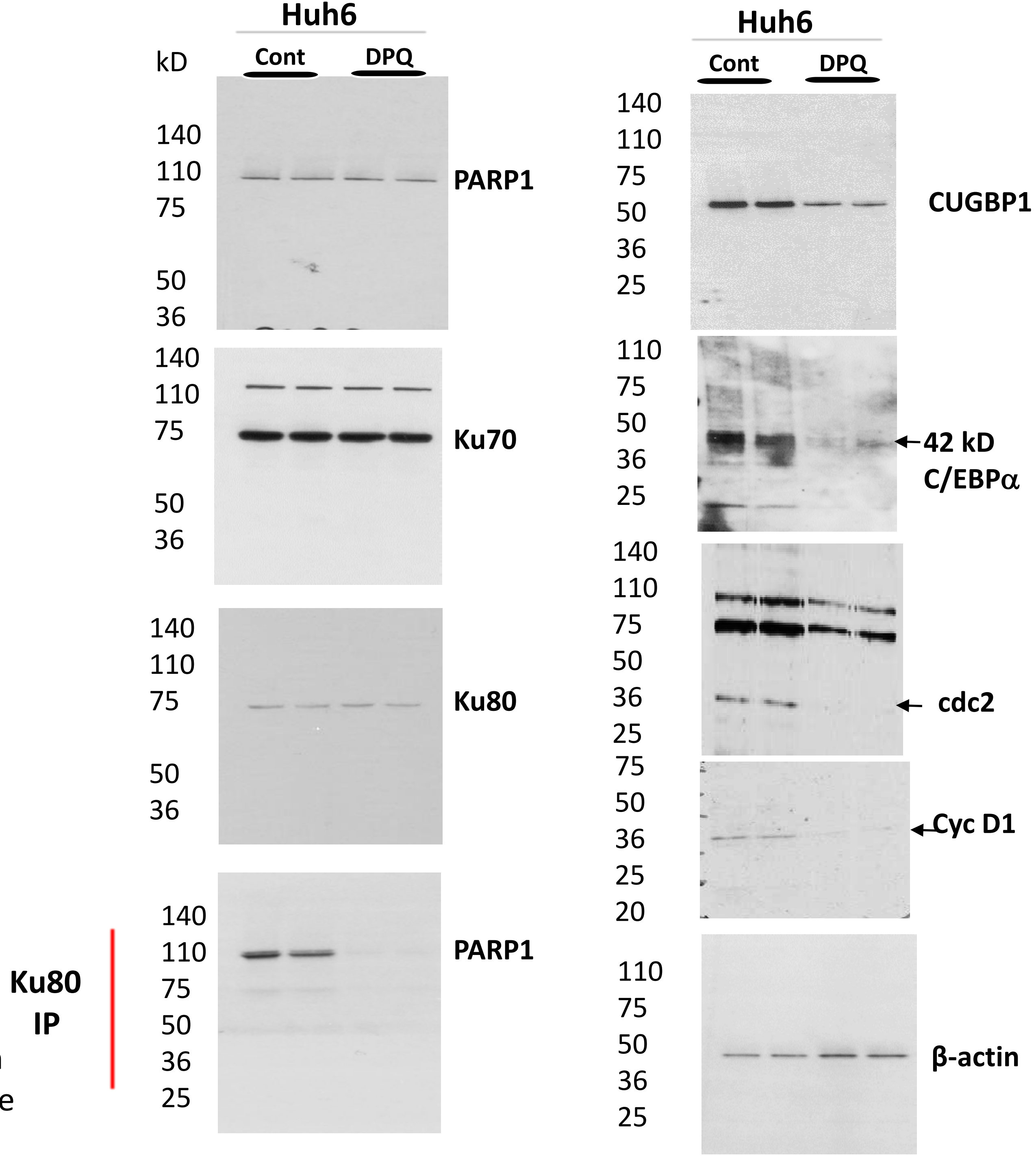

Supplementary Fig 39. Full-length immunoblots corresponding to the blots in Figure 7a.

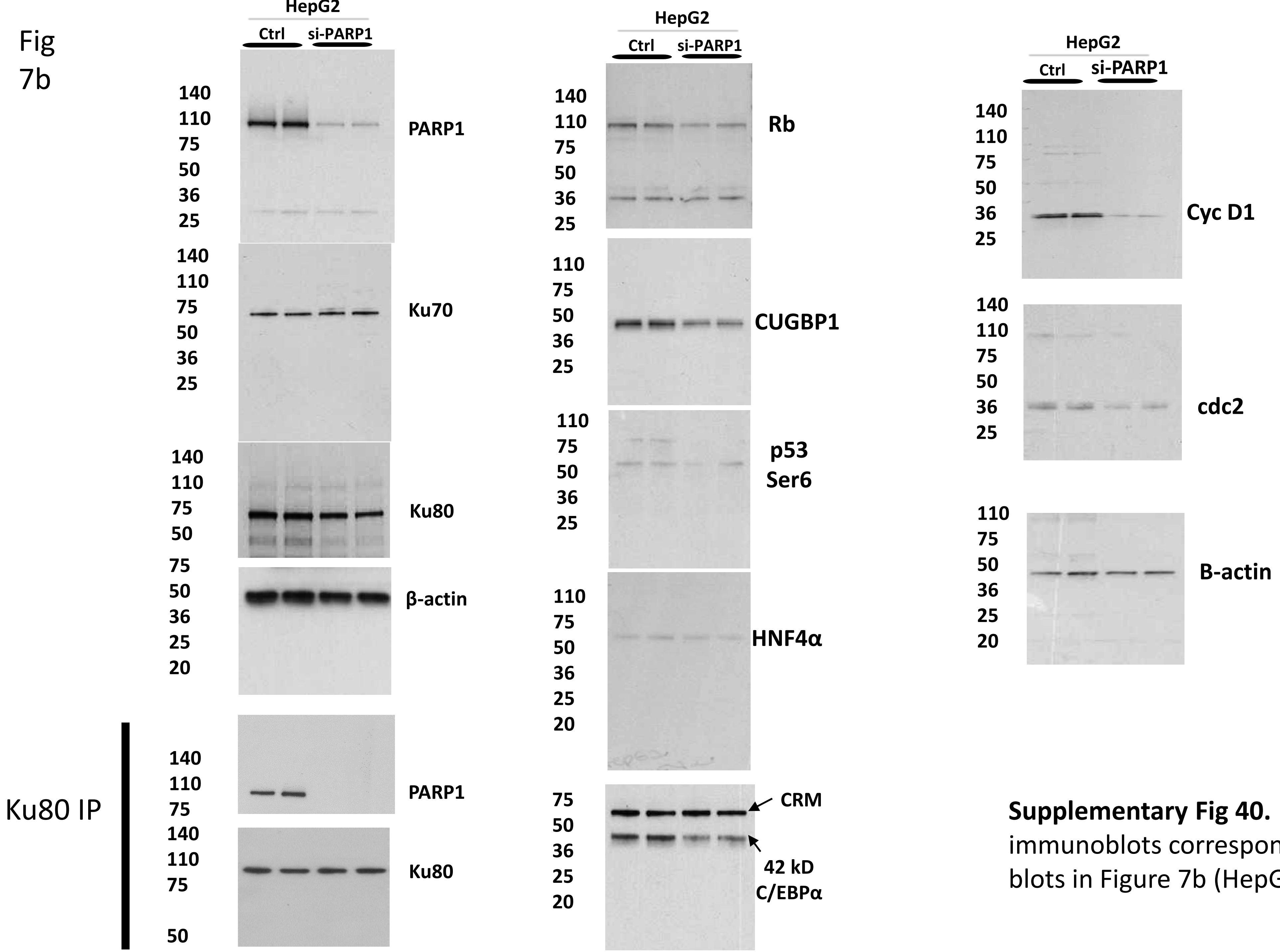

Supplementary Fig 40. Full-length immunoblots corresponding to the blots in Figure 7b (HepG2 cells).

Fig 7b, Huh6 cells

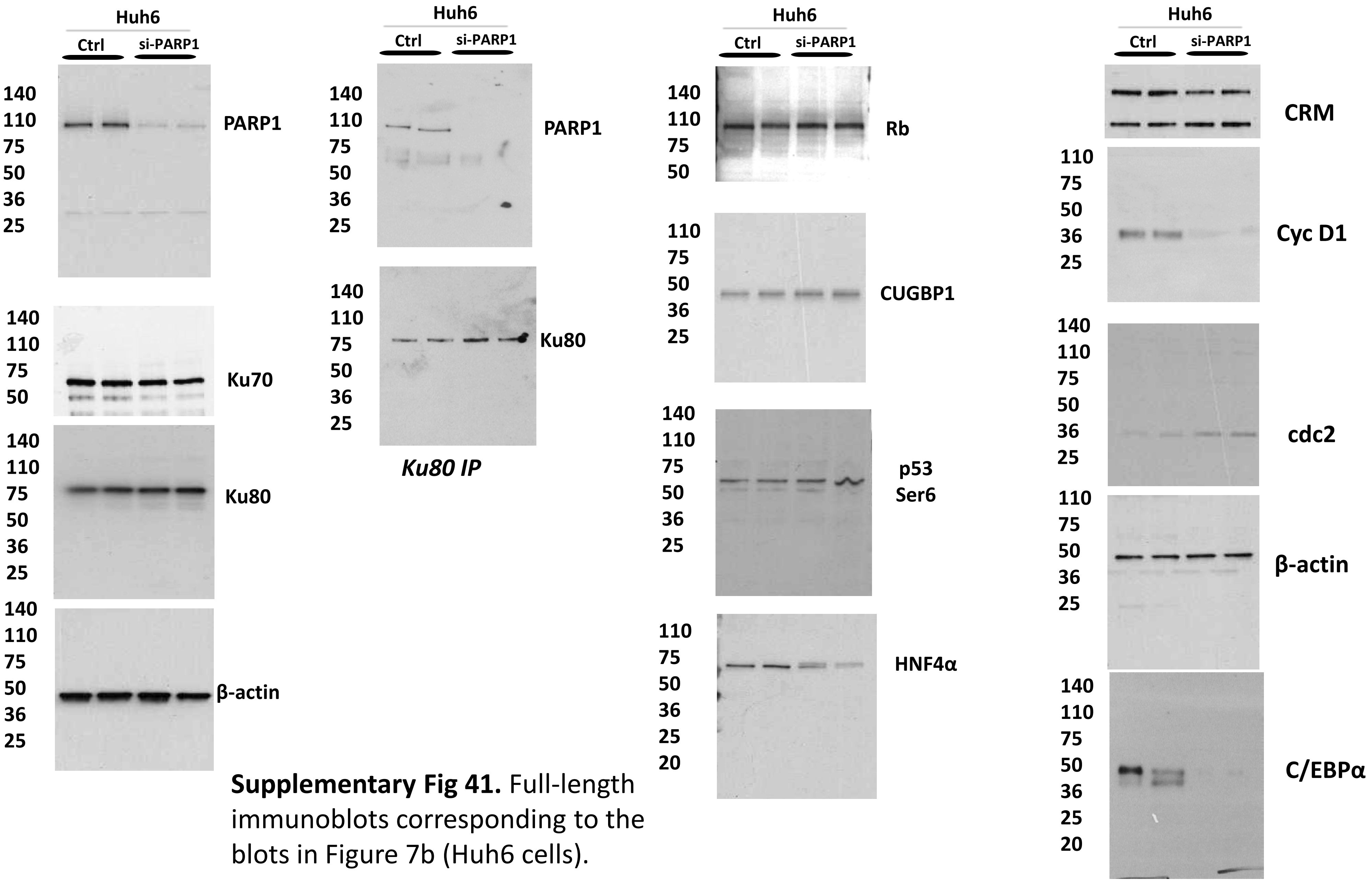

Supplementary Fig 41. Full-length immunoblots corresponding to the blots in Figure 7b (Huh6 cells).

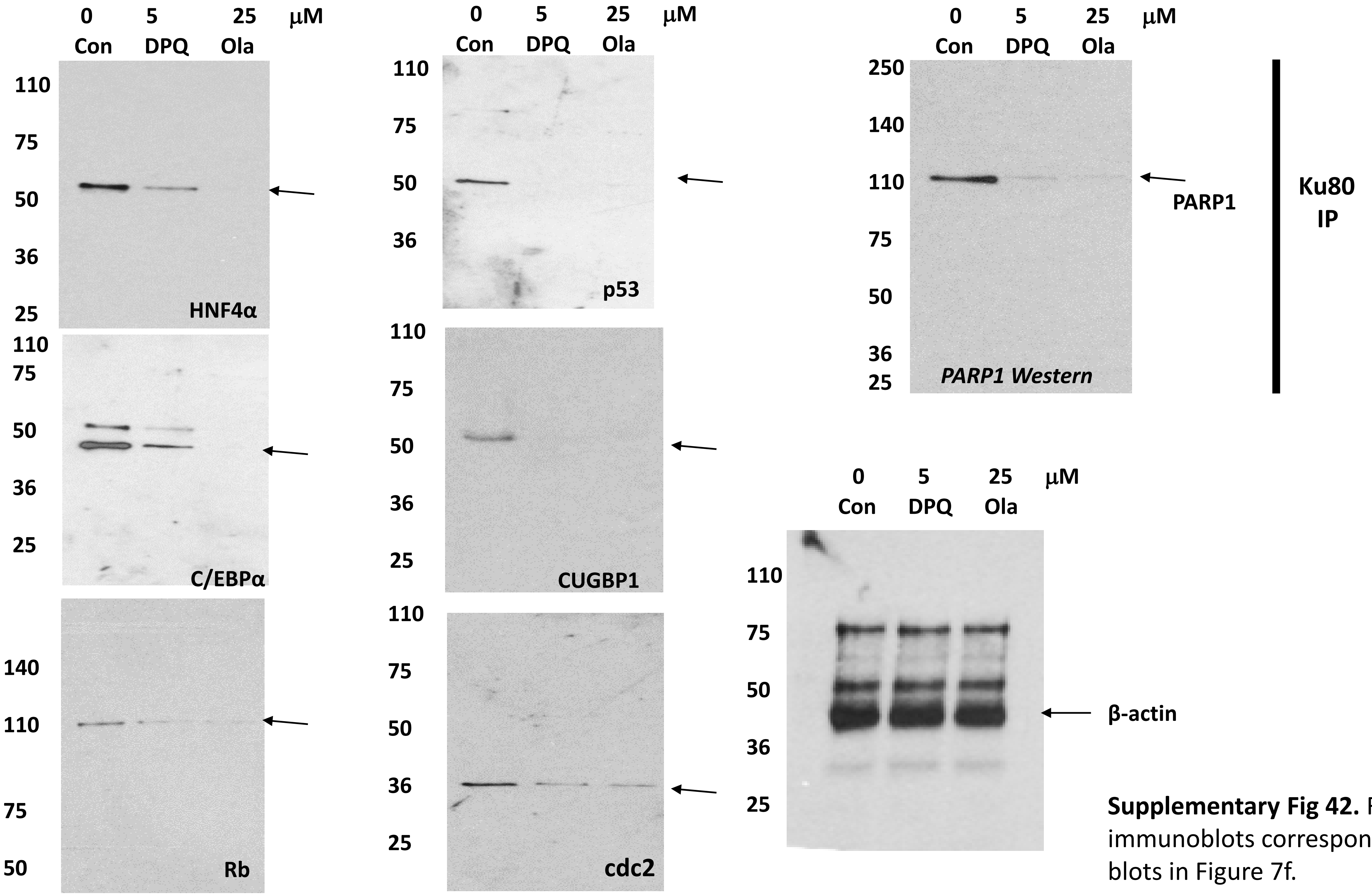

Supplementary Fig 42. Full-length immunoblots corresponding to the blots in Figure 7f.
